# Supplementary figures and images for: The conditioned medium from mesenchymal stromal cells pretreated with proinflammatory cytokines promote fibroblasts migration and activation
Source: PLoS One. 2022 Apr 11;17(4):e0265049. doi: 10.1371/journal.pone.0265049 (PMC9000110; doi:10.1371/journal.pone.0265049)

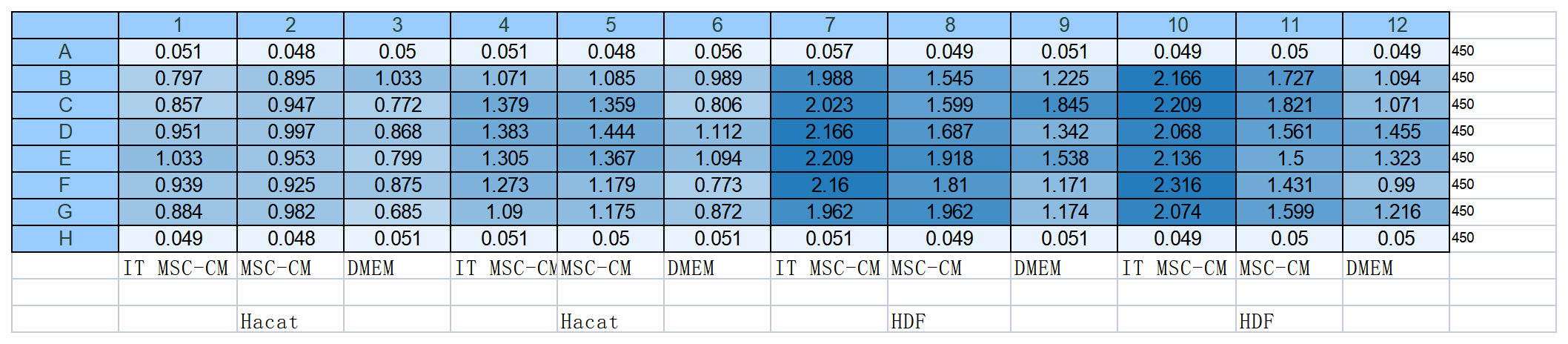

Supplement: S1 Fig — (TIF) [file pone.0265049.s001.tif]

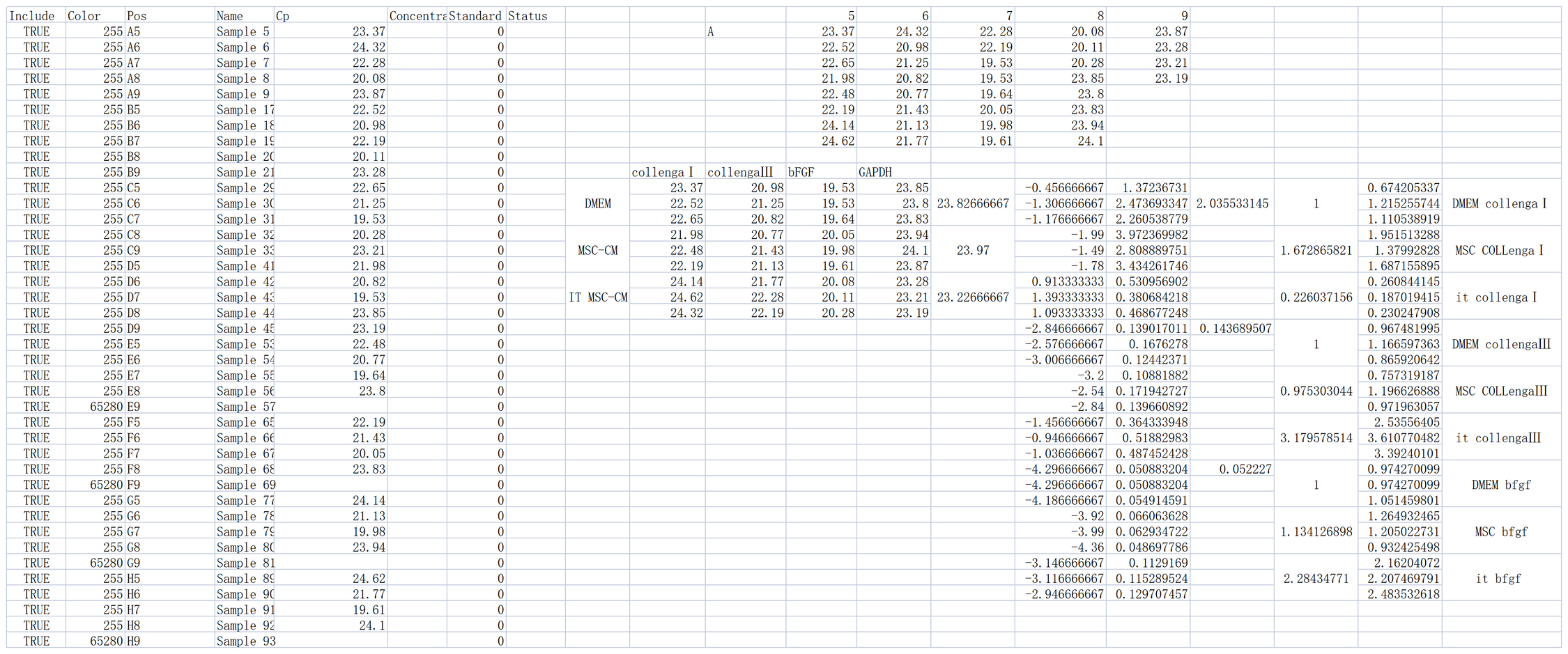

Supplement: S2 Fig — (TIF) [file pone.0265049.s002.tif]

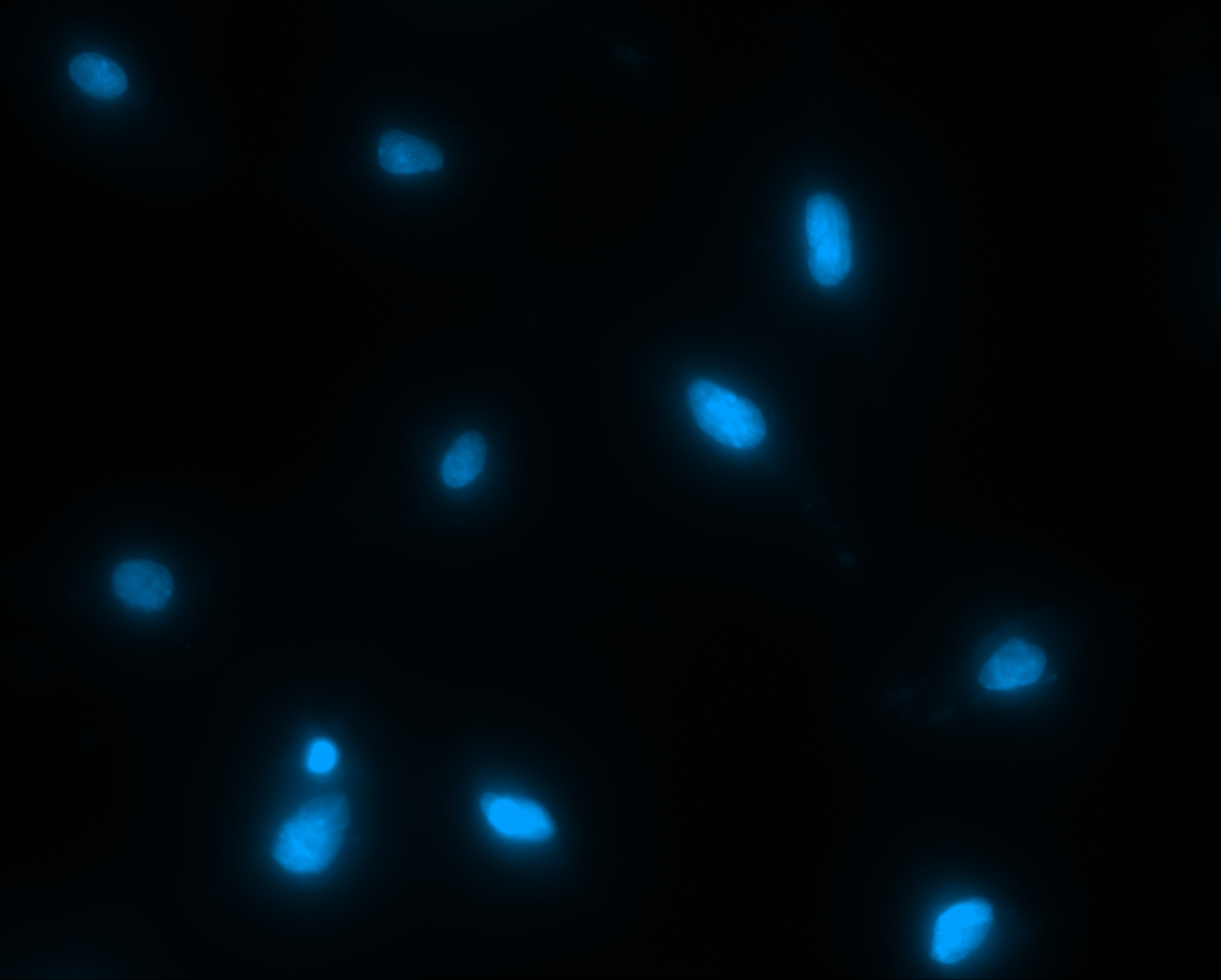

Supplement: S1 File — (ZIP) [file pone.0265049.s003.zip › HDF activation in vitro/DMEM Snap-317_DAPI.tif]

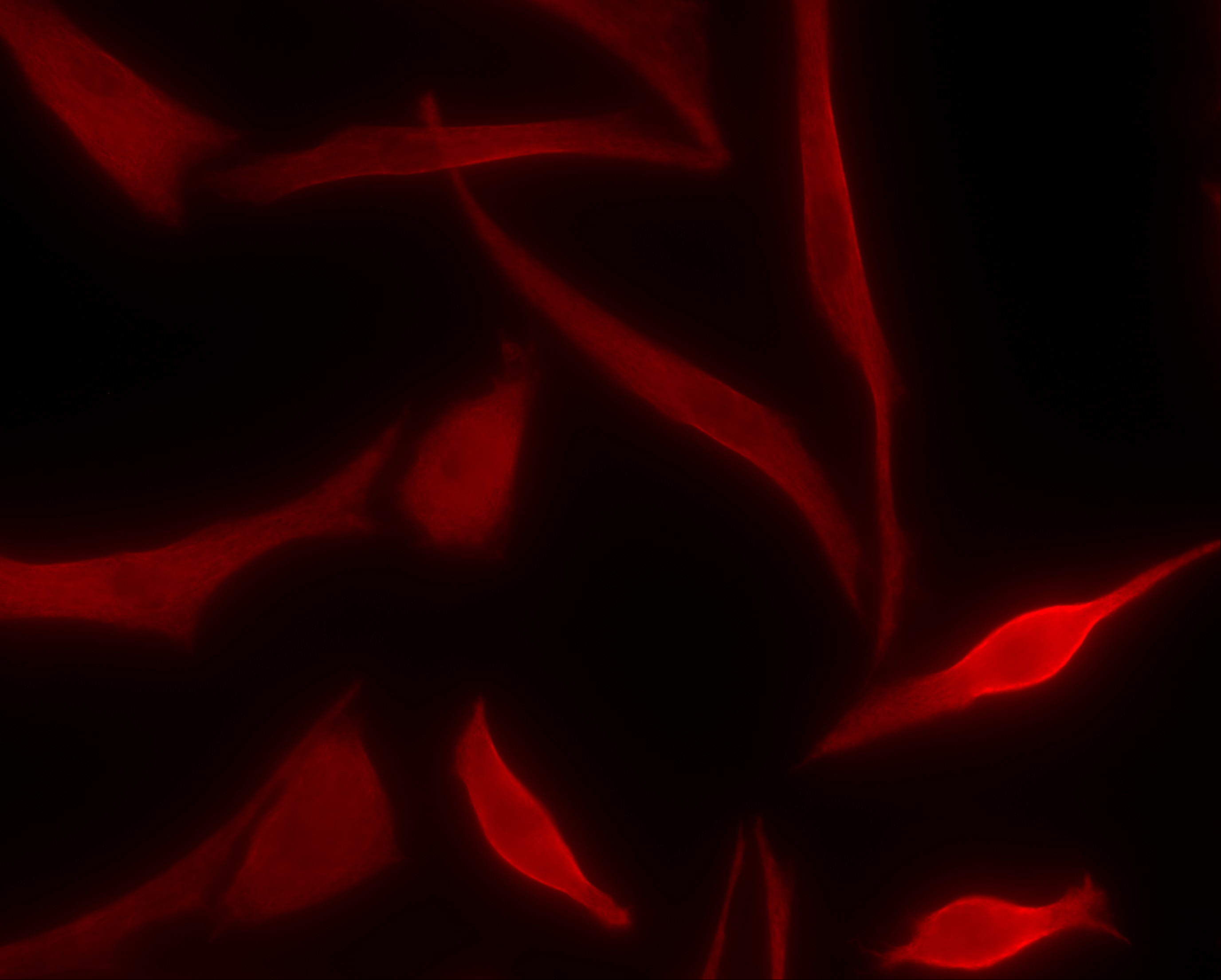

Supplement: S1 File — (ZIP) [file pone.0265049.s003.zip › HDF activation in vitro/DMEM Snap-317_Vimentin Alexa Fluor 594.tif]

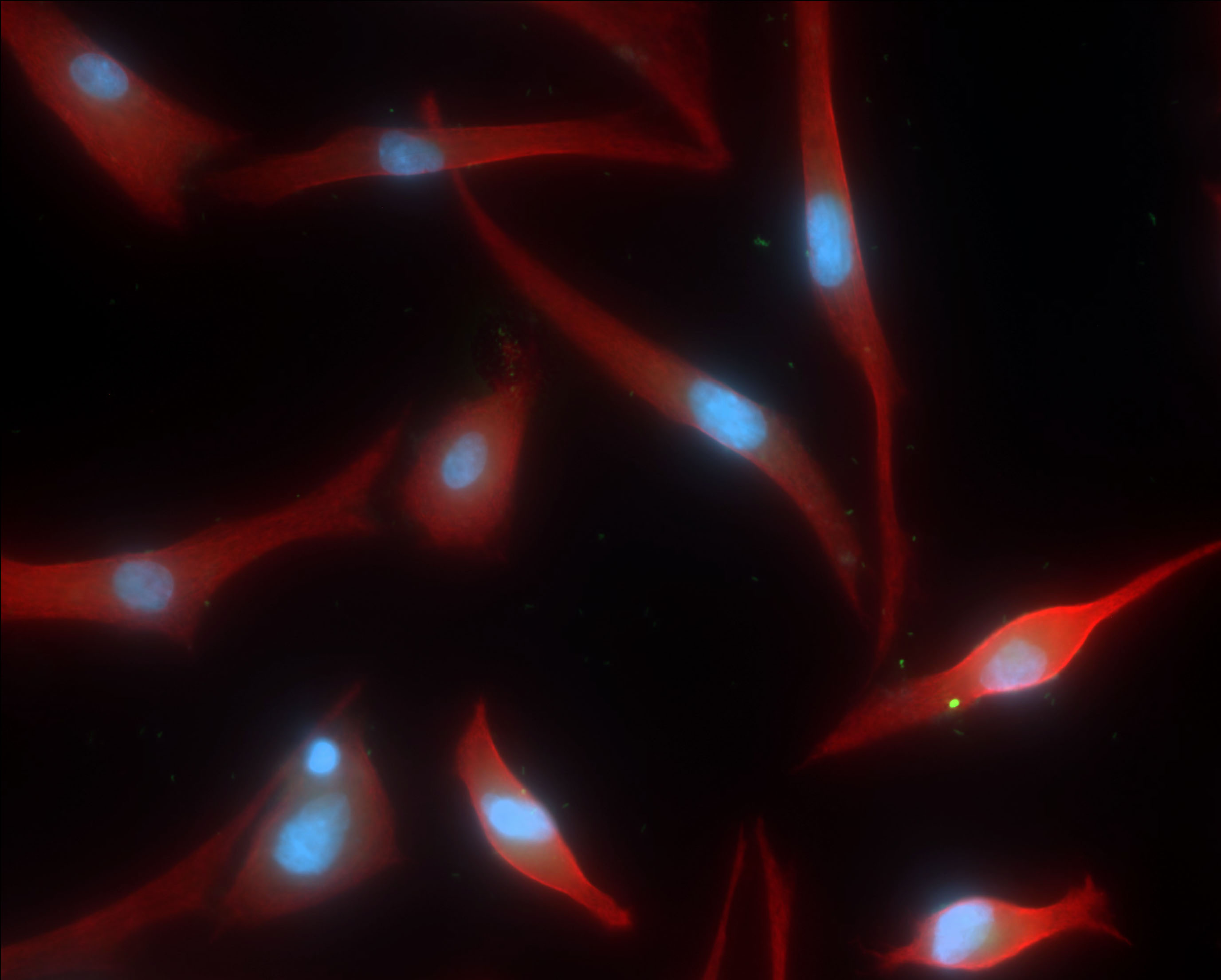

Supplement: S1 File — (ZIP) [file pone.0265049.s003.zip › HDF activation in vitro/DMEM Snap-317_merge.tif]

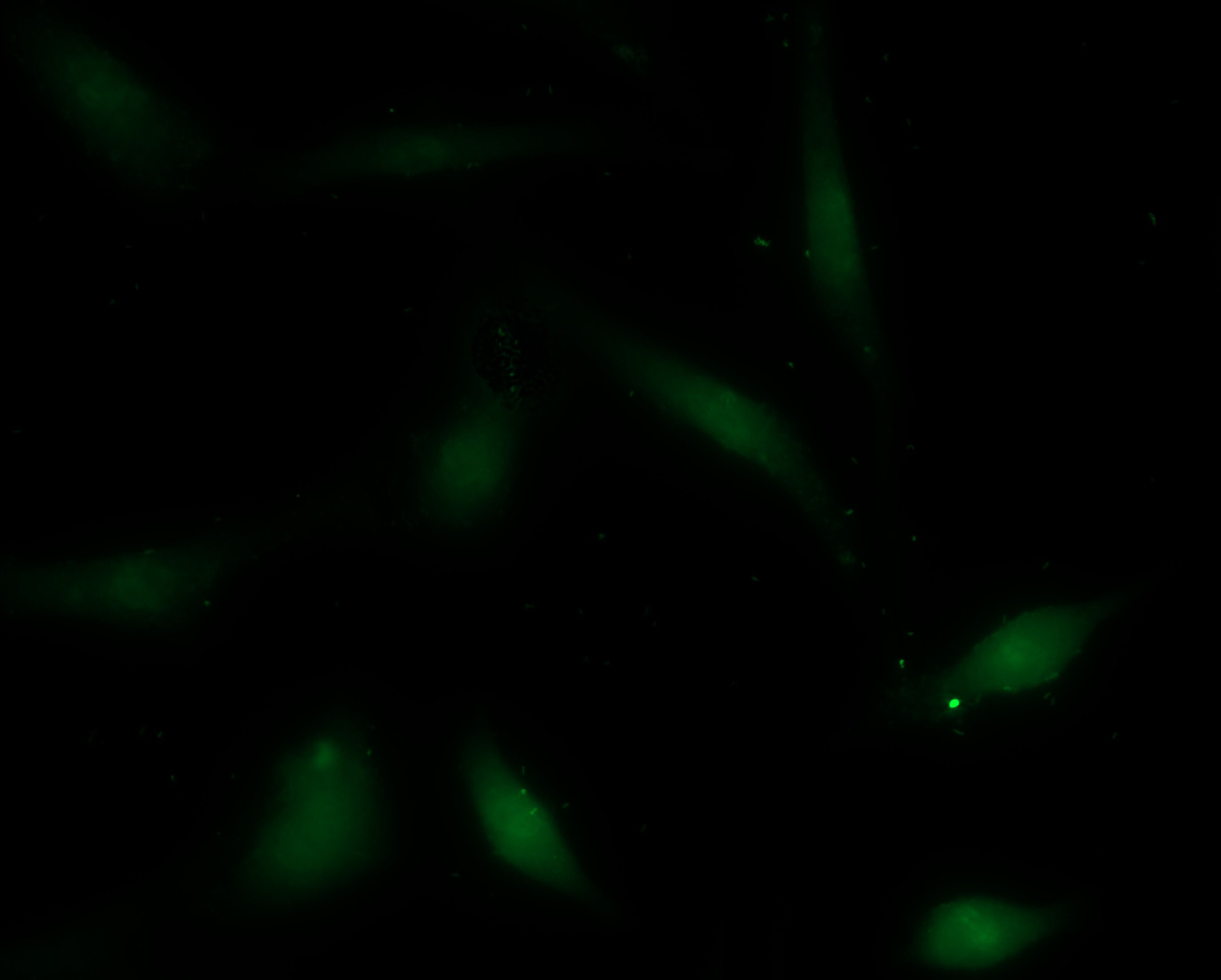

Supplement: S1 File — (ZIP) [file pone.0265049.s003.zip › HDF activation in vitro/DMEM Snap-317_a┴-SMA Alexa Fluor 488.tif]

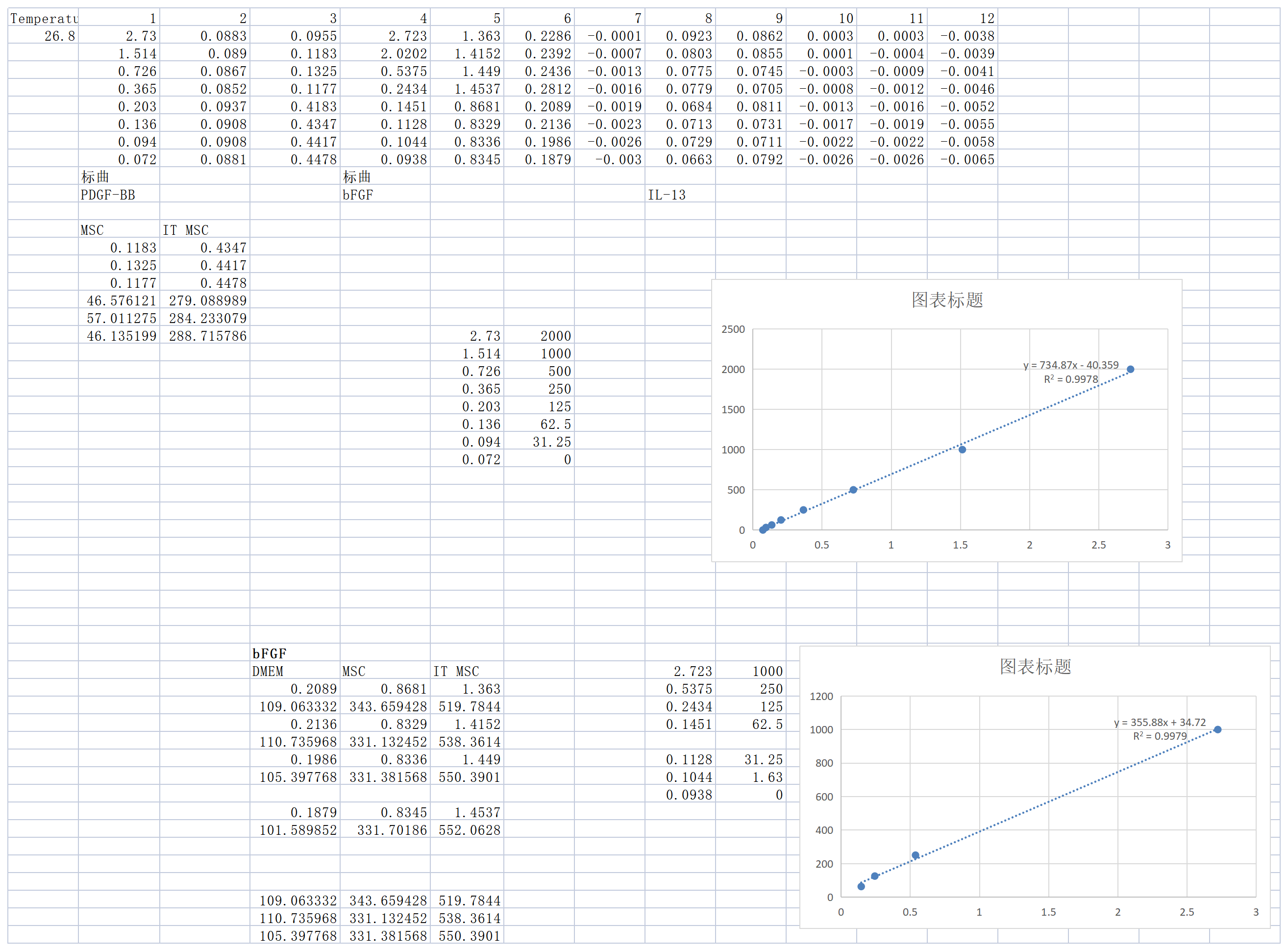

Supplement: S1 File — (ZIP) [file pone.0265049.s003.zip › HDF activation in vitro/ELISA bFGF PDGF-BB.tif]

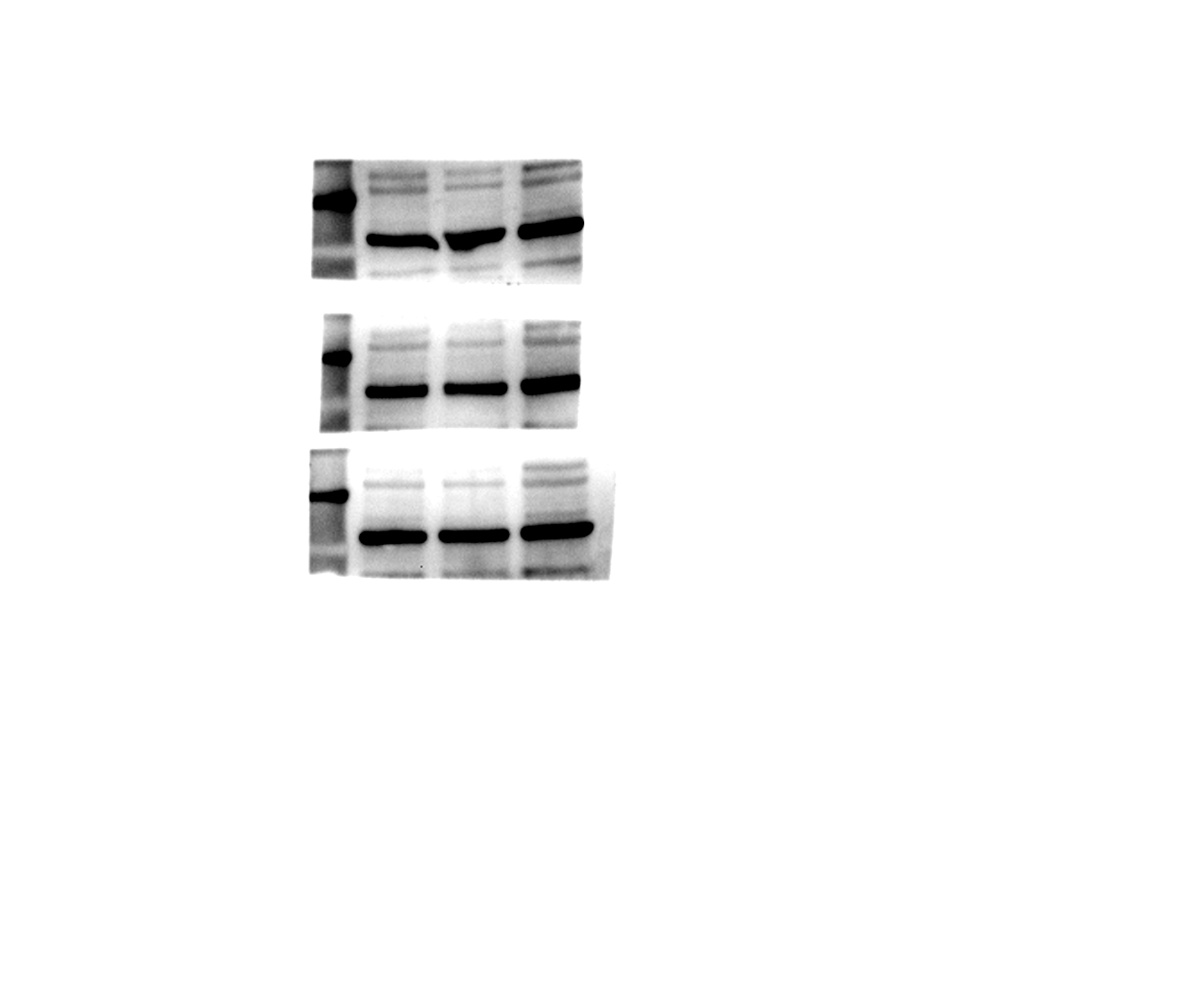

Supplement: S1 File — (ZIP) [file pone.0265049.s003.zip › HDF activation in vitro/HDF ACTIN.Tif]

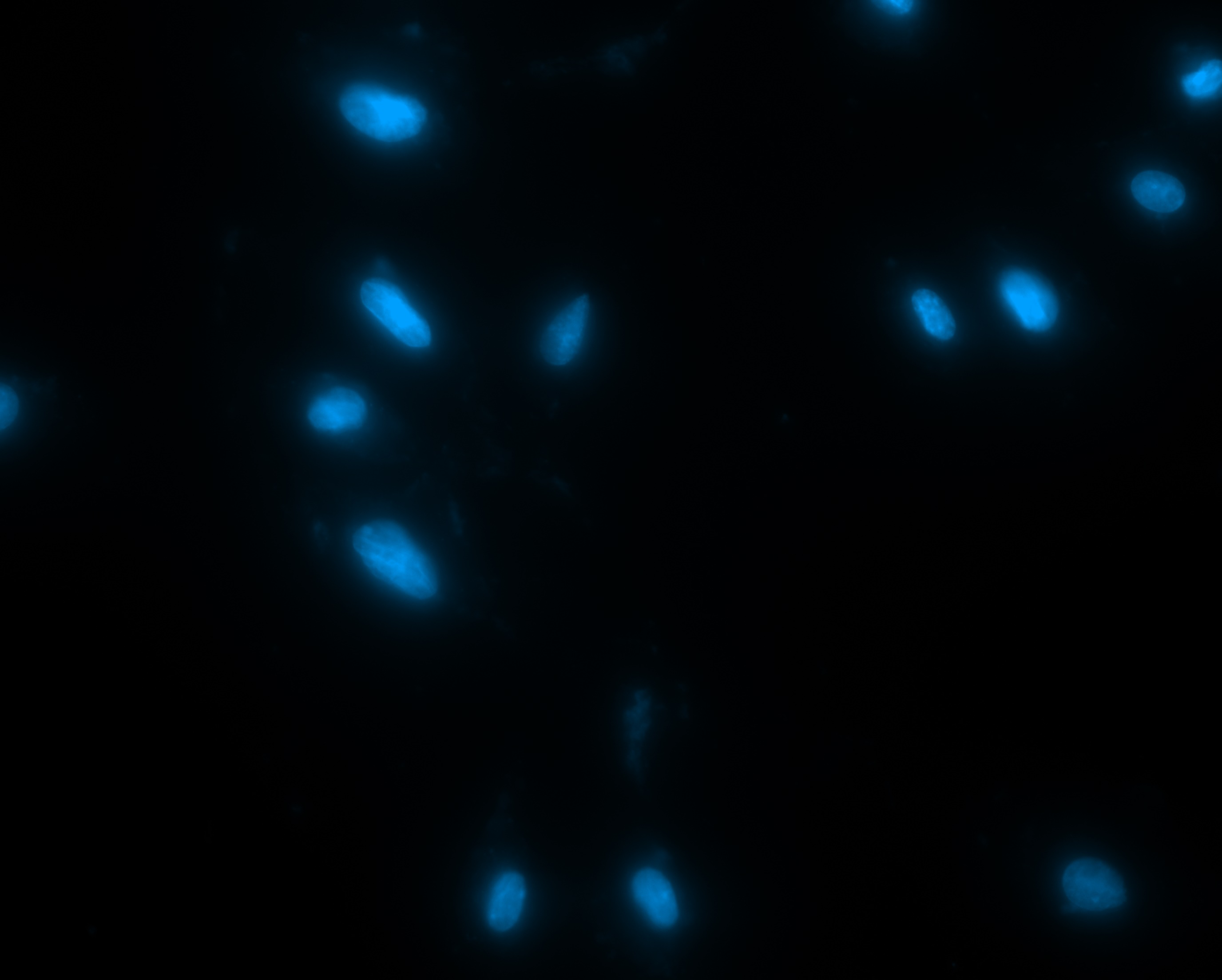

Supplement: S1 File — (ZIP) [file pone.0265049.s003.zip › HDF activation in vitro/IT MSC-CM Snap-358_DAPI.tif]

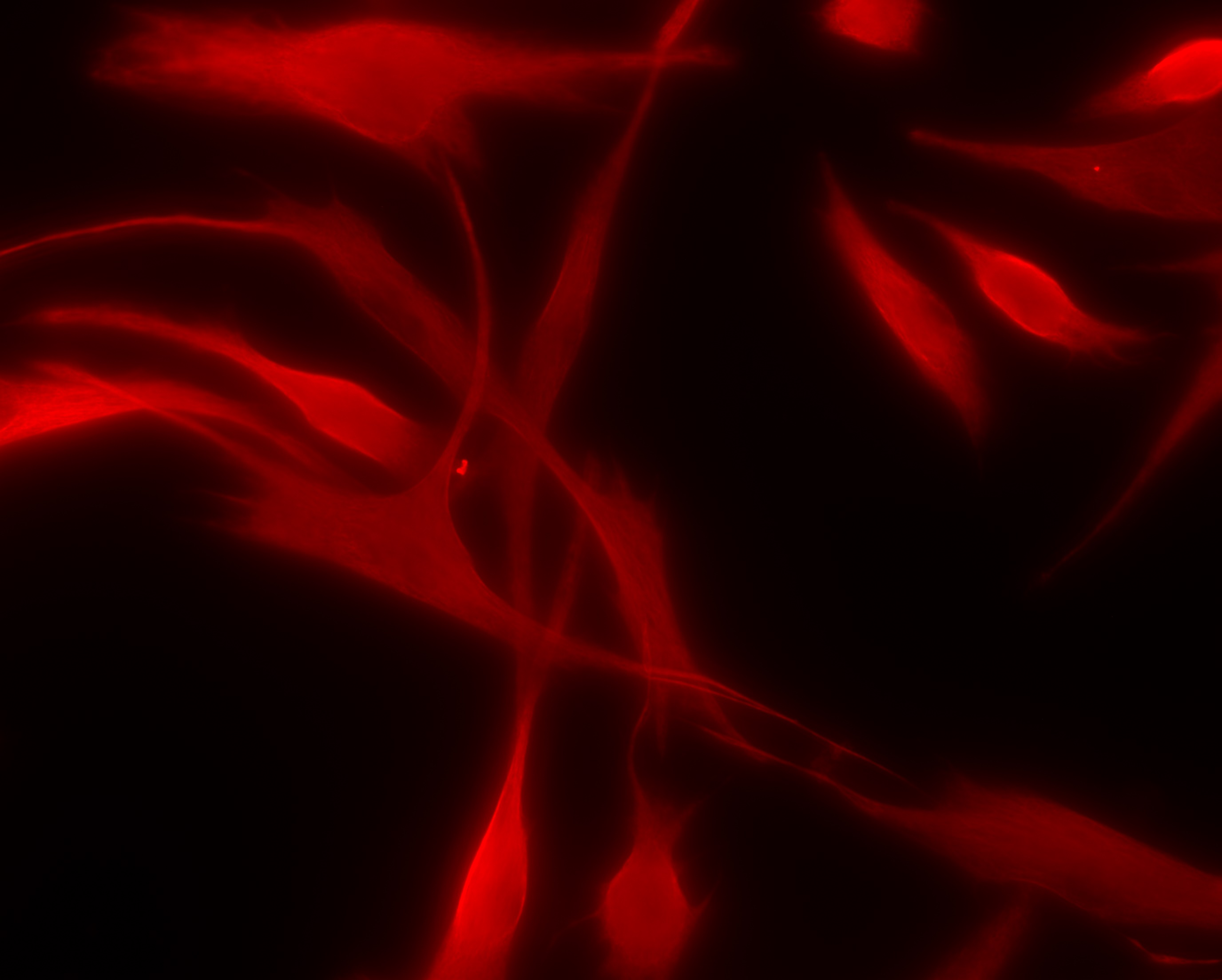

Supplement: S1 File — (ZIP) [file pone.0265049.s003.zip › HDF activation in vitro/IT MSC-CM Snap-358_Vimentin Alexa Fluor 594.tif]

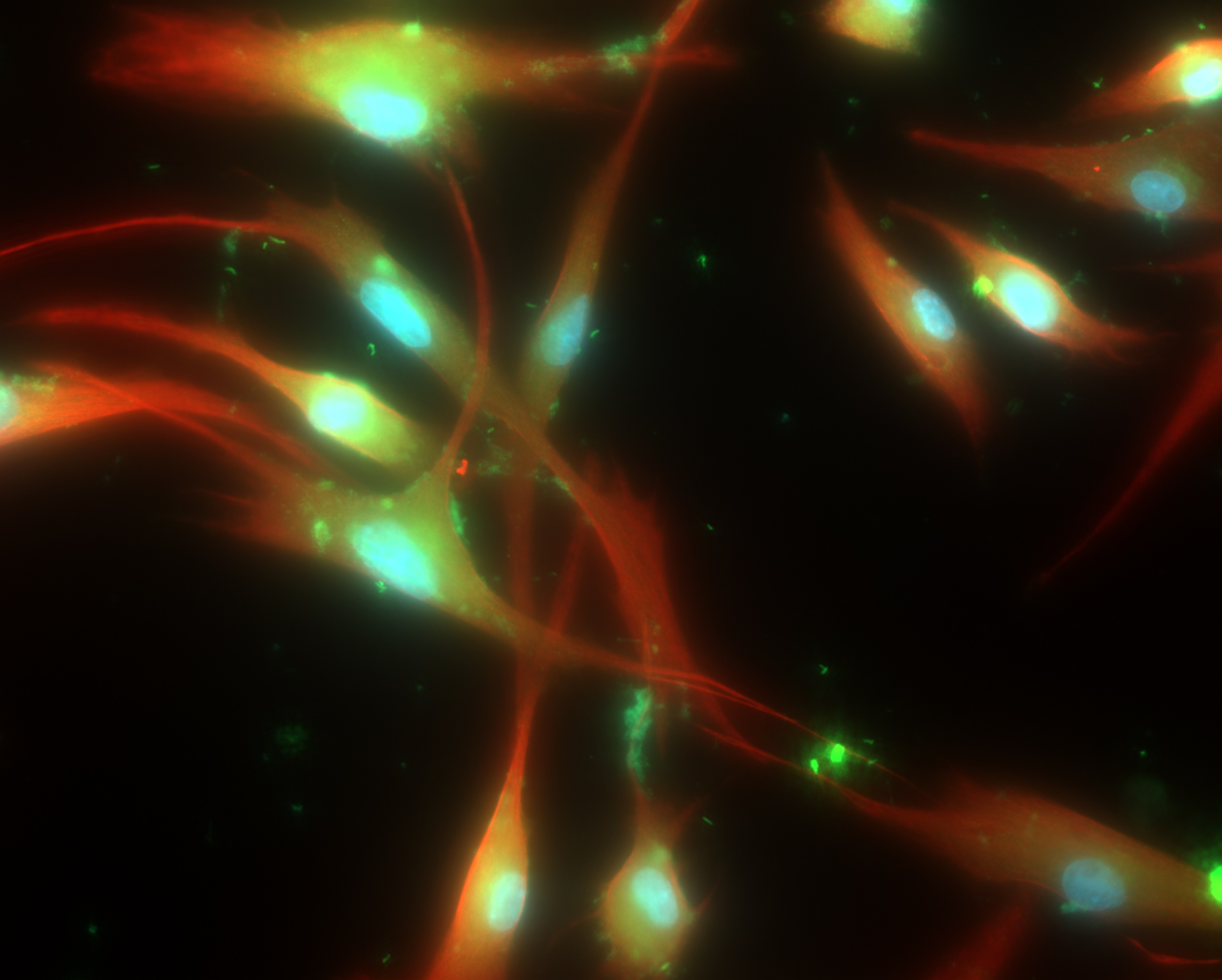

Supplement: S1 File — (ZIP) [file pone.0265049.s003.zip › HDF activation in vitro/IT MSC-CM Snap-358_merge.tif]

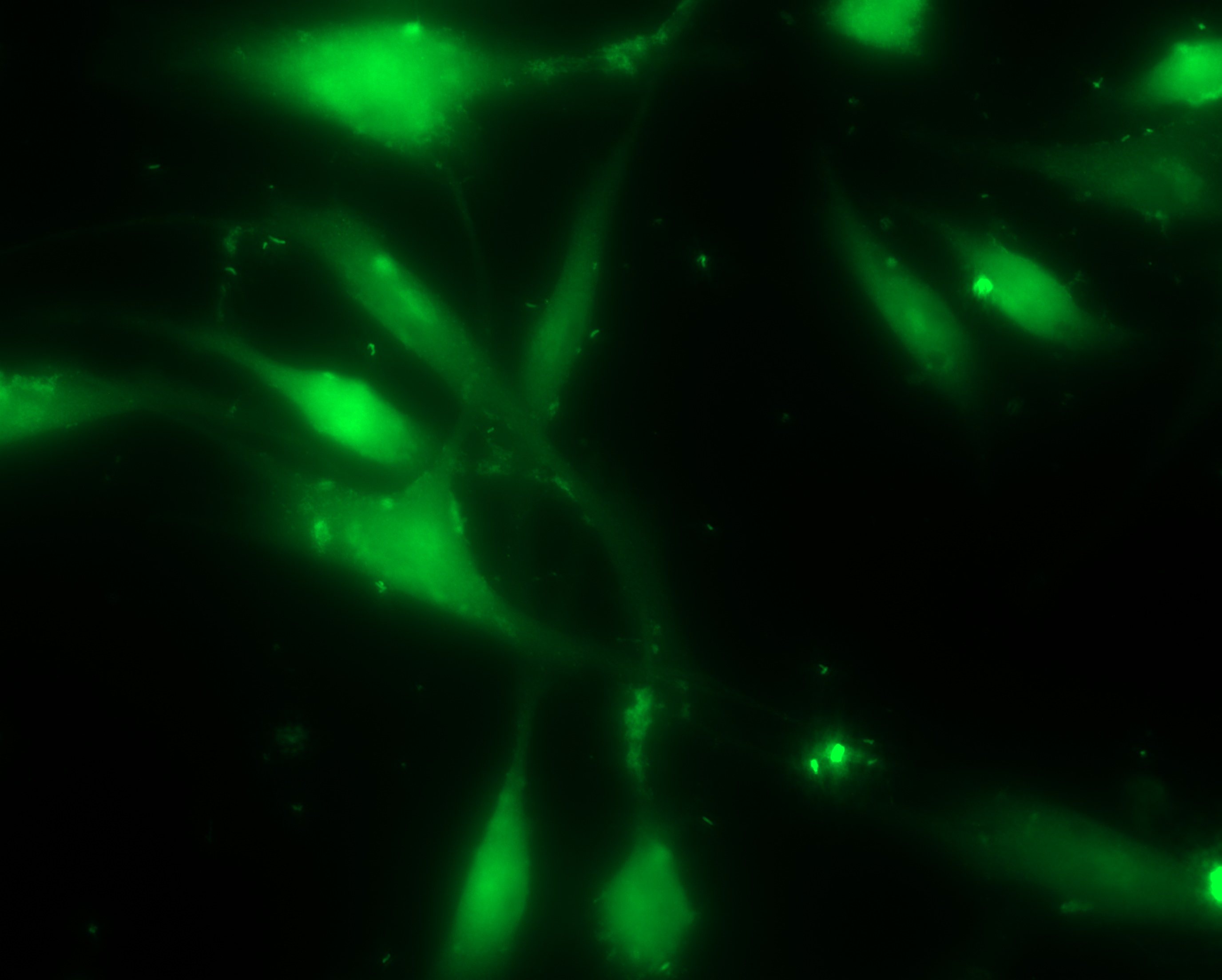

Supplement: S1 File — (ZIP) [file pone.0265049.s003.zip › HDF activation in vitro/IT MSC-CM Snap-358_a┴-SMA Alexa Fluor 488.tif]

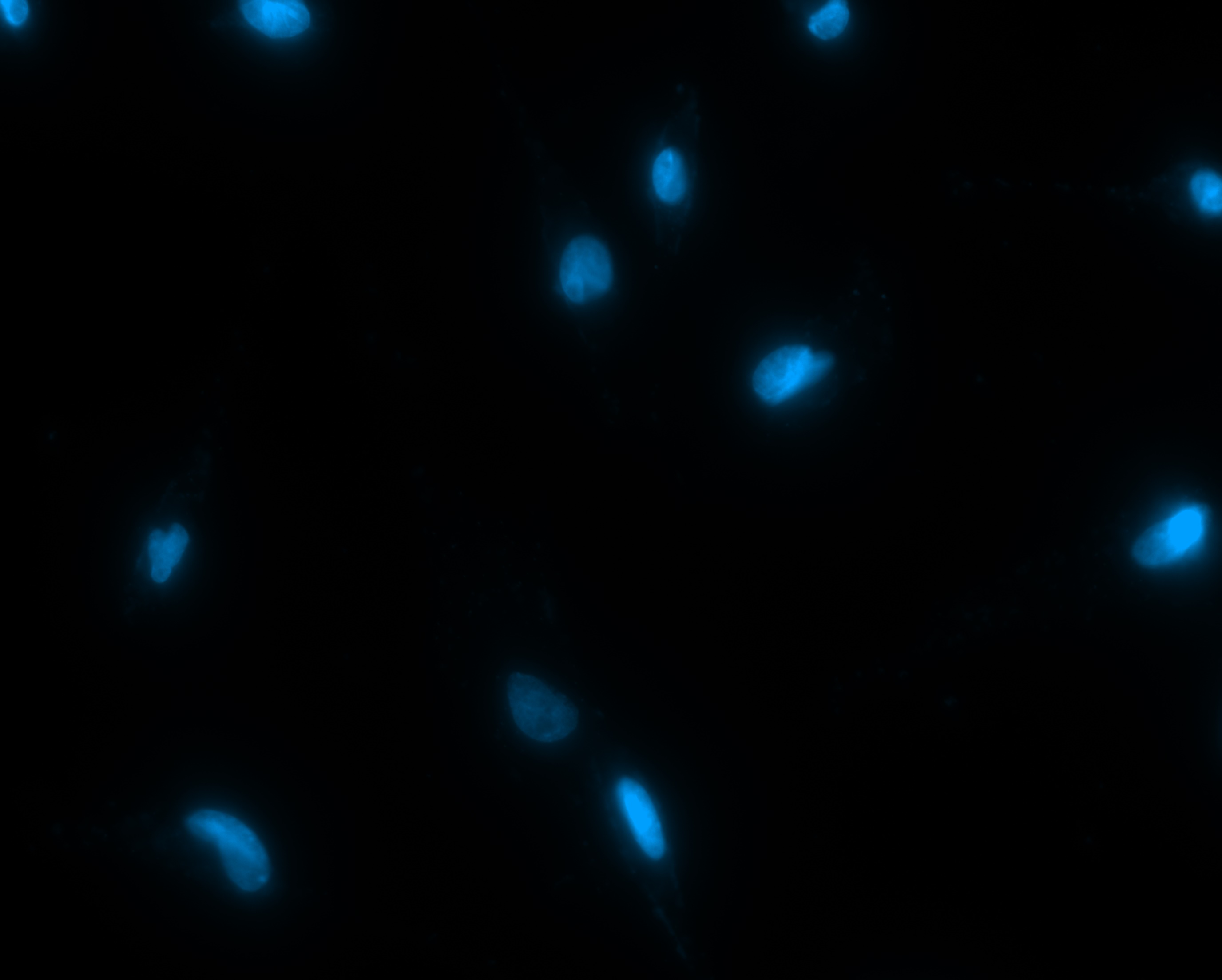

Supplement: S1 File — (ZIP) [file pone.0265049.s003.zip › HDF activation in vitro/MSC-CM Snap-340_DAPI.tif]

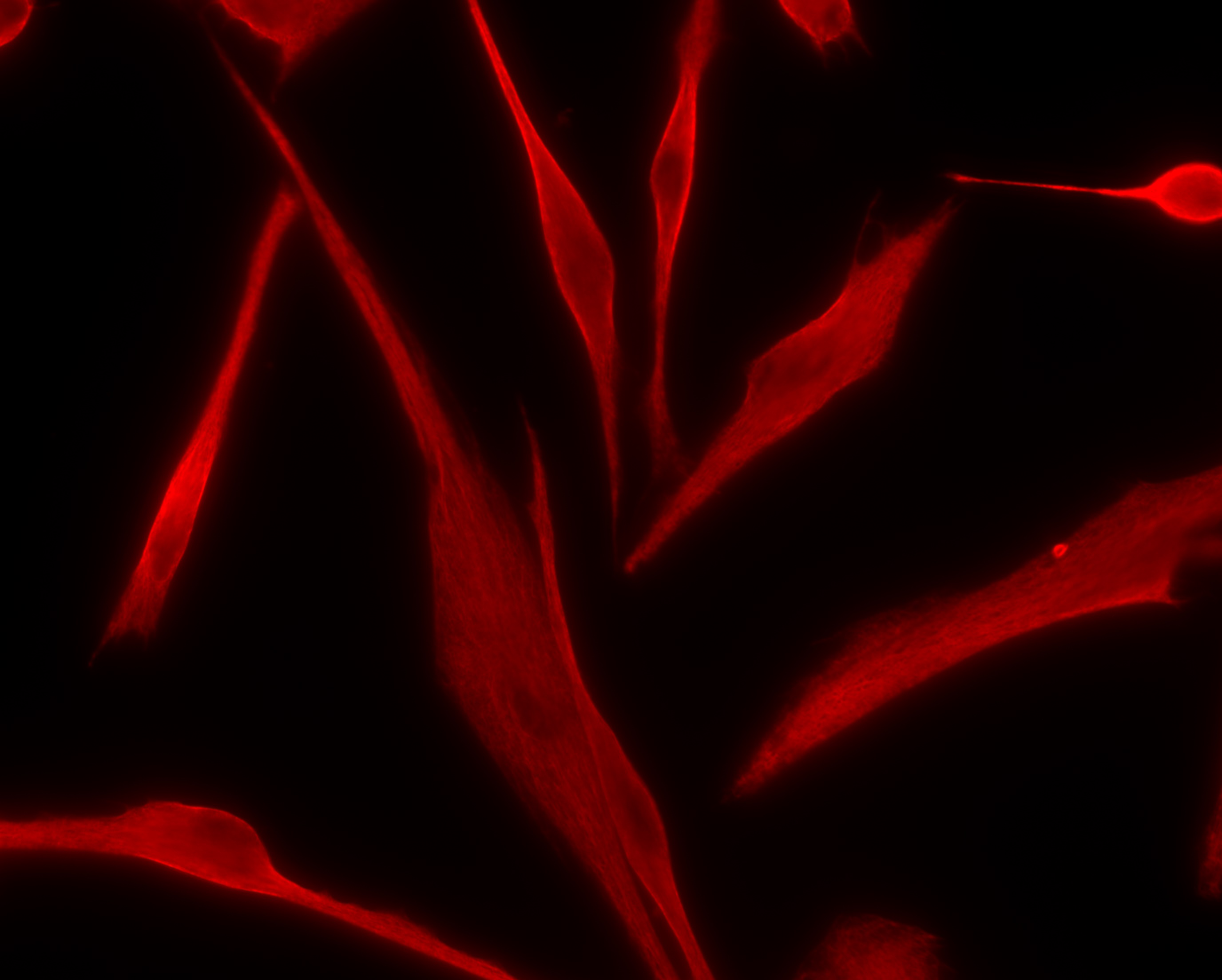

Supplement: S1 File — (ZIP) [file pone.0265049.s003.zip › HDF activation in vitro/MSC-CM Snap-340_Vimentin Alexa Fluor 594.tif]

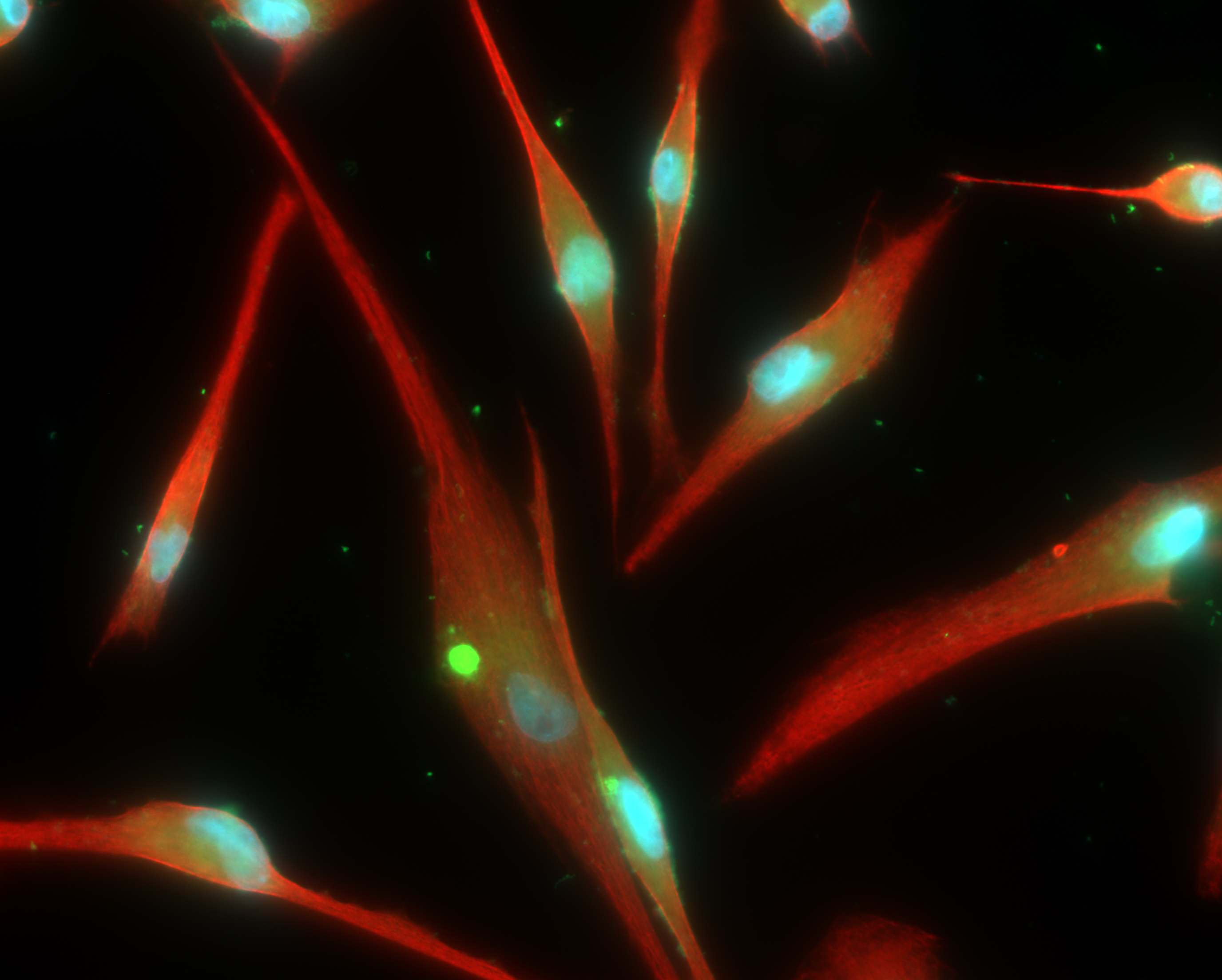

Supplement: S1 File — (ZIP) [file pone.0265049.s003.zip › HDF activation in vitro/MSC-CM Snap-340_merge.tif]

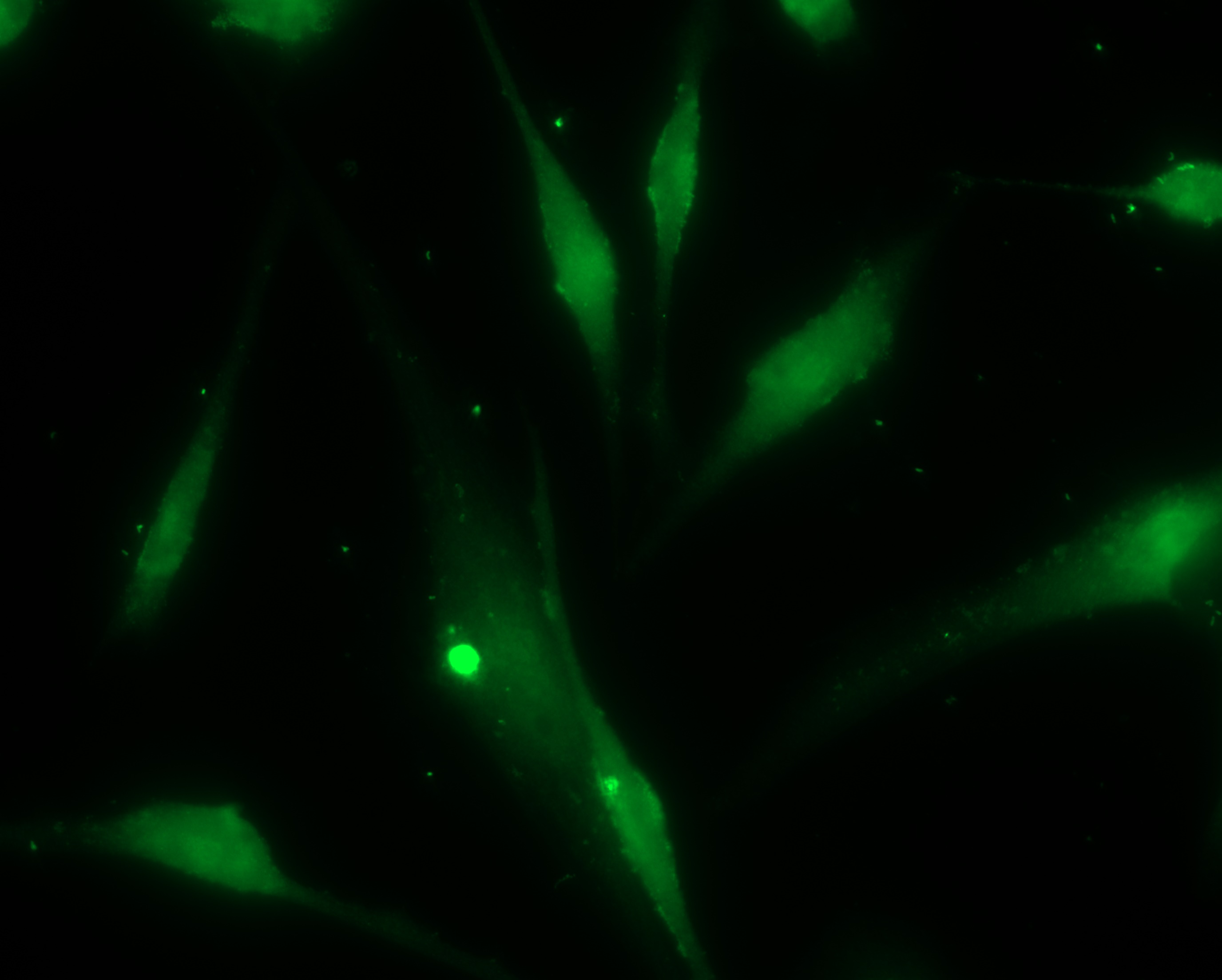

Supplement: S1 File — (ZIP) [file pone.0265049.s003.zip › HDF activation in vitro/MSC-CM Snap-340_a┴-SMA Alexa Fluor 488.tif]

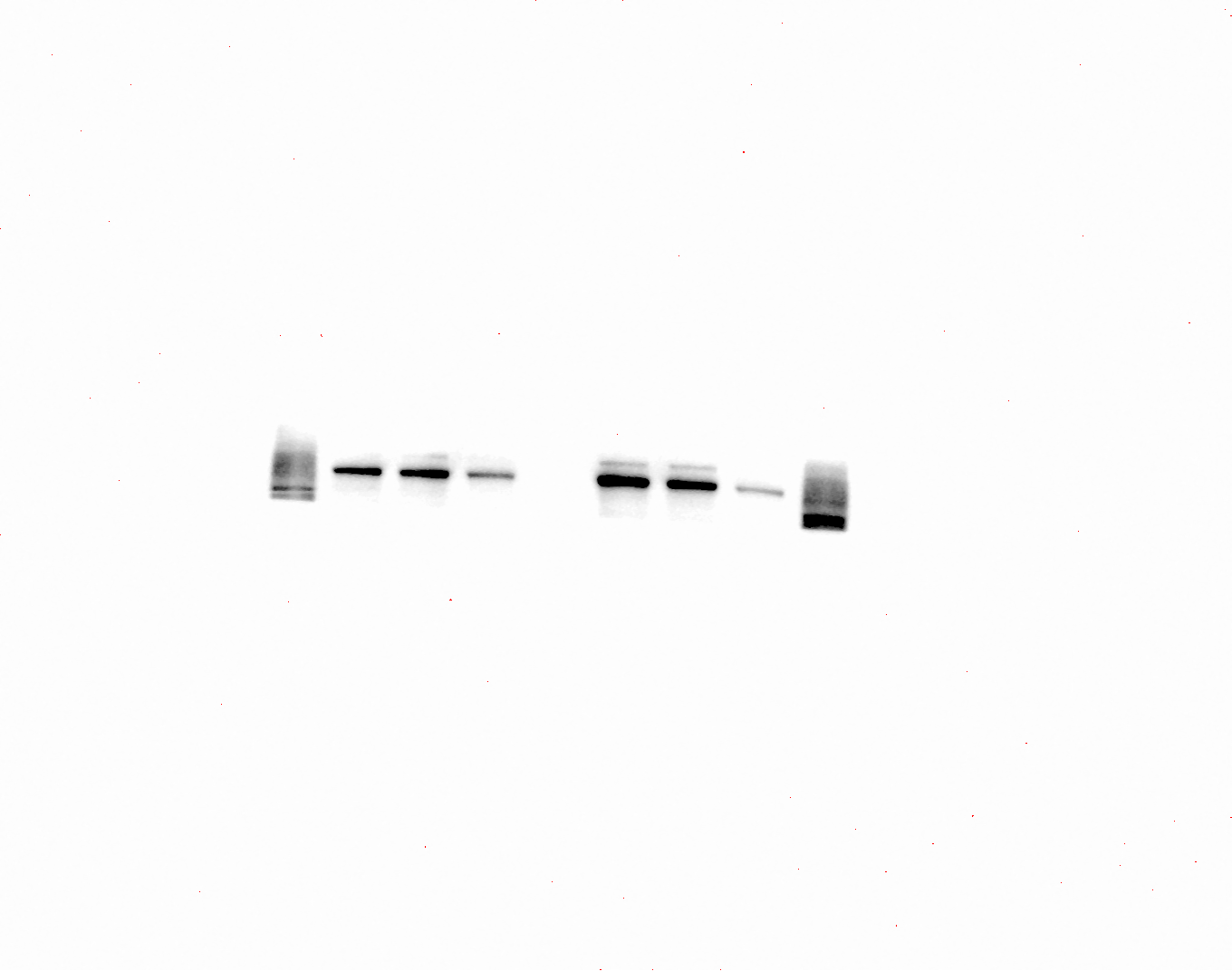

Supplement: S1 File — (ZIP) [file pone.0265049.s003.zip › HDF activation in vitro/collagen ó±.tif]

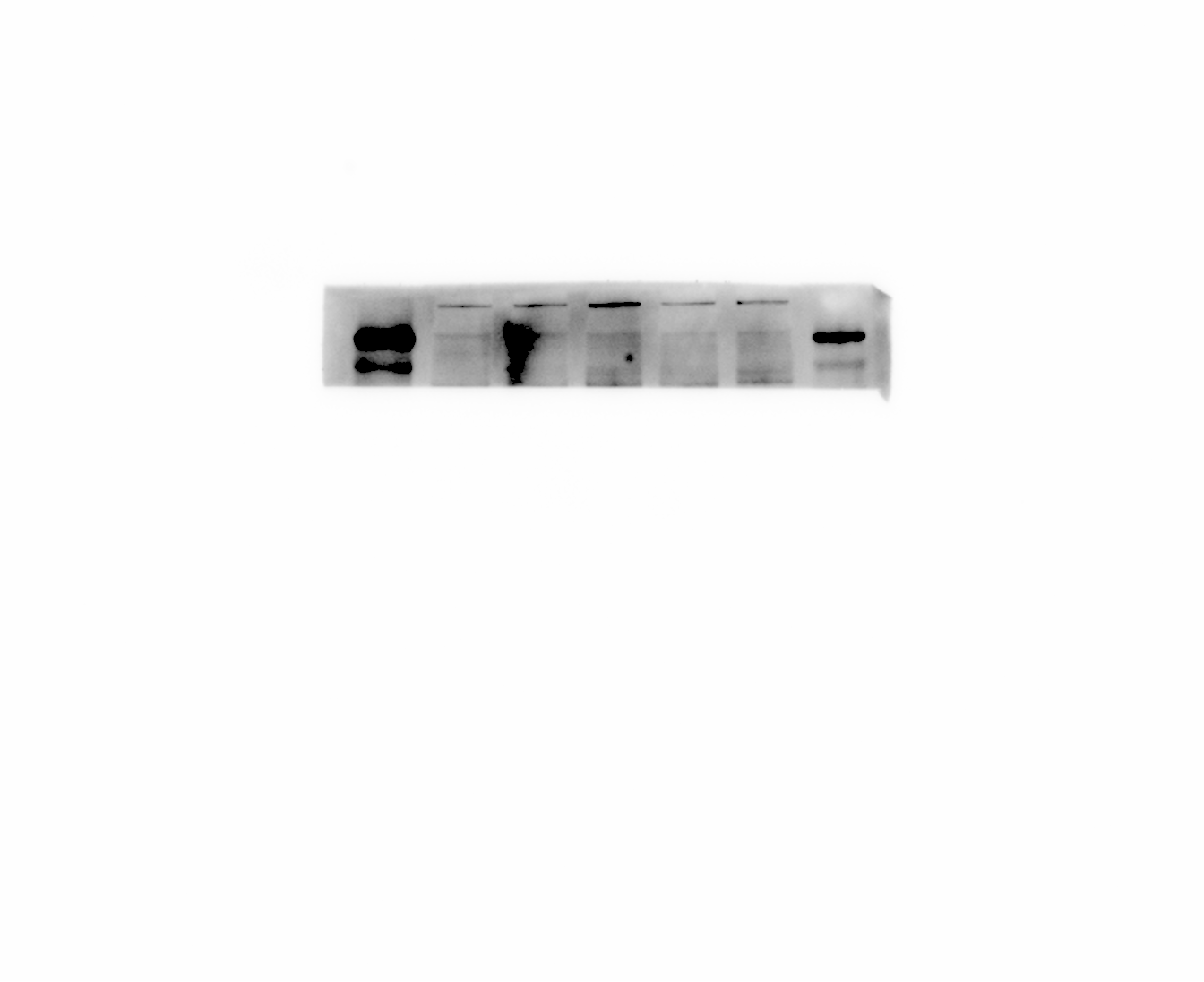

Supplement: S1 File — (ZIP) [file pone.0265049.s003.zip › HDF activation in vitro/collagen ó≤.tif]

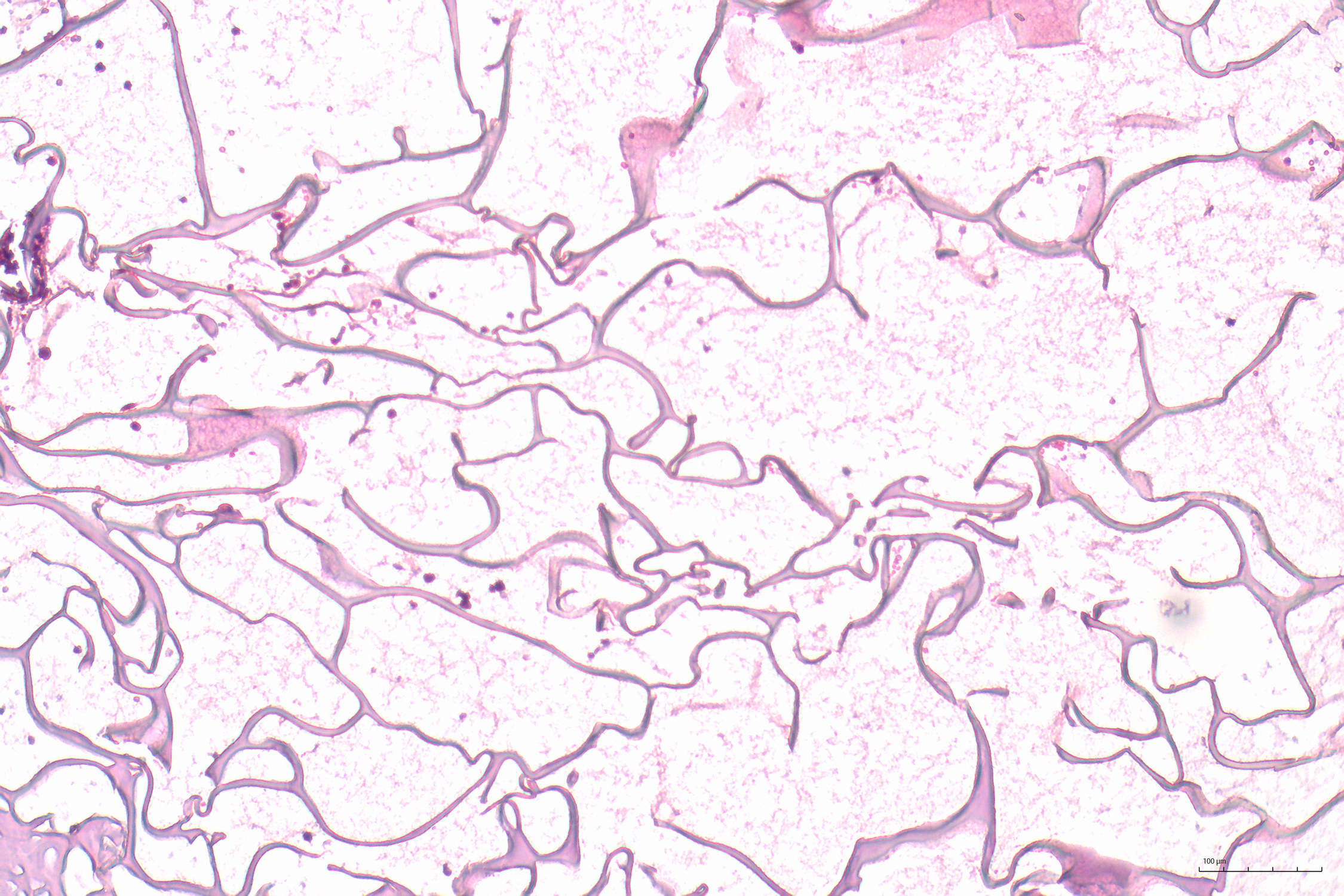

Supplement: S2 File — (ZIP) [file pone.0265049.s004.zip › HE/SF 3D.tif]

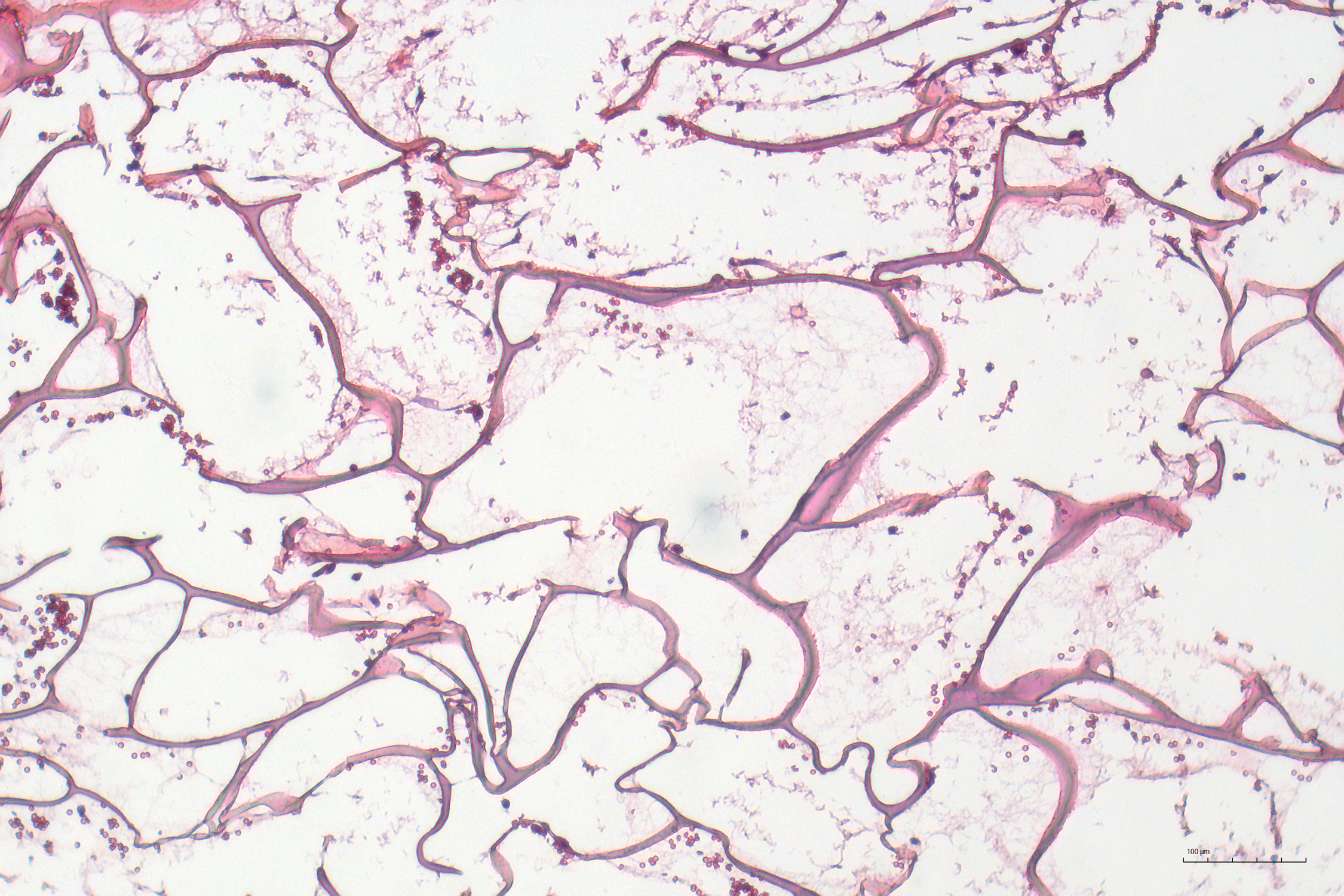

Supplement: S2 File — (ZIP) [file pone.0265049.s004.zip › HE/SF 7D.tif]

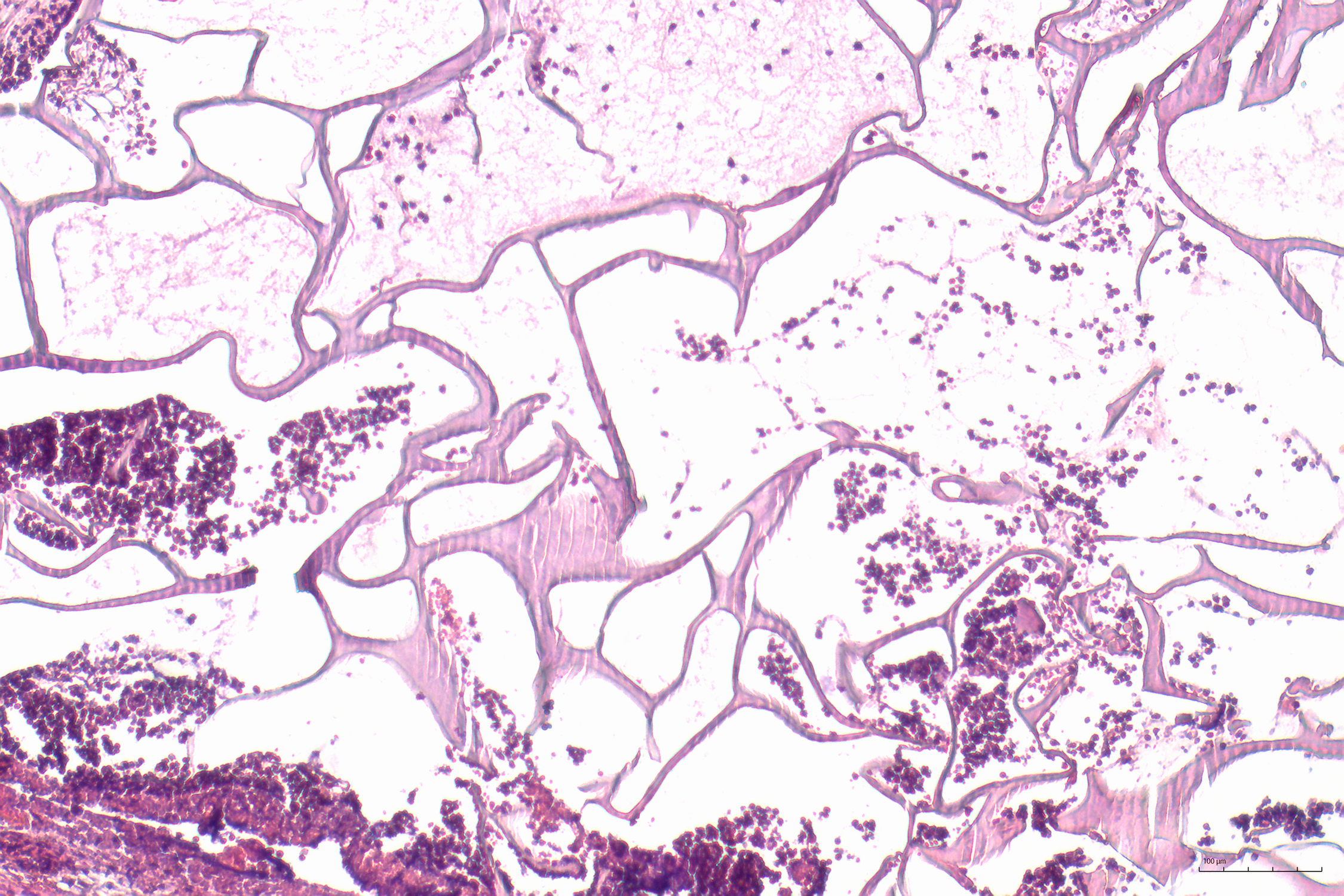

Supplement: S2 File — (ZIP) [file pone.0265049.s004.zip › HE/SF+LN+IT MSC-CM 3D.tif]

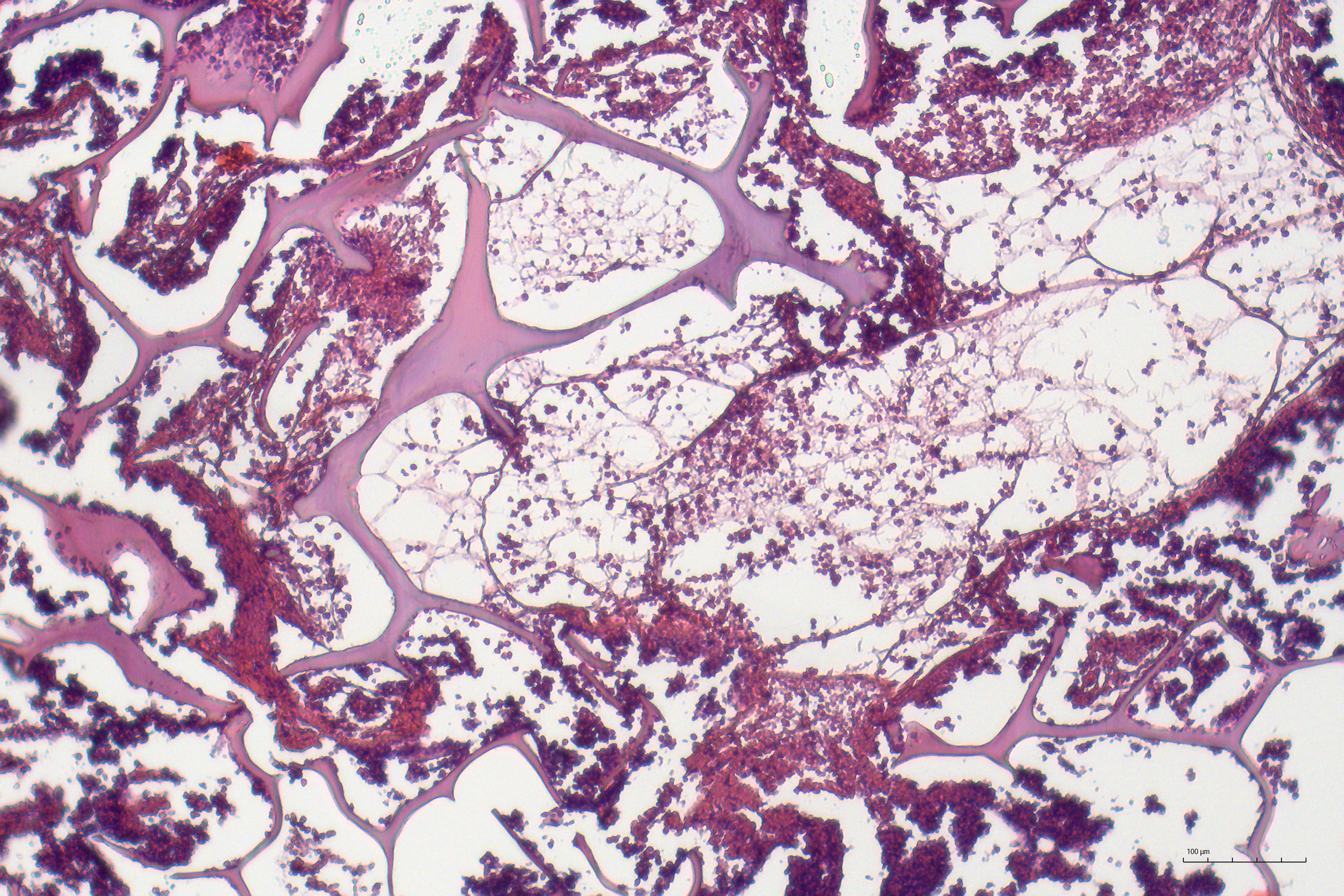

Supplement: S2 File — (ZIP) [file pone.0265049.s004.zip › HE/SF+LN+IT MSC-CM 7D.tif]

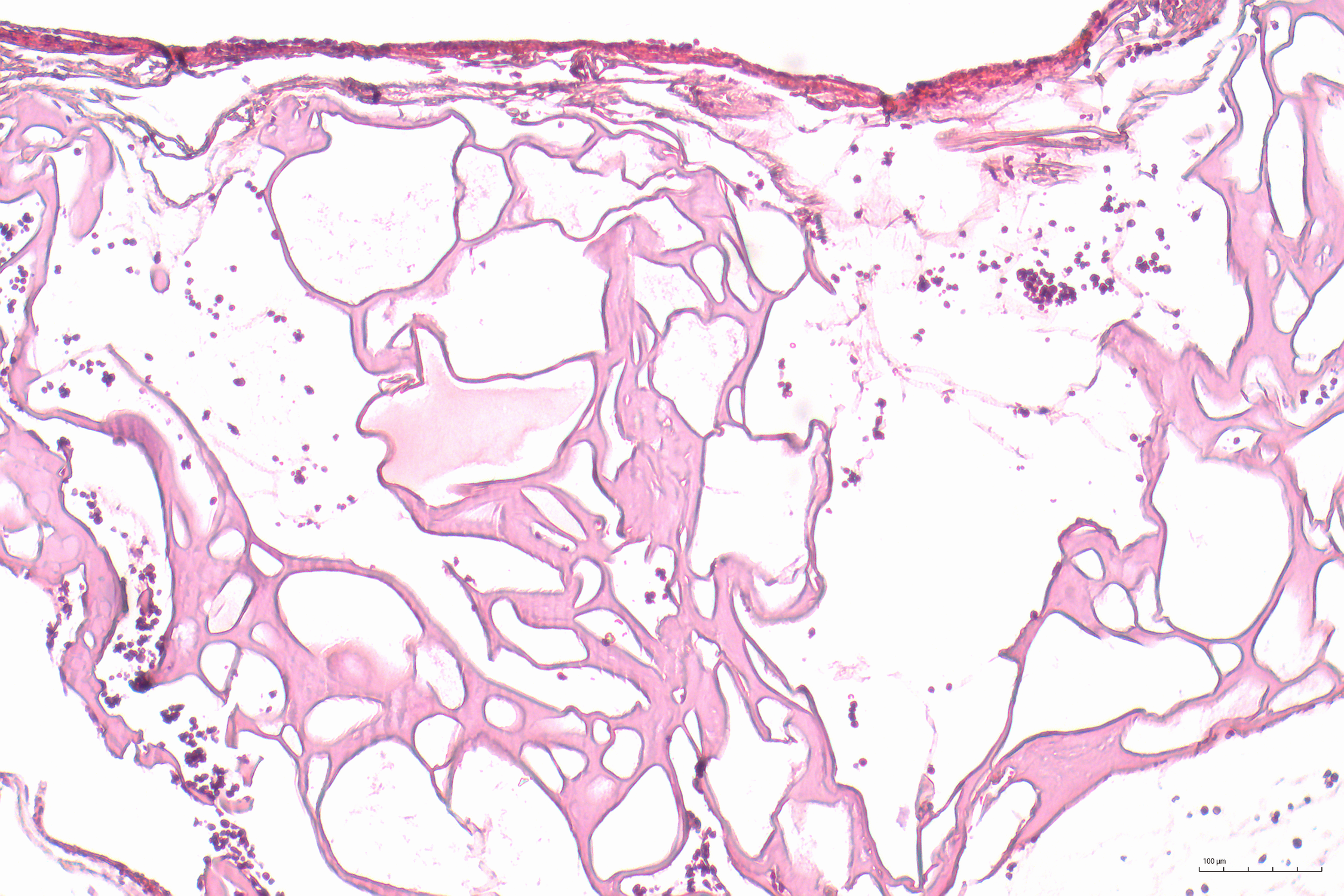

Supplement: S2 File — (ZIP) [file pone.0265049.s004.zip › HE/SF+LN+MSC-CM 3D.tif]

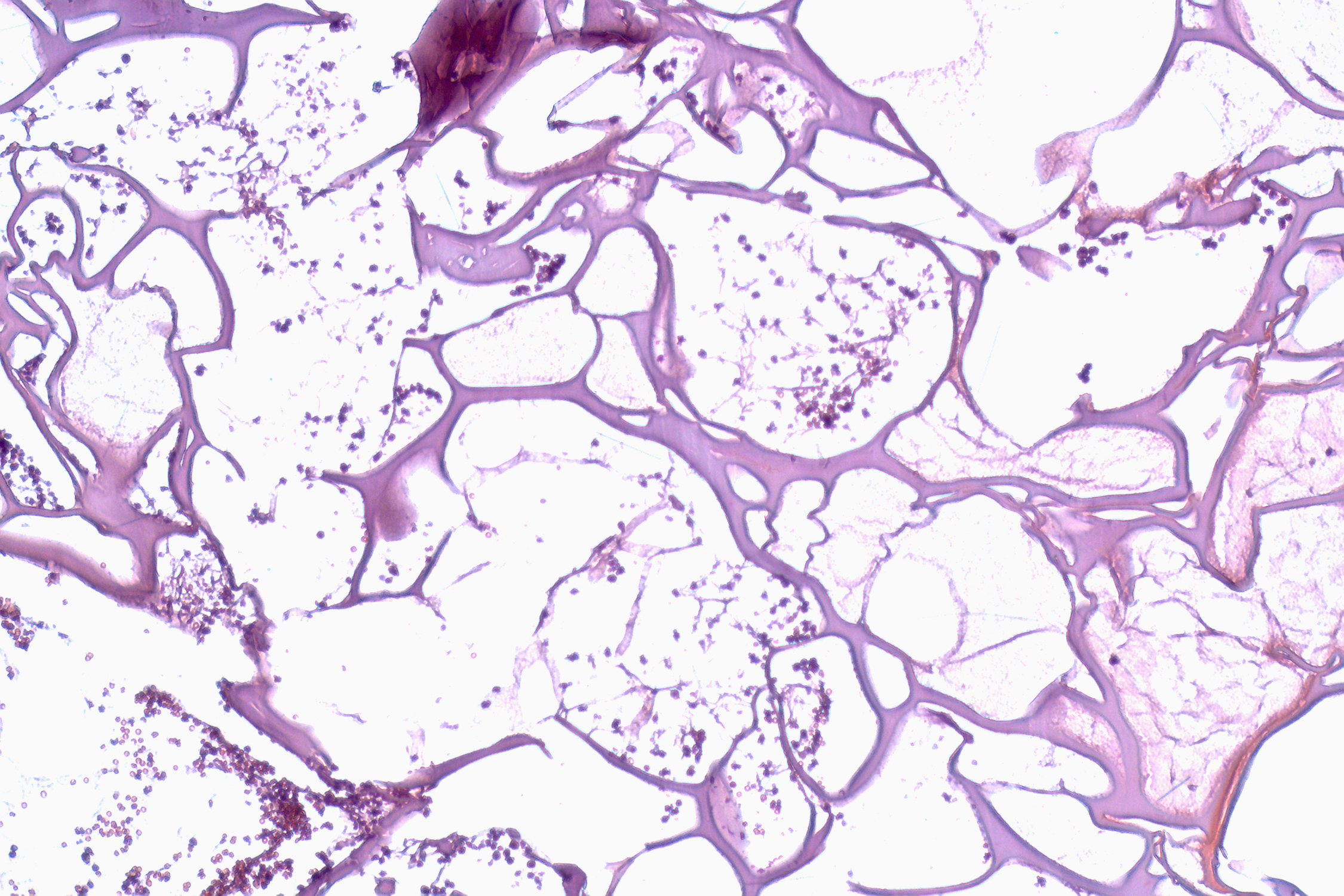

Supplement: S2 File — (ZIP) [file pone.0265049.s004.zip › HE/SF+LN+MSC-CM 7D.tif]

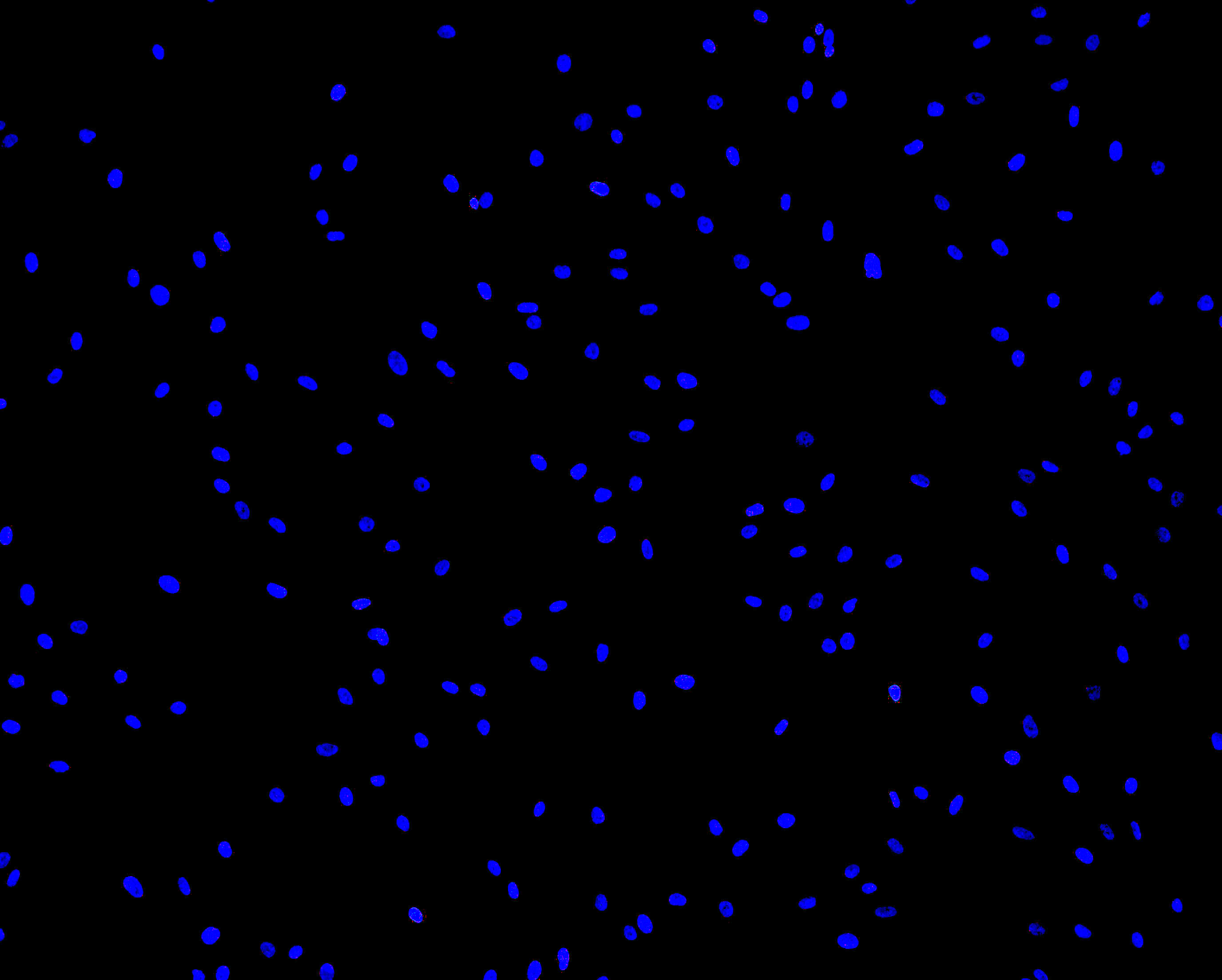

Supplement: S3 File — (ZIP) [file pone.0265049.s005.zip › Identification of UC-MSC and HDF/HDF DAPI.tif]

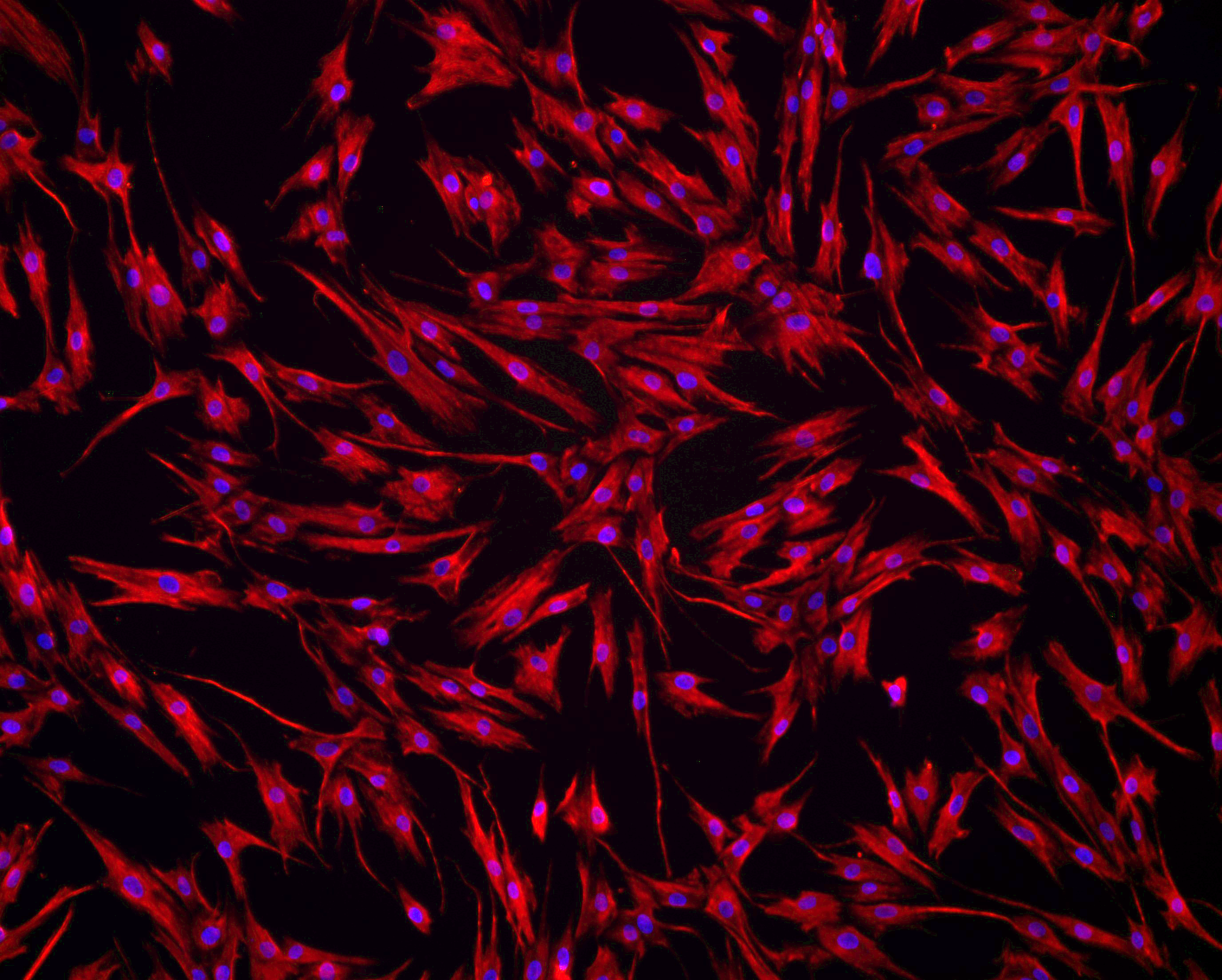

Supplement: S3 File — (ZIP) [file pone.0265049.s005.zip › Identification of UC-MSC and HDF/HDF Merged.tif]

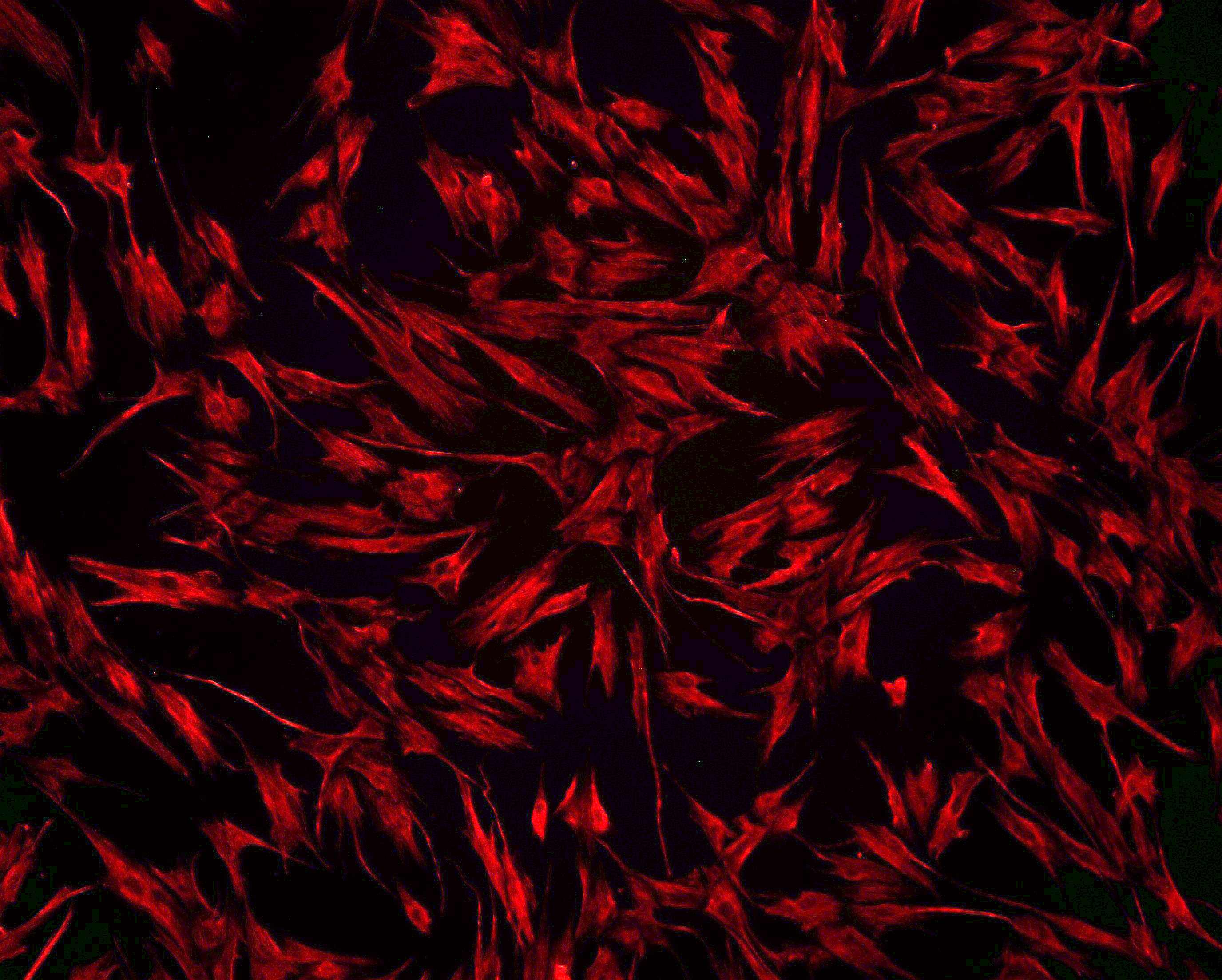

Supplement: S3 File — (ZIP) [file pone.0265049.s005.zip › Identification of UC-MSC and HDF/HDF Vimentin.tif]

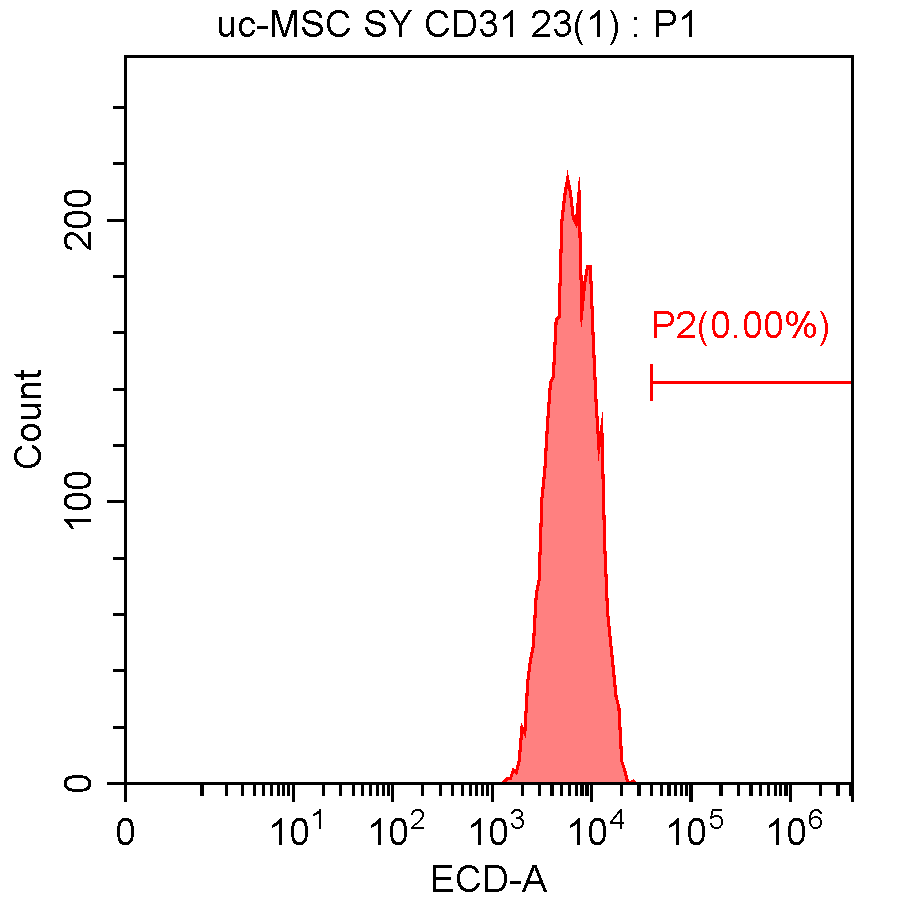

Supplement: S3 File — (ZIP) [file pone.0265049.s005.zip › Identification of UC-MSC and HDF/UC-MSC CD31.tif]

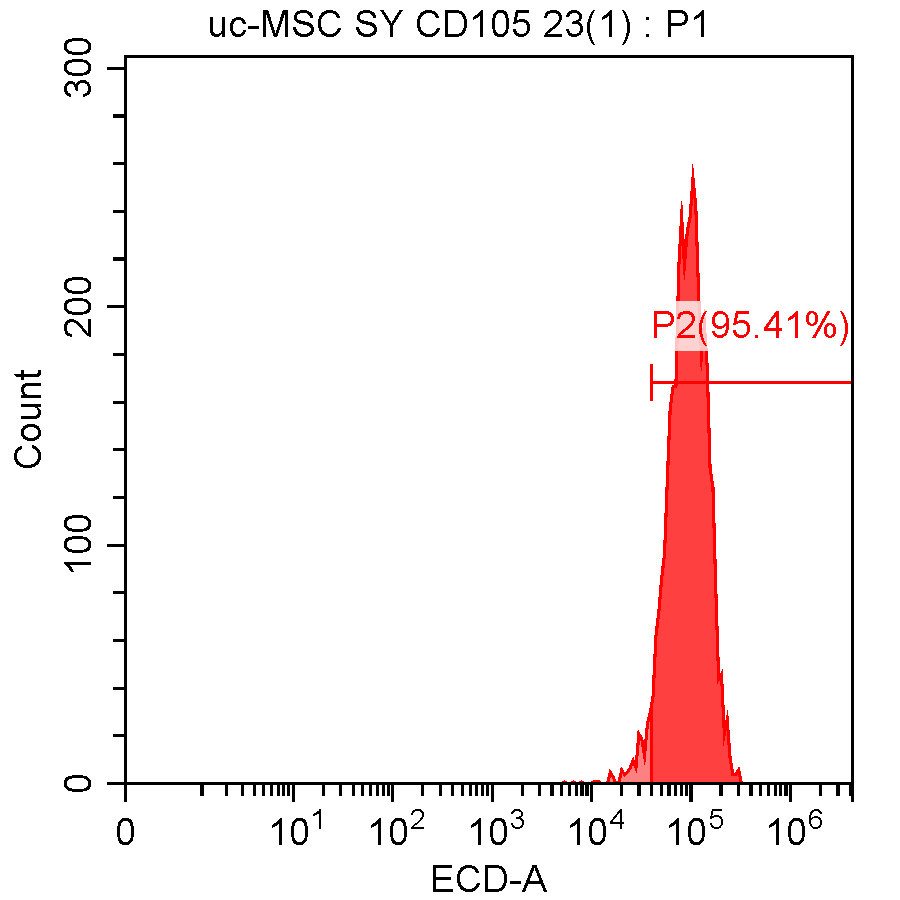

Supplement: S3 File — (ZIP) [file pone.0265049.s005.zip › Identification of UC-MSC and HDF/UC-MSC CD105.tif]

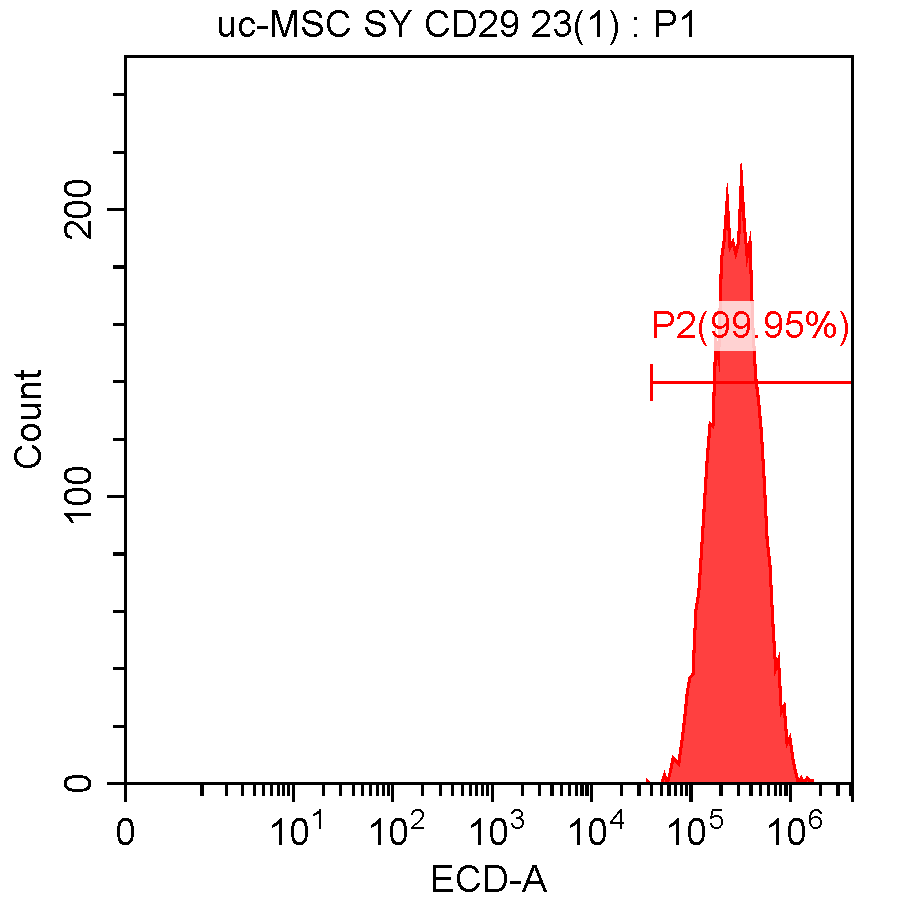

Supplement: S3 File — (ZIP) [file pone.0265049.s005.zip › Identification of UC-MSC and HDF/UC-MSC CD29.tif]

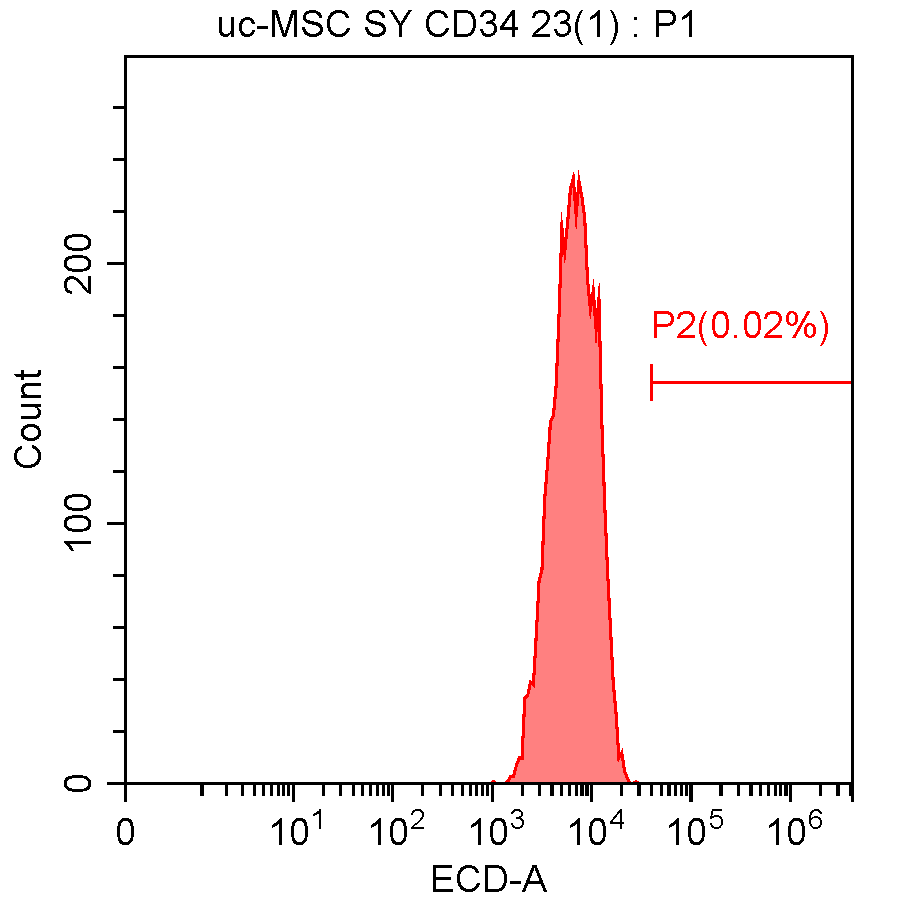

Supplement: S3 File — (ZIP) [file pone.0265049.s005.zip › Identification of UC-MSC and HDF/UC-MSC CD34.tif]

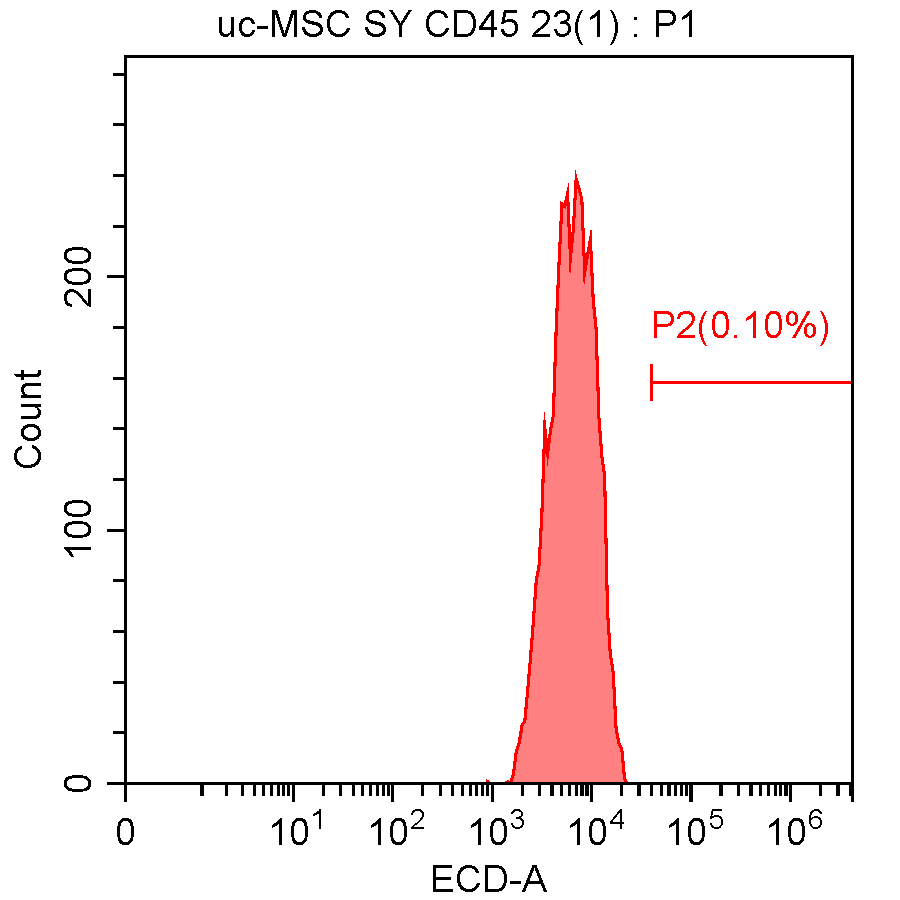

Supplement: S3 File — (ZIP) [file pone.0265049.s005.zip › Identification of UC-MSC and HDF/UC-MSC CD45.tif]

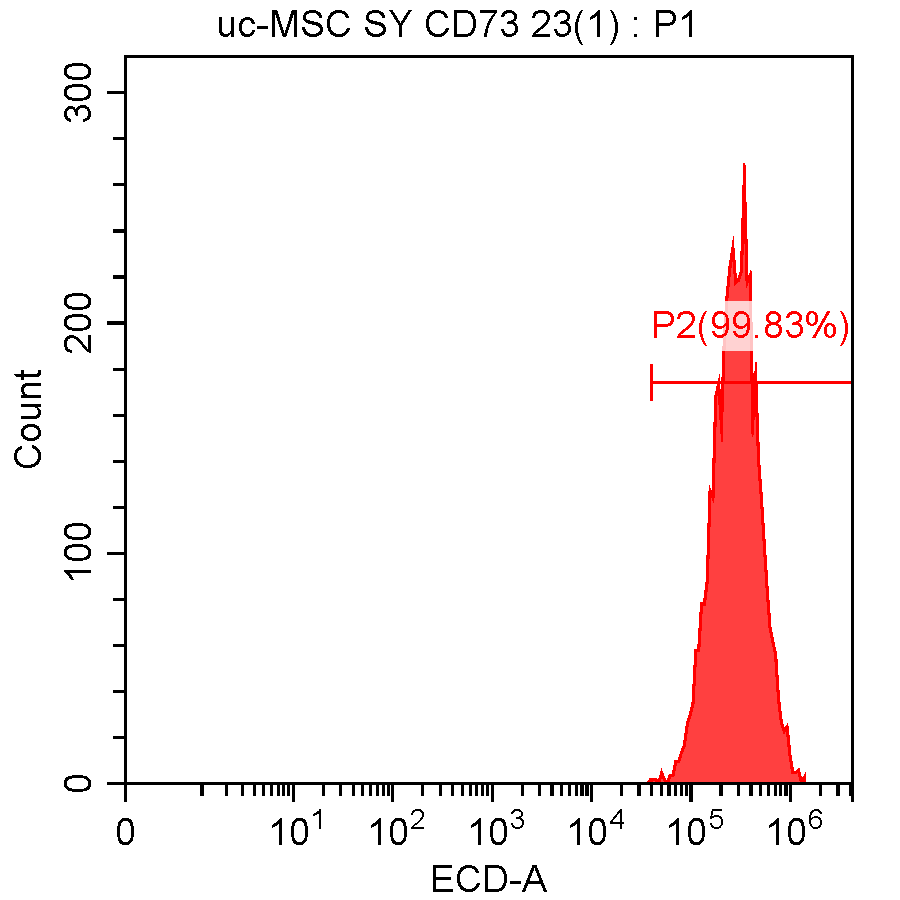

Supplement: S3 File — (ZIP) [file pone.0265049.s005.zip › Identification of UC-MSC and HDF/UC-MSC CD73.tif]

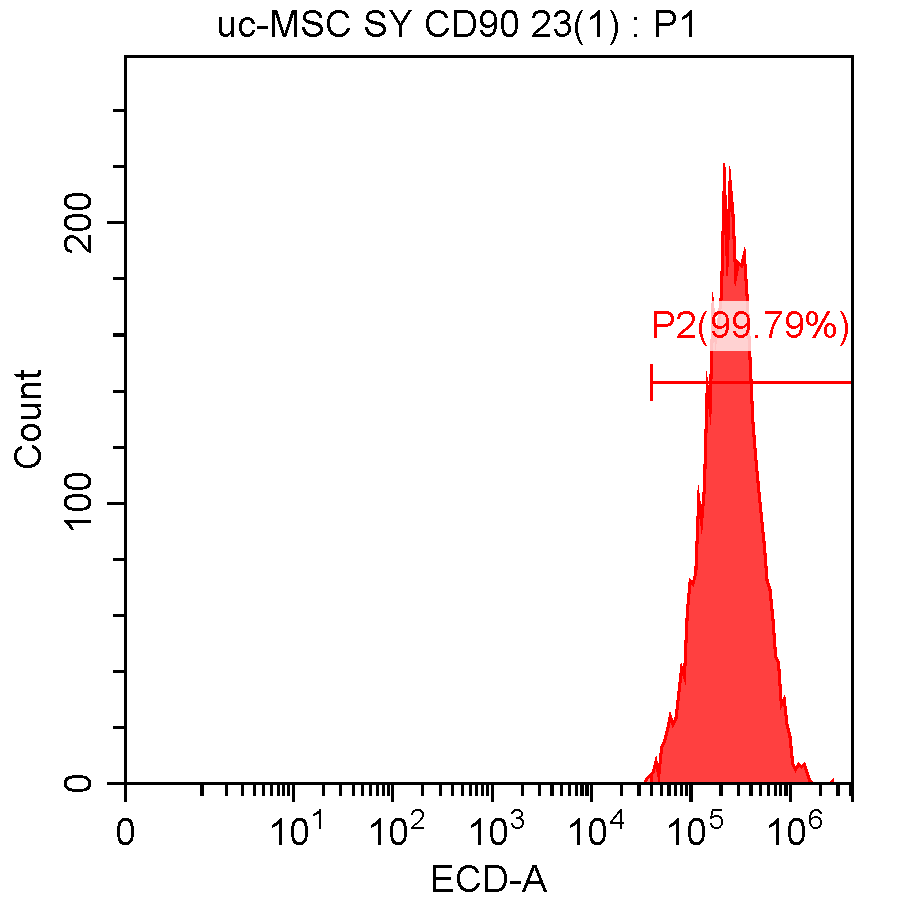

Supplement: S3 File — (ZIP) [file pone.0265049.s005.zip › Identification of UC-MSC and HDF/UC-MSC CD90.tif]

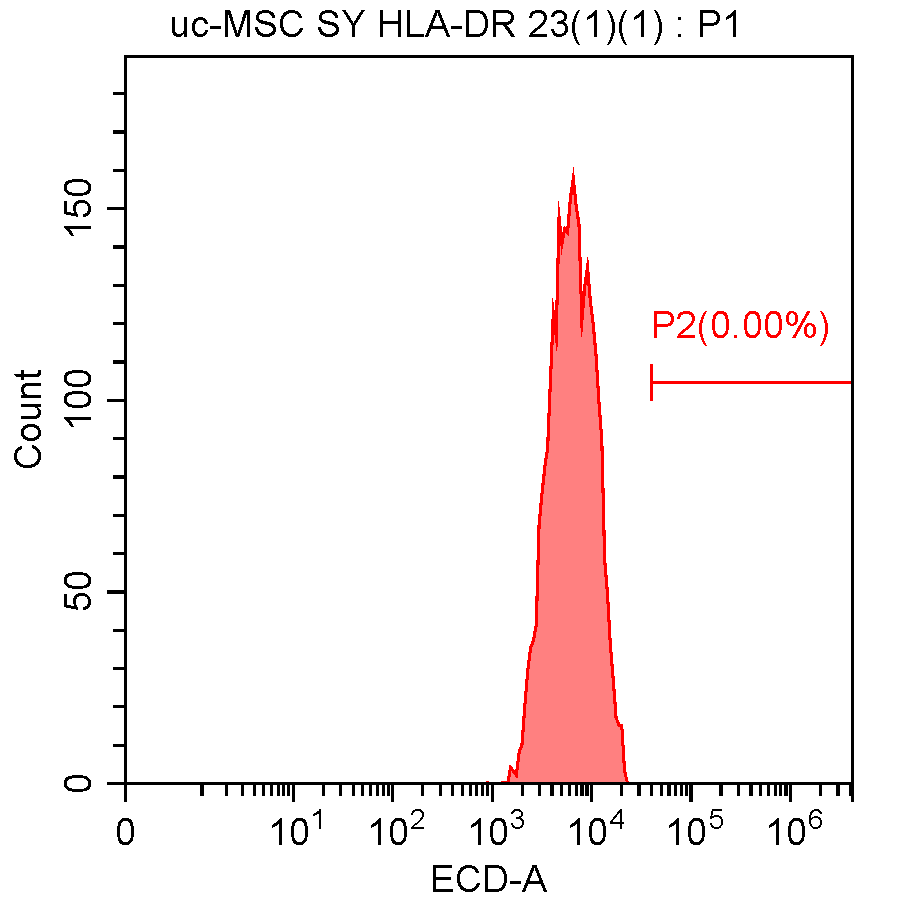

Supplement: S3 File — (ZIP) [file pone.0265049.s005.zip › Identification of UC-MSC and HDF/UC-MSC HLA-DR.tif]

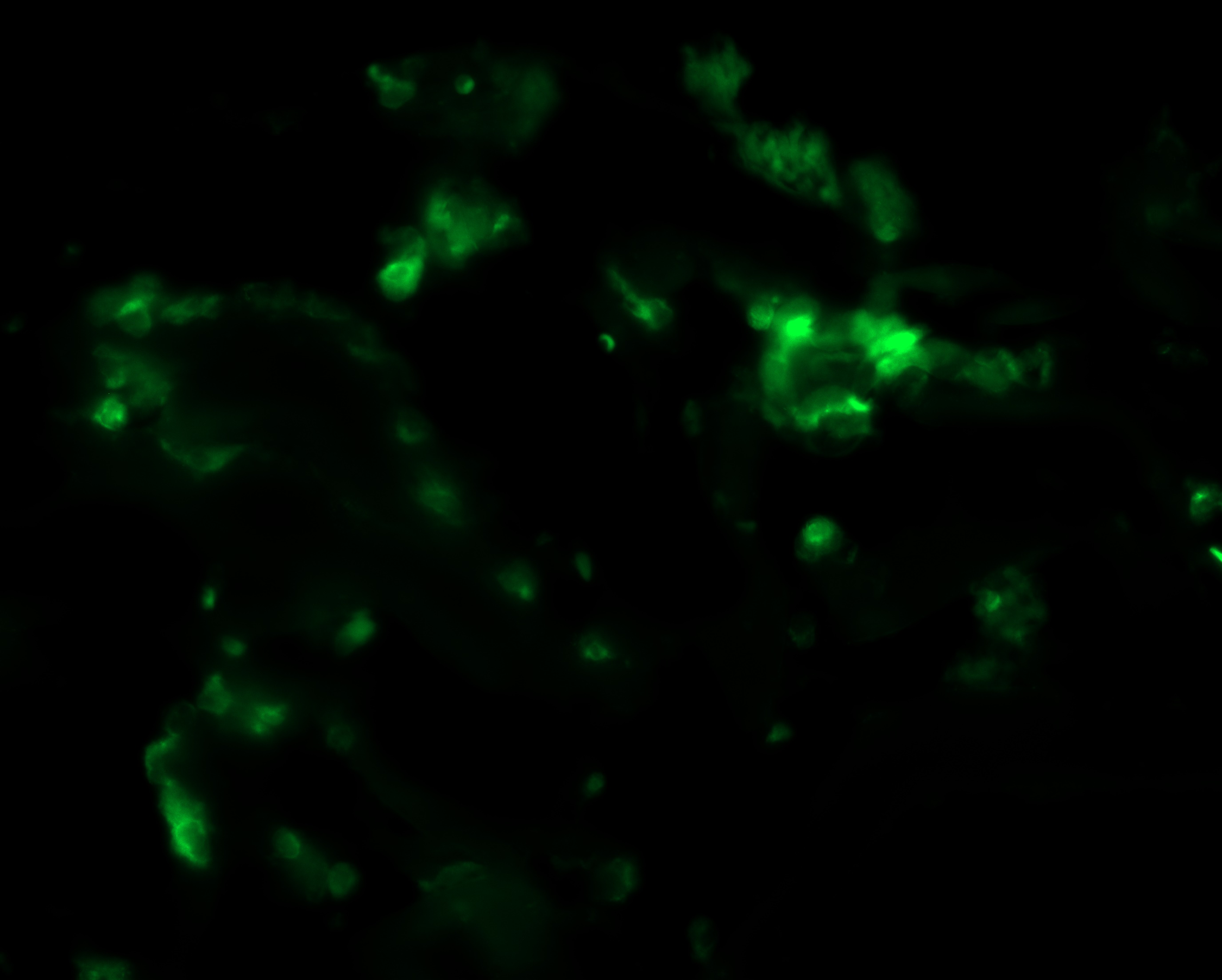

Supplement: S4 File — (ZIP) [file pone.0265049.s006.zip › IF/3D SF ITMSC Snap-398_Alexa Fluor 488.tif]

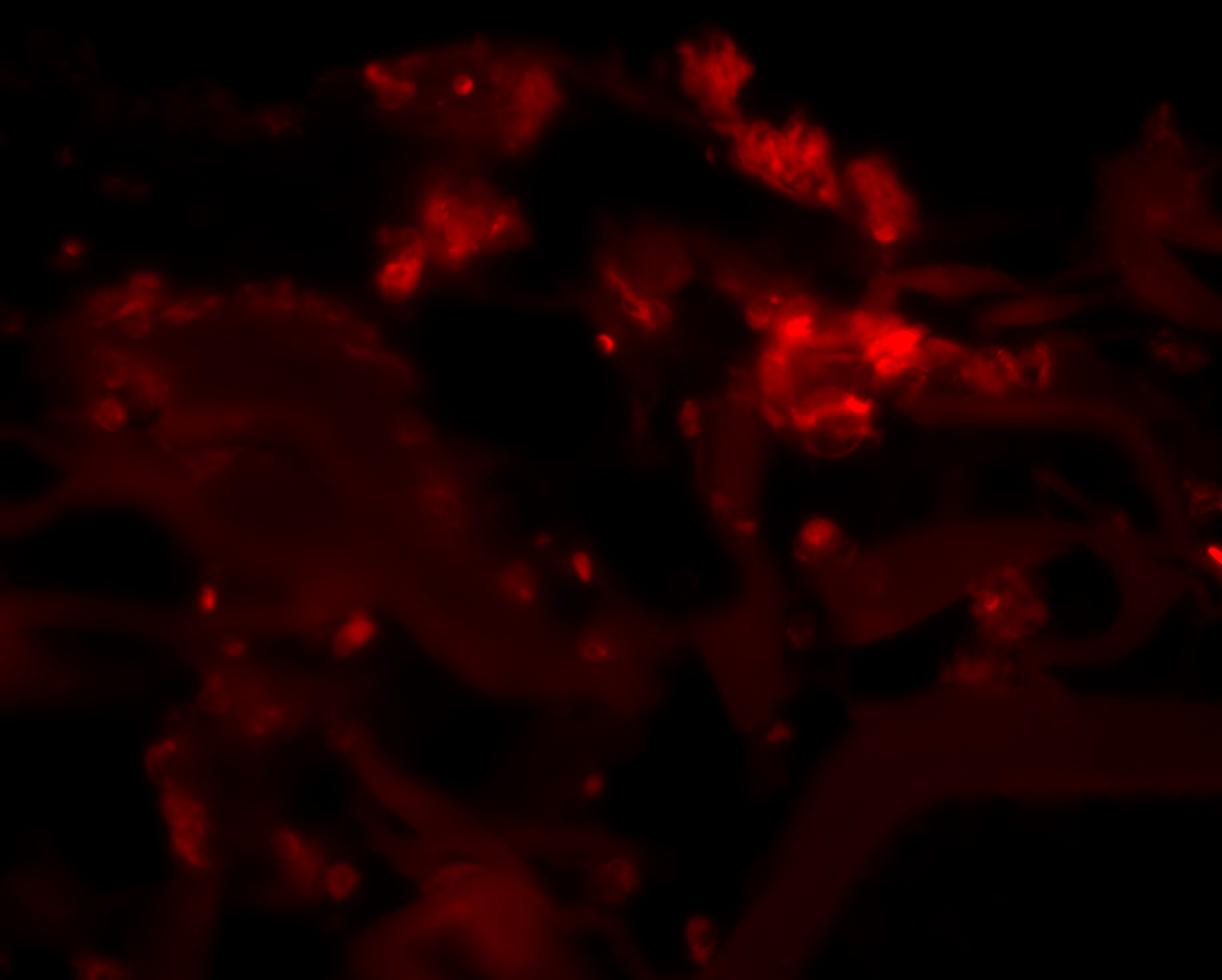

Supplement: S4 File — (ZIP) [file pone.0265049.s006.zip › IF/3D SF ITMSC Snap-398_Alexa Fluor 594.tif]

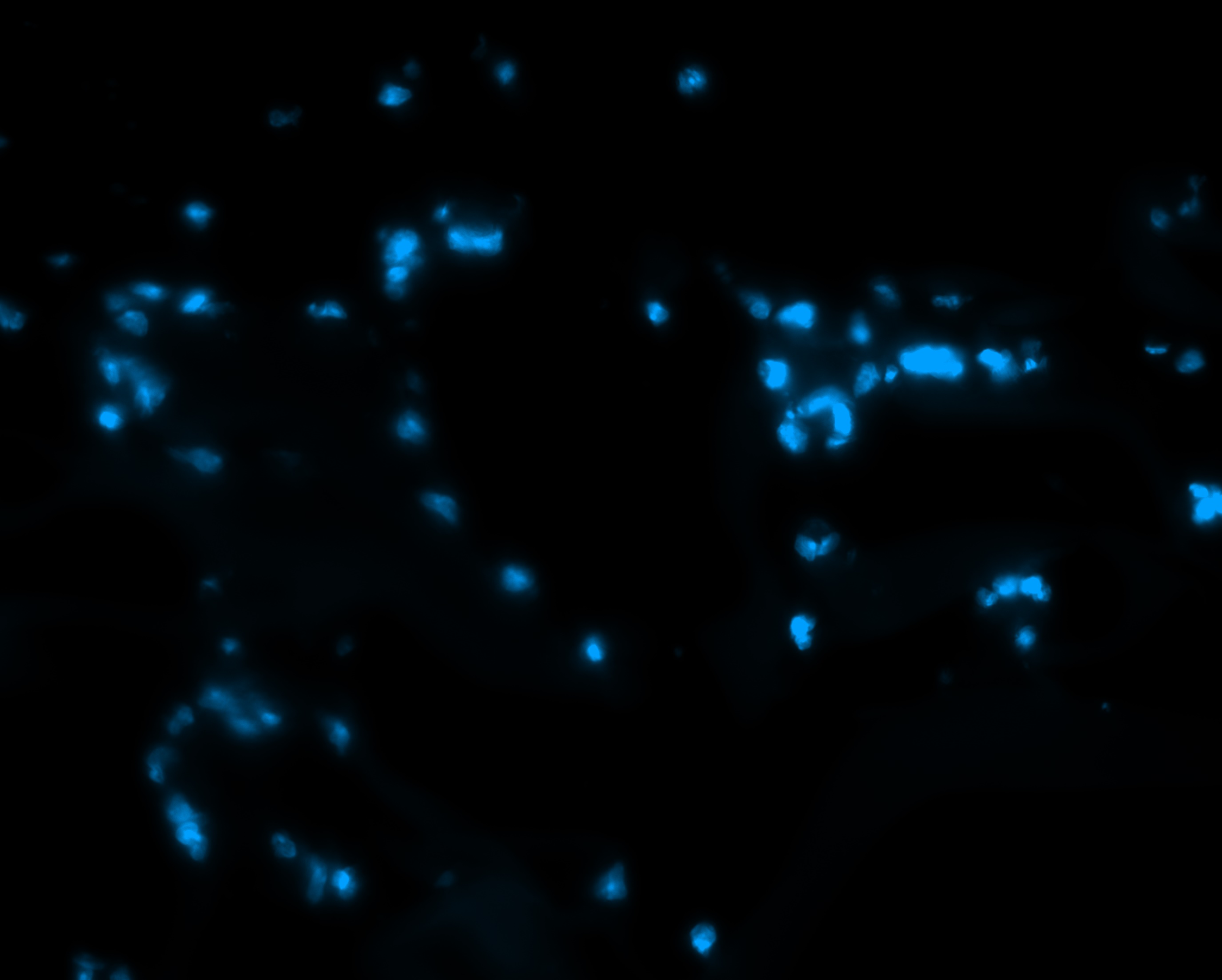

Supplement: S4 File — (ZIP) [file pone.0265049.s006.zip › IF/3D SF ITMSC Snap-398_DAPI.tif]

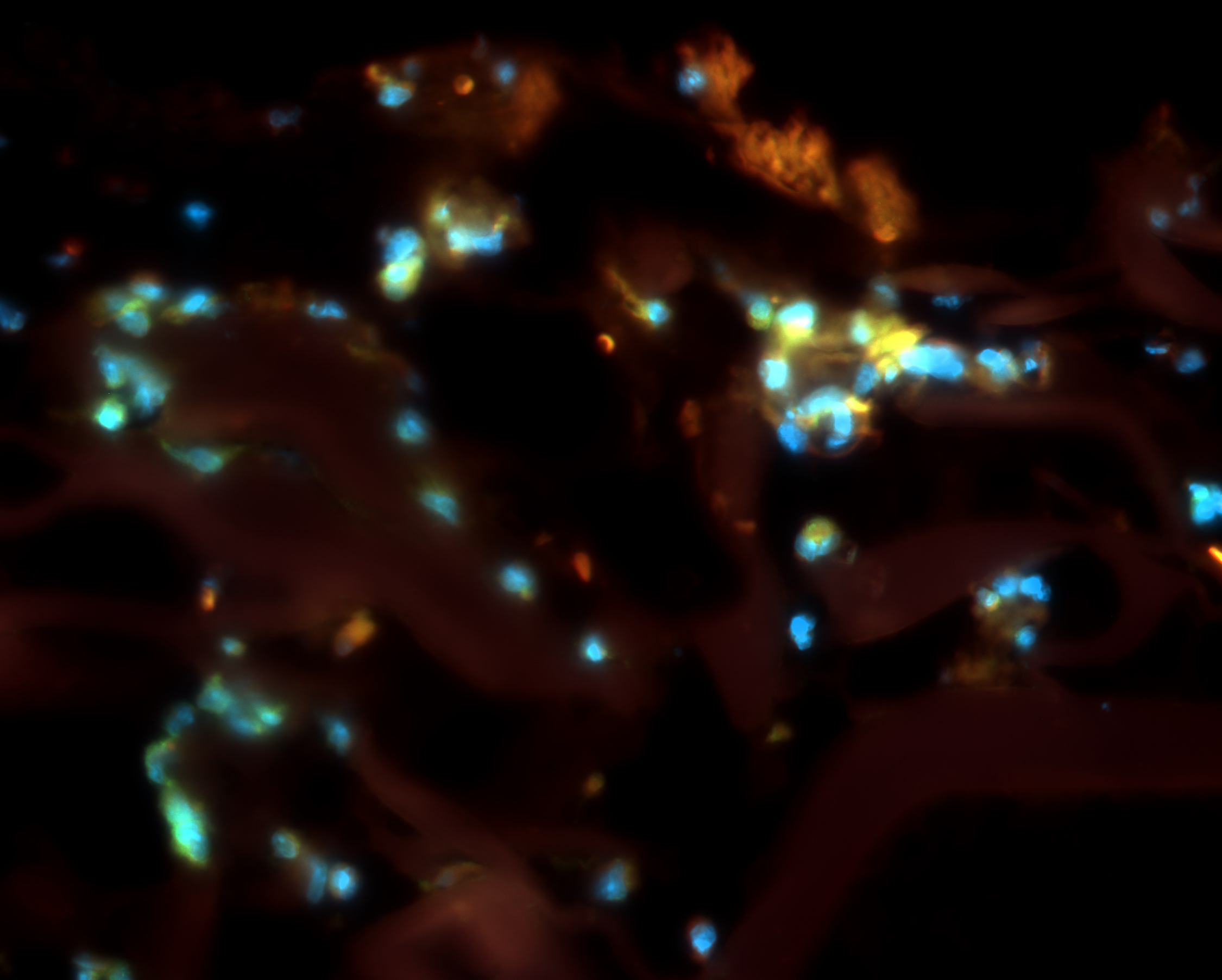

Supplement: S4 File — (ZIP) [file pone.0265049.s006.zip › IF/3D SF ITMSC Snap-398_Merge.tif]

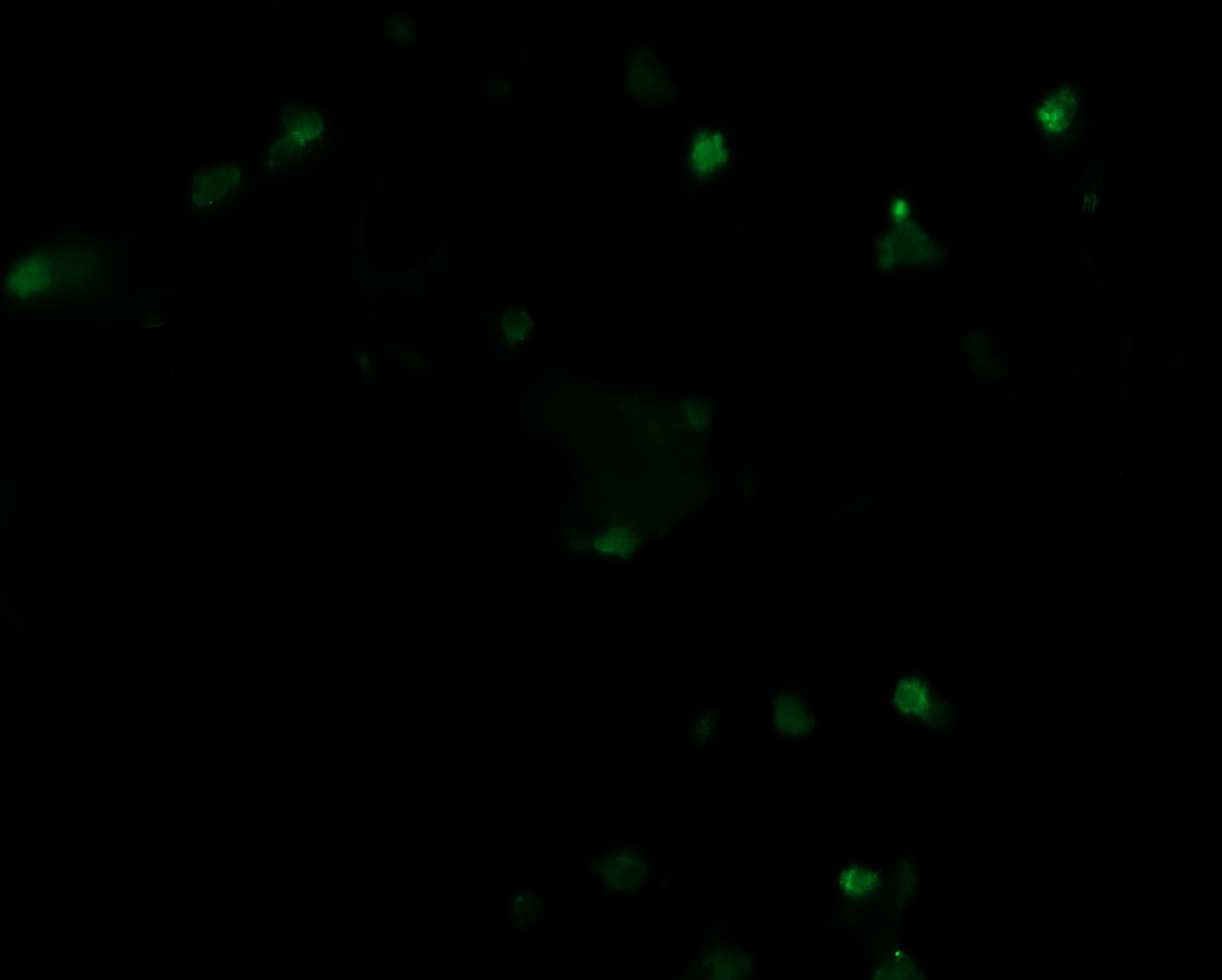

Supplement: S4 File — (ZIP) [file pone.0265049.s006.zip › IF/3D SF MSCSnap-416_Alexa Fluor 488-1.tif]

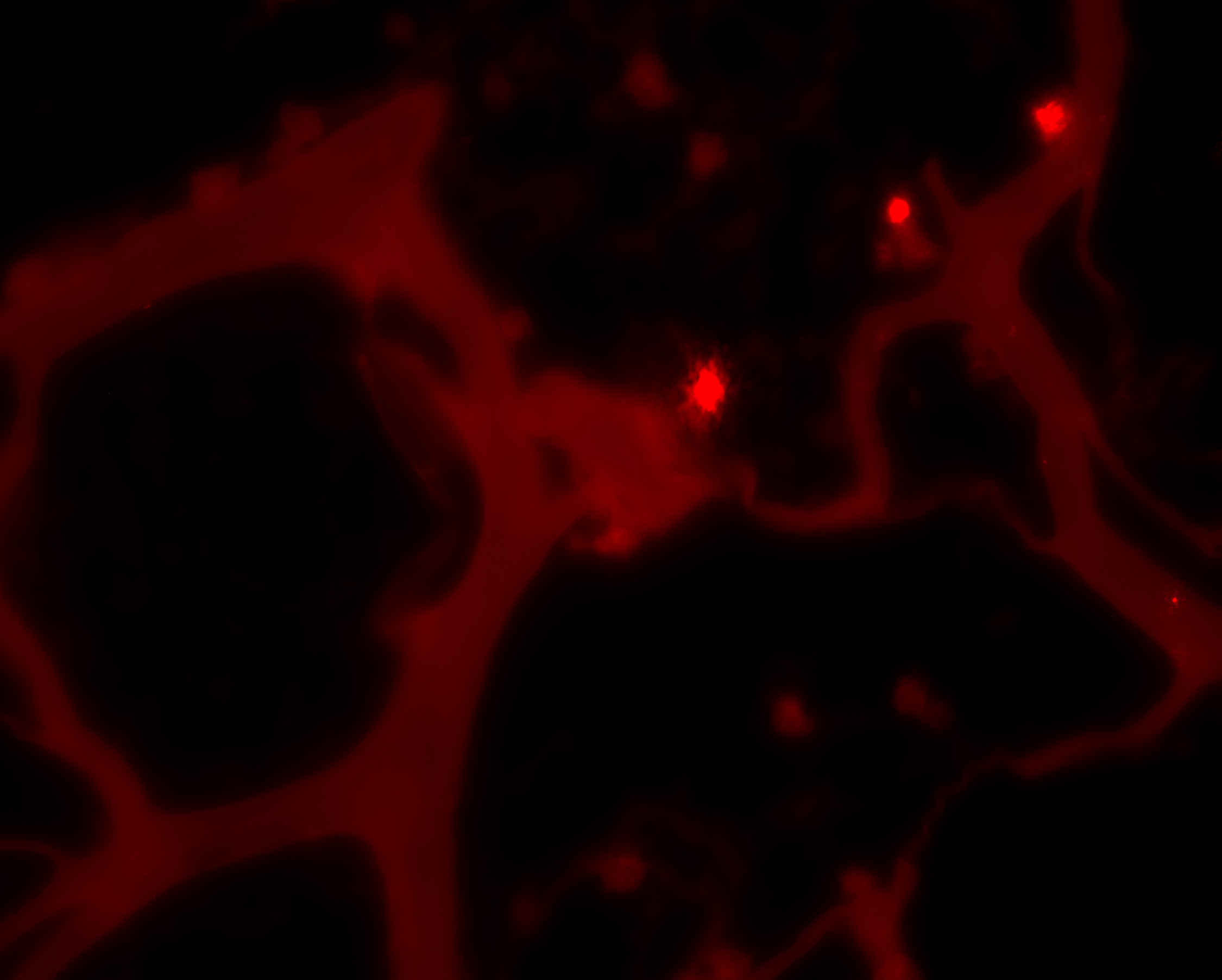

Supplement: S4 File — (ZIP) [file pone.0265049.s006.zip › IF/3D SF MSCSnap-416_Alexa Fluor 594.tif]

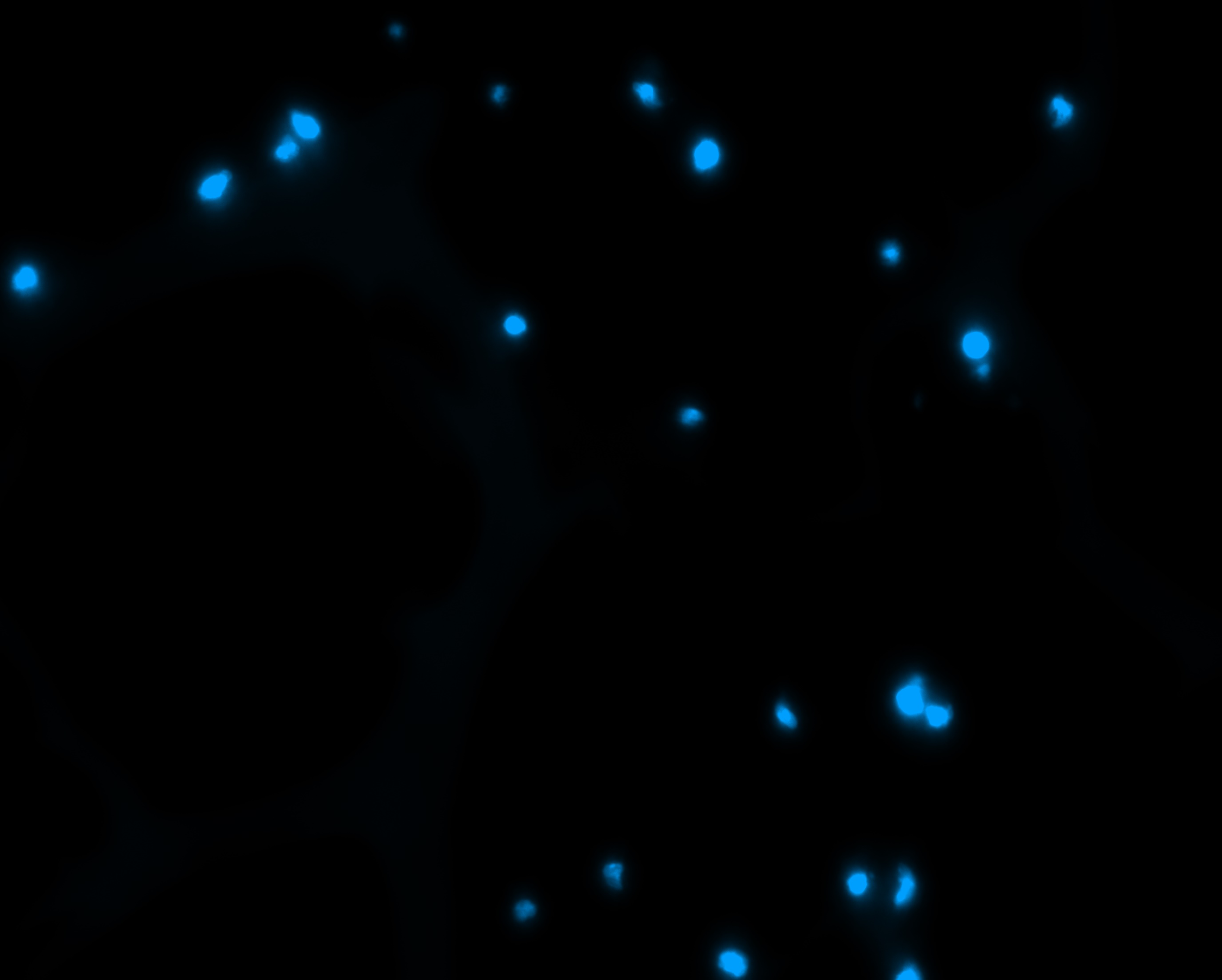

Supplement: S4 File — (ZIP) [file pone.0265049.s006.zip › IF/3D SF MSCSnap-416_DAPI.tif]

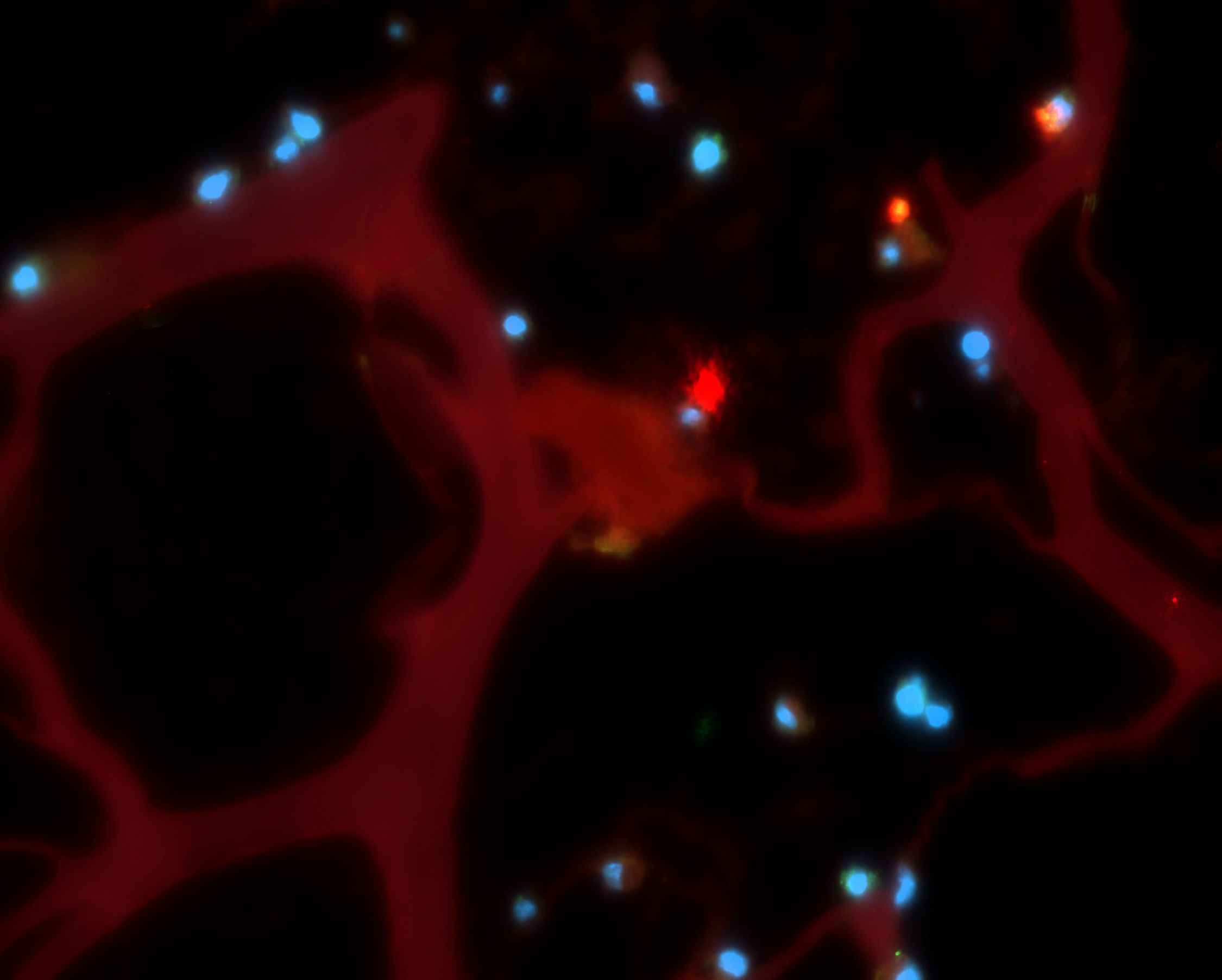

Supplement: S4 File — (ZIP) [file pone.0265049.s006.zip › IF/3D SF MSCSnap-416_Merge.tif]

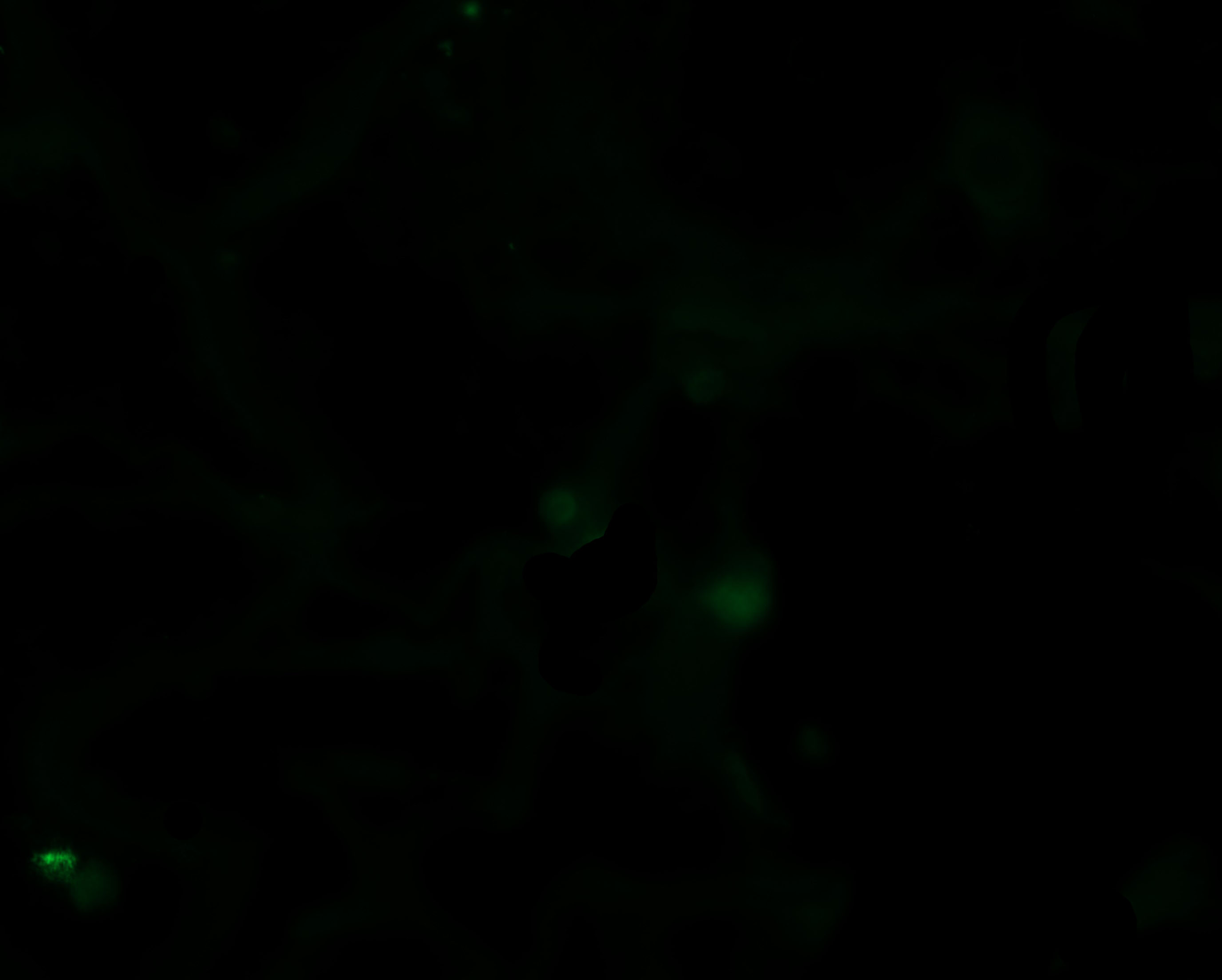

Supplement: S4 File — (ZIP) [file pone.0265049.s006.zip › IF/3D SF Snap-412_Alexa Fluor 488-1.tif]

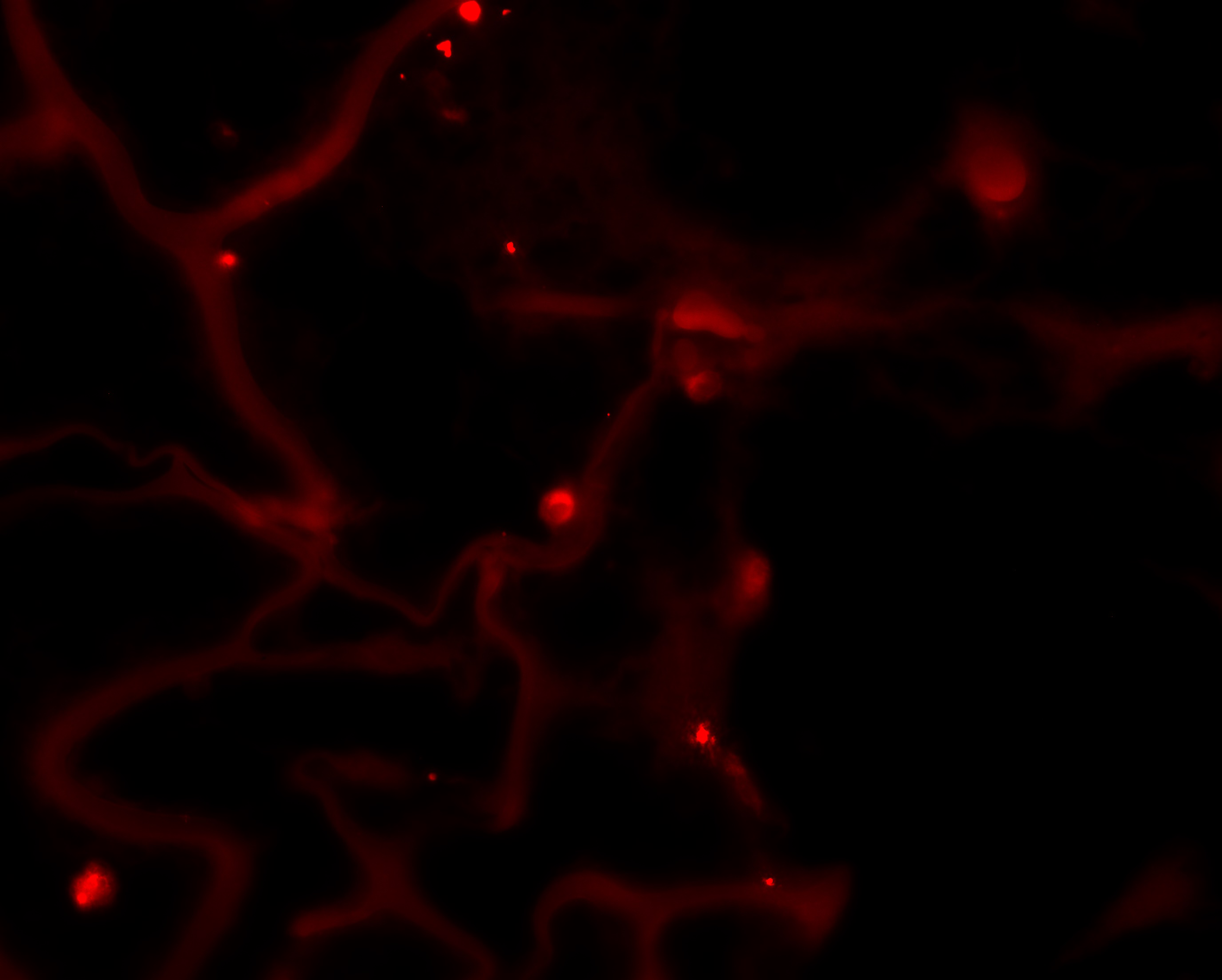

Supplement: S4 File — (ZIP) [file pone.0265049.s006.zip › IF/3D SF Snap-412_Alexa Fluor 594.tif]

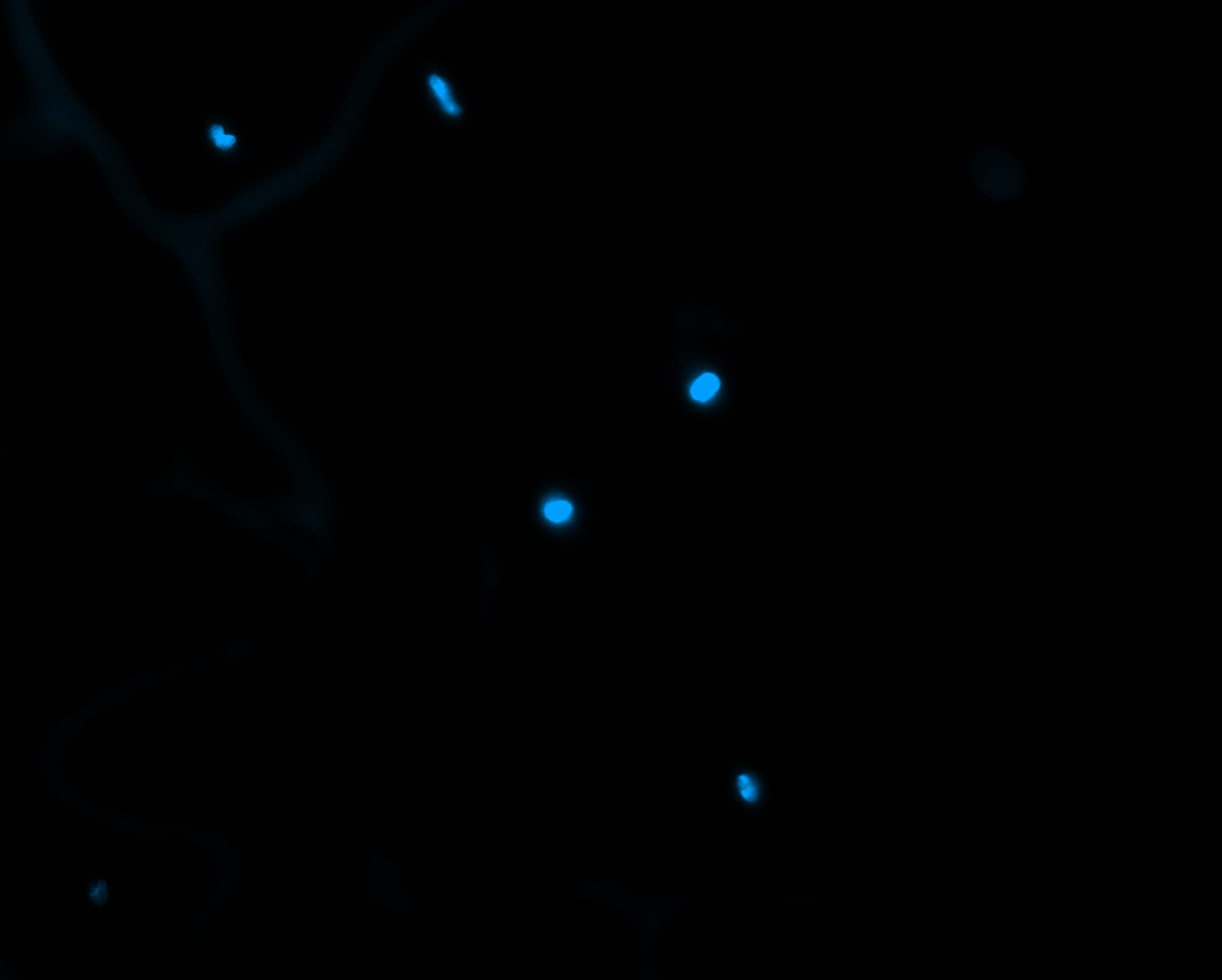

Supplement: S4 File — (ZIP) [file pone.0265049.s006.zip › IF/3D SF Snap-412_DAPI.tif]

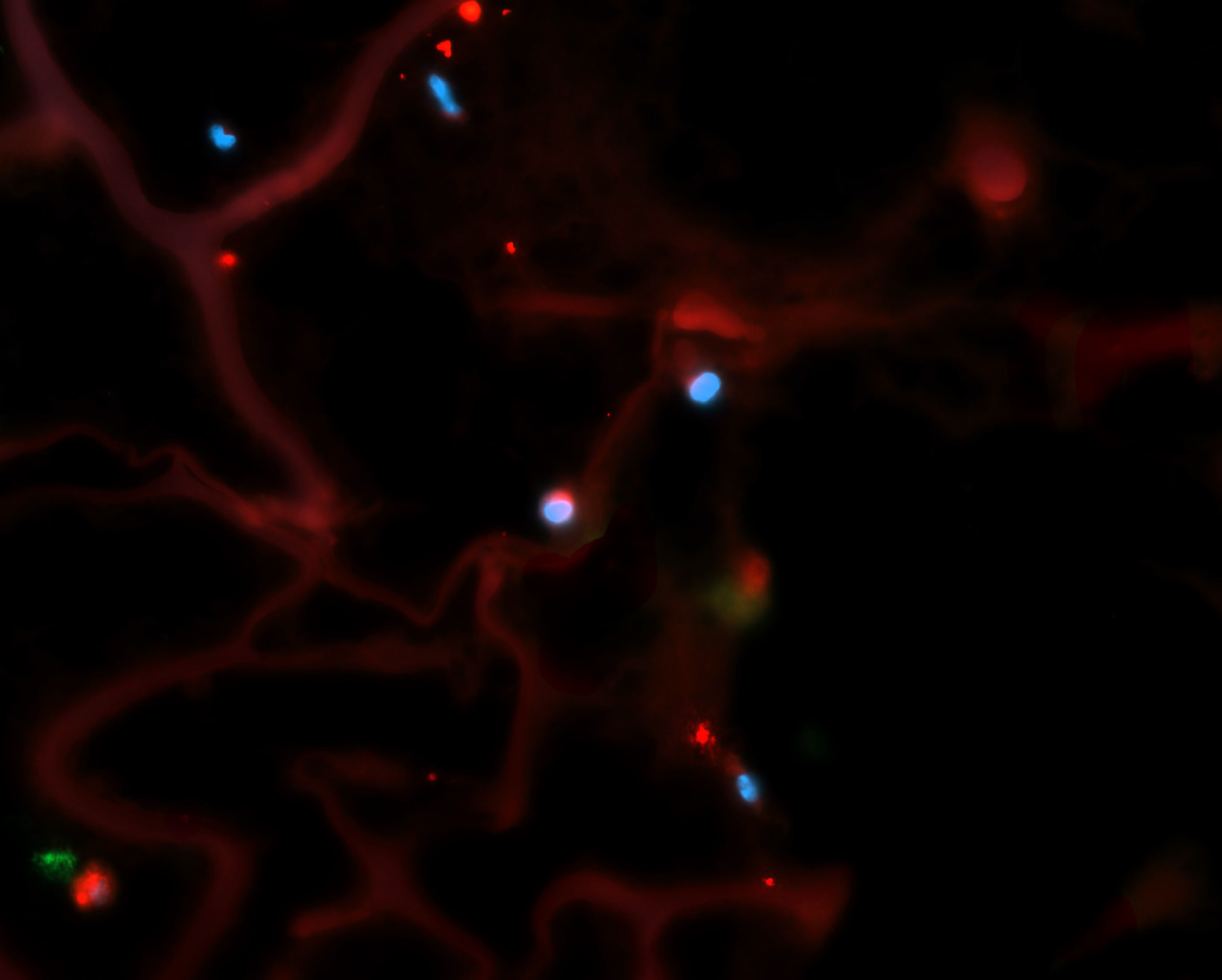

Supplement: S4 File — (ZIP) [file pone.0265049.s006.zip › IF/3D SF Snap-412_Merge.tif]

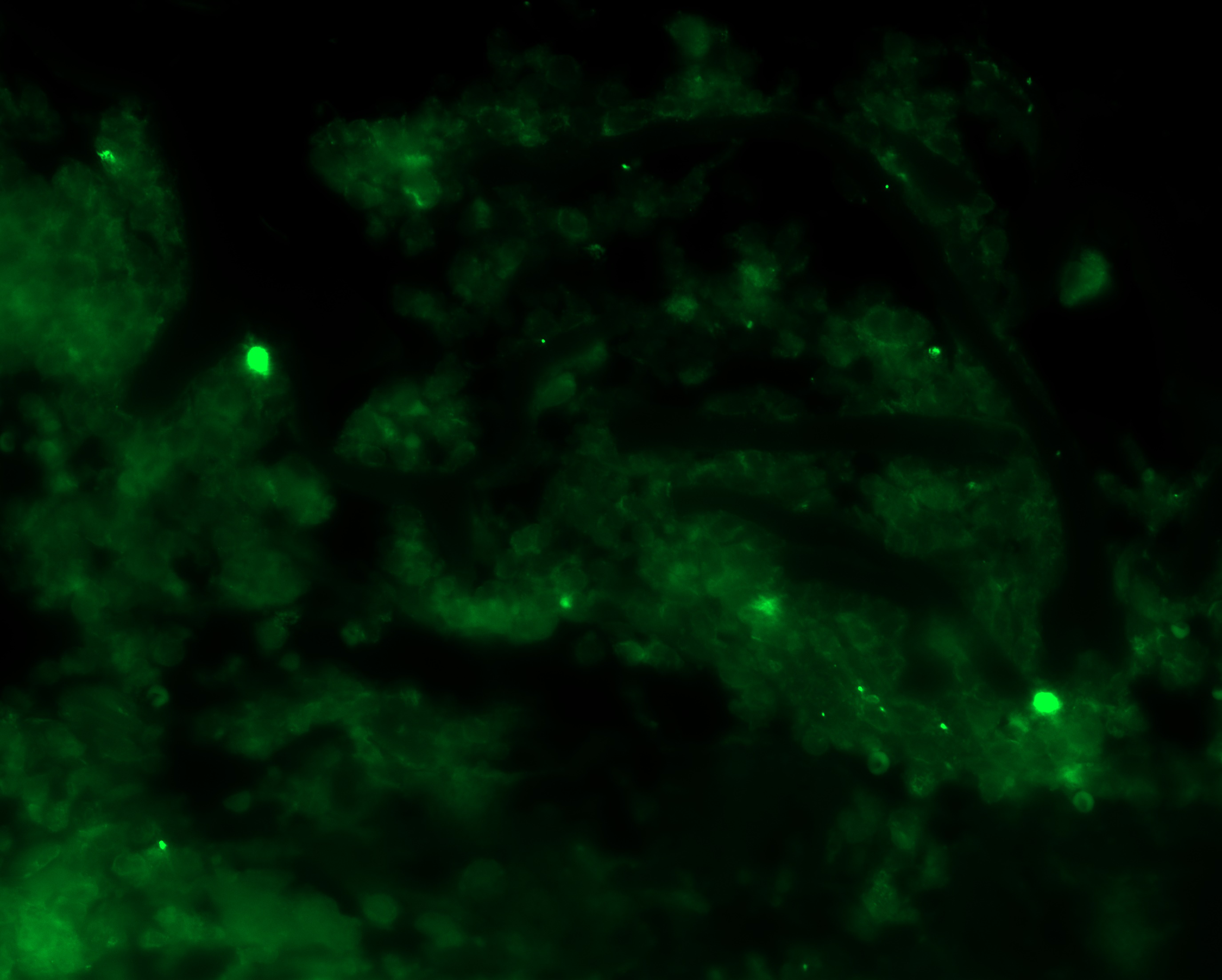

Supplement: S4 File — (ZIP) [file pone.0265049.s006.zip › IF/7D SF IT MSC Snap-378_Alexa Fluor 488.tif]

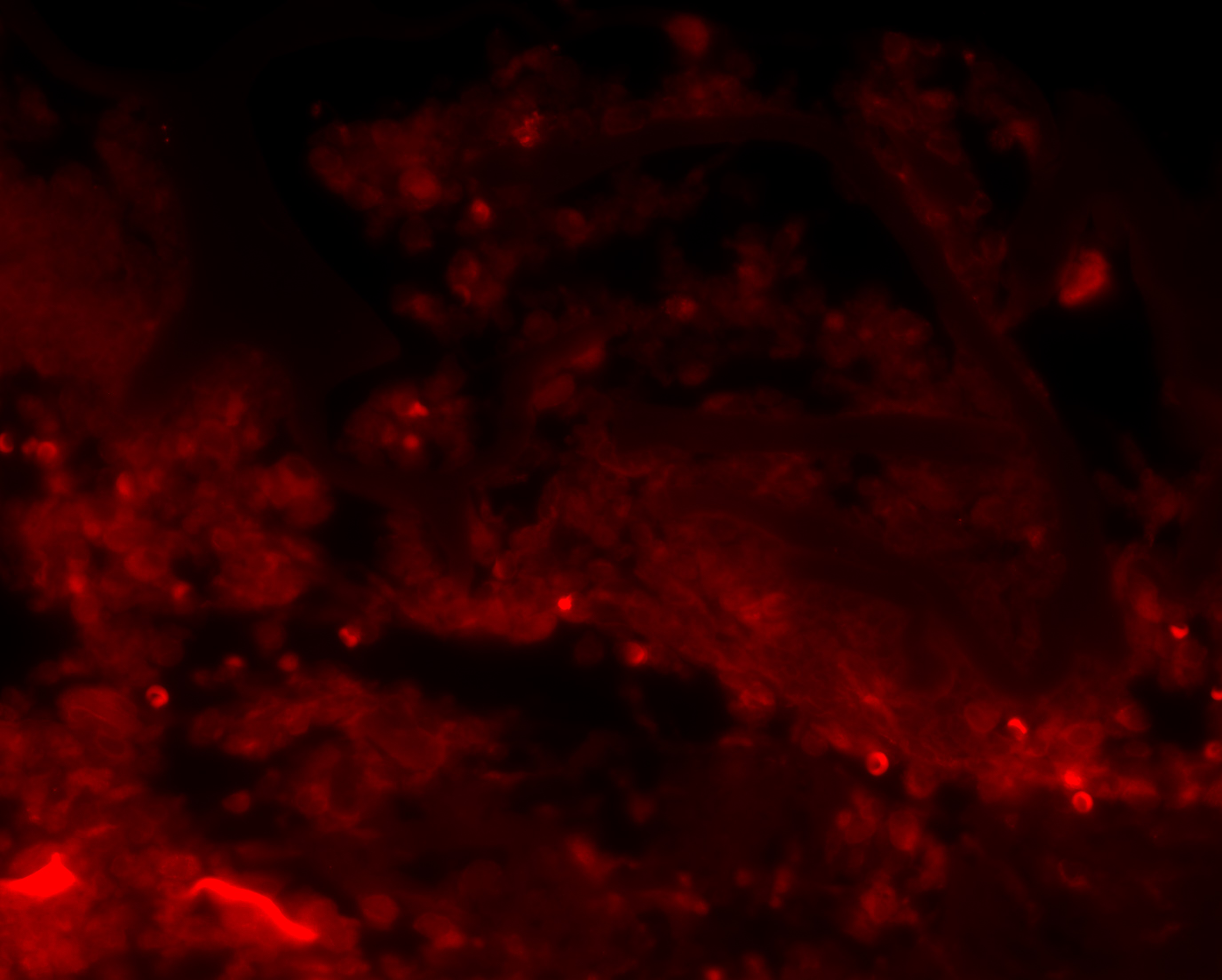

Supplement: S4 File — (ZIP) [file pone.0265049.s006.zip › IF/7D SF IT MSC Snap-378_Alexa Fluor 594.tif]

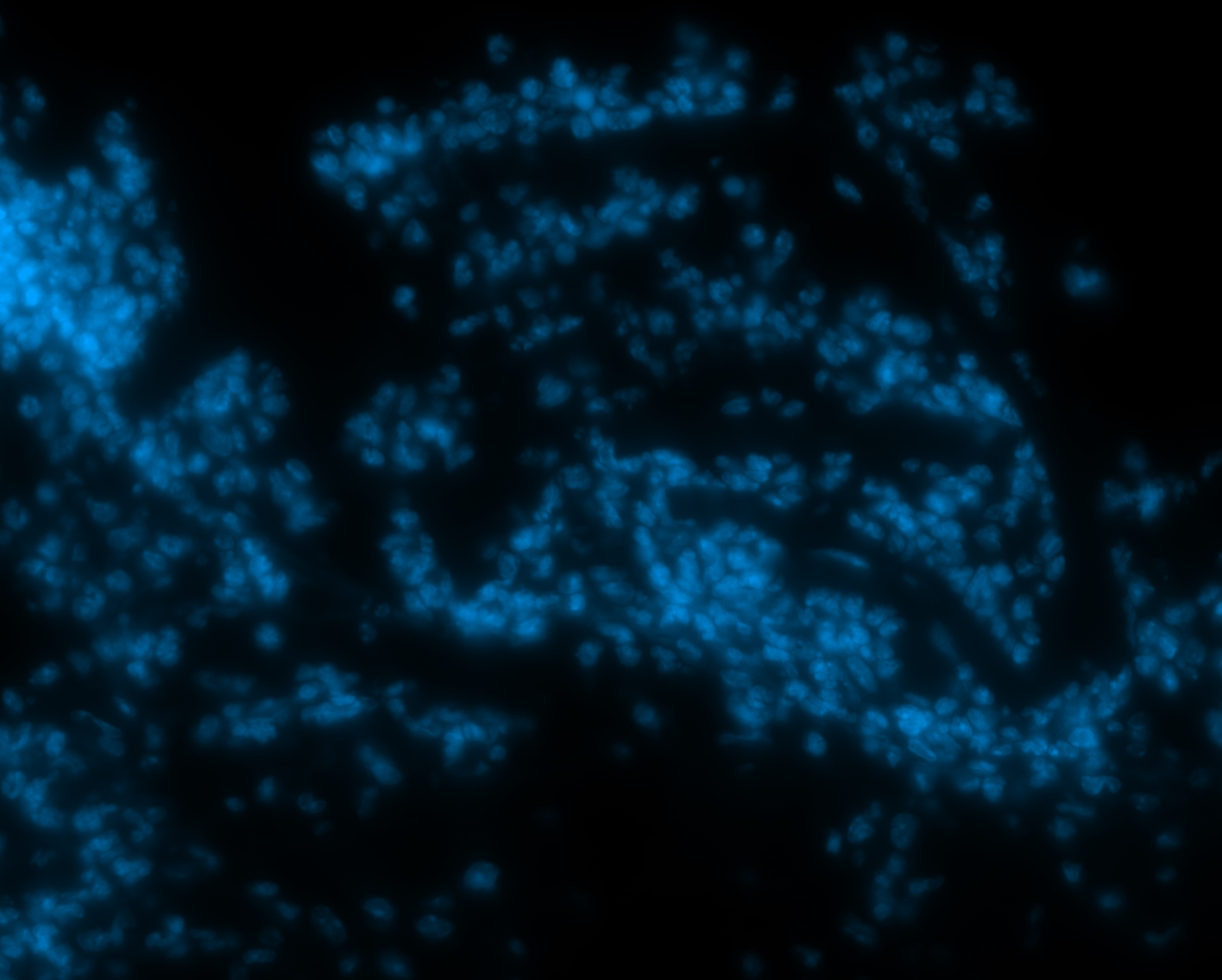

Supplement: S4 File — (ZIP) [file pone.0265049.s006.zip › IF/7D SF IT MSC Snap-378_DAPI.tif]

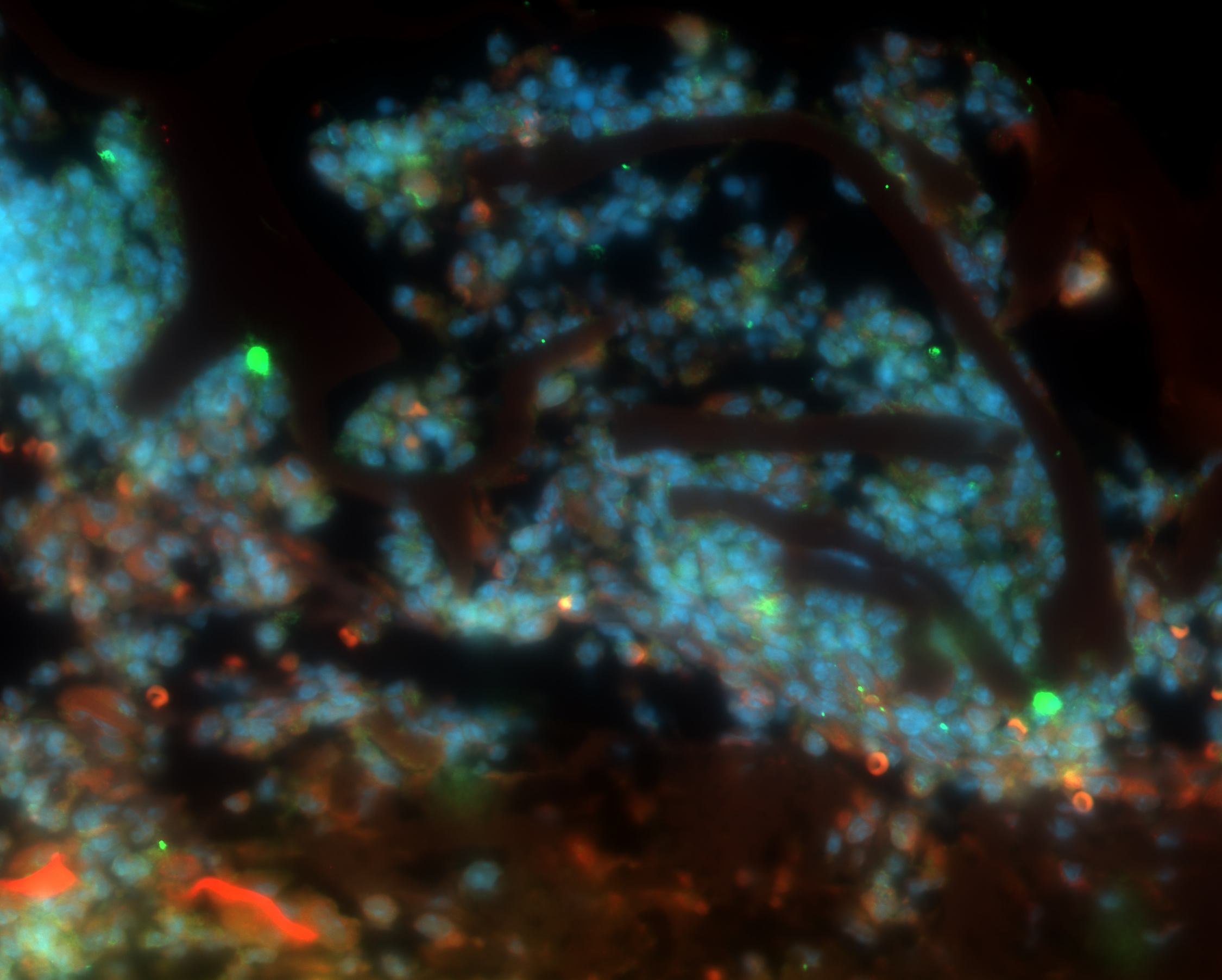

Supplement: S4 File — (ZIP) [file pone.0265049.s006.zip › IF/7D SF IT MSC Snap-378_Merge.tif]

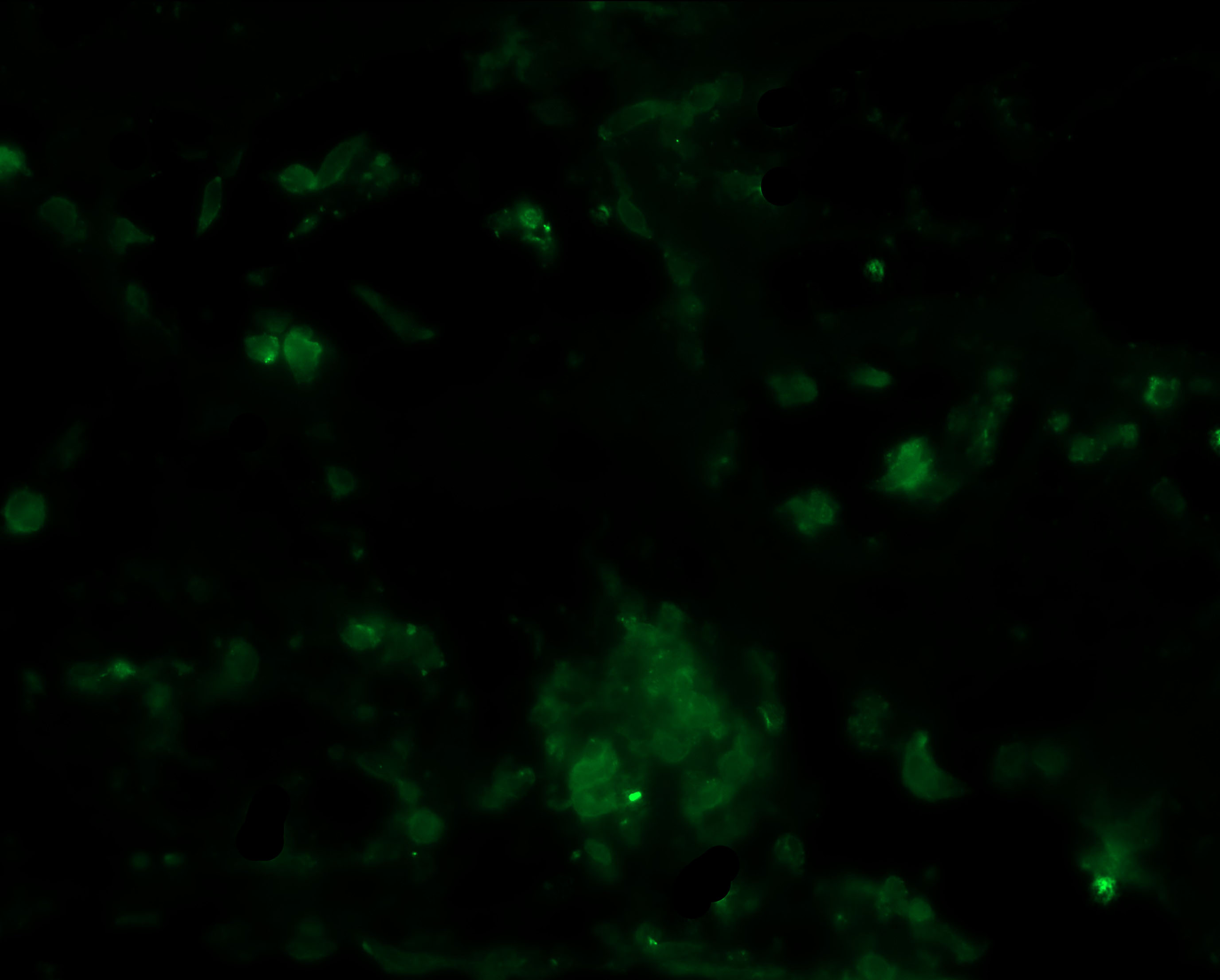

Supplement: S4 File — (ZIP) [file pone.0265049.s006.zip › IF/7D SF MSC Snap-388_Alexa Fluor 488-1.tif]

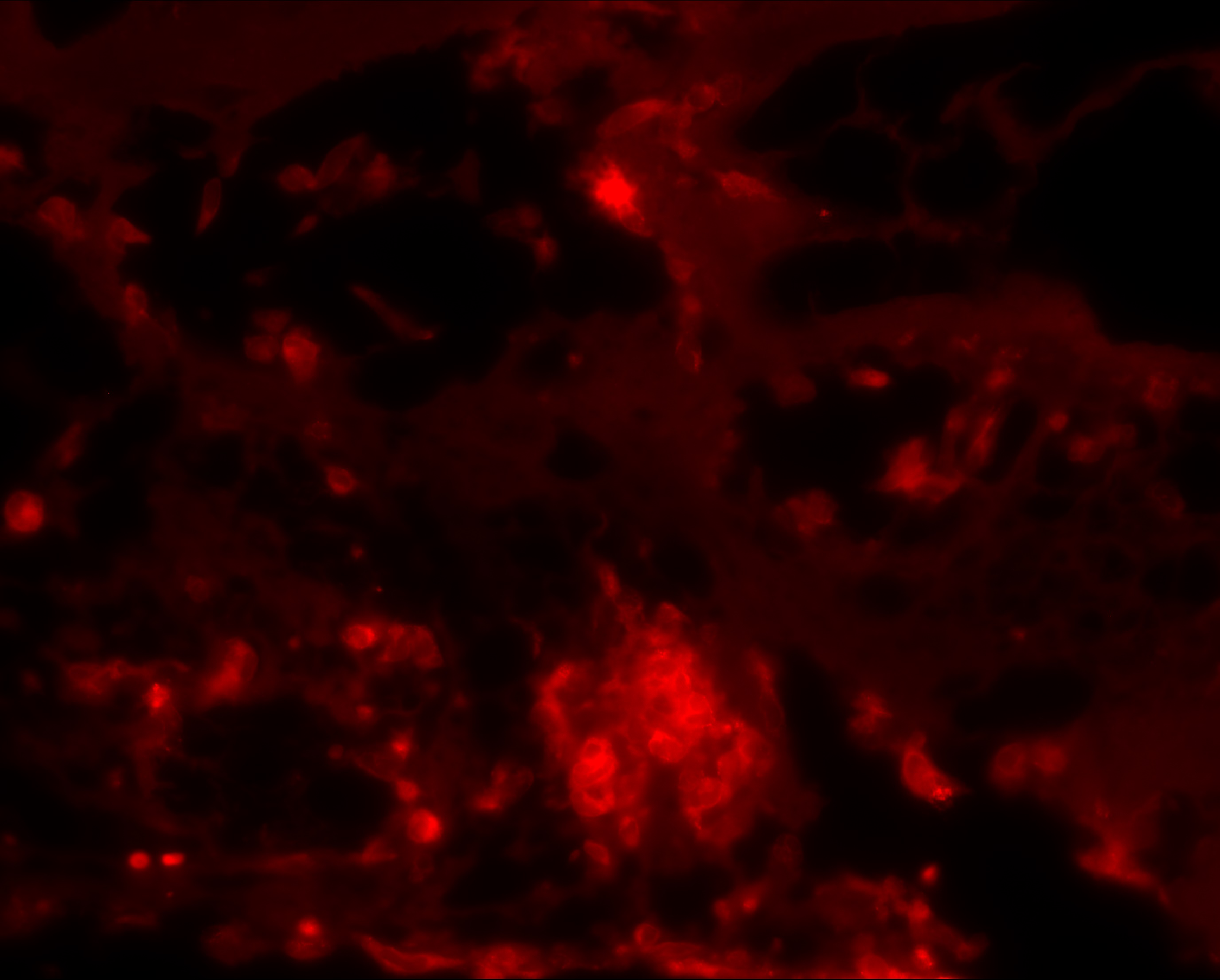

Supplement: S4 File — (ZIP) [file pone.0265049.s006.zip › IF/7D SF MSC Snap-388_Alexa Fluor 594.tif]

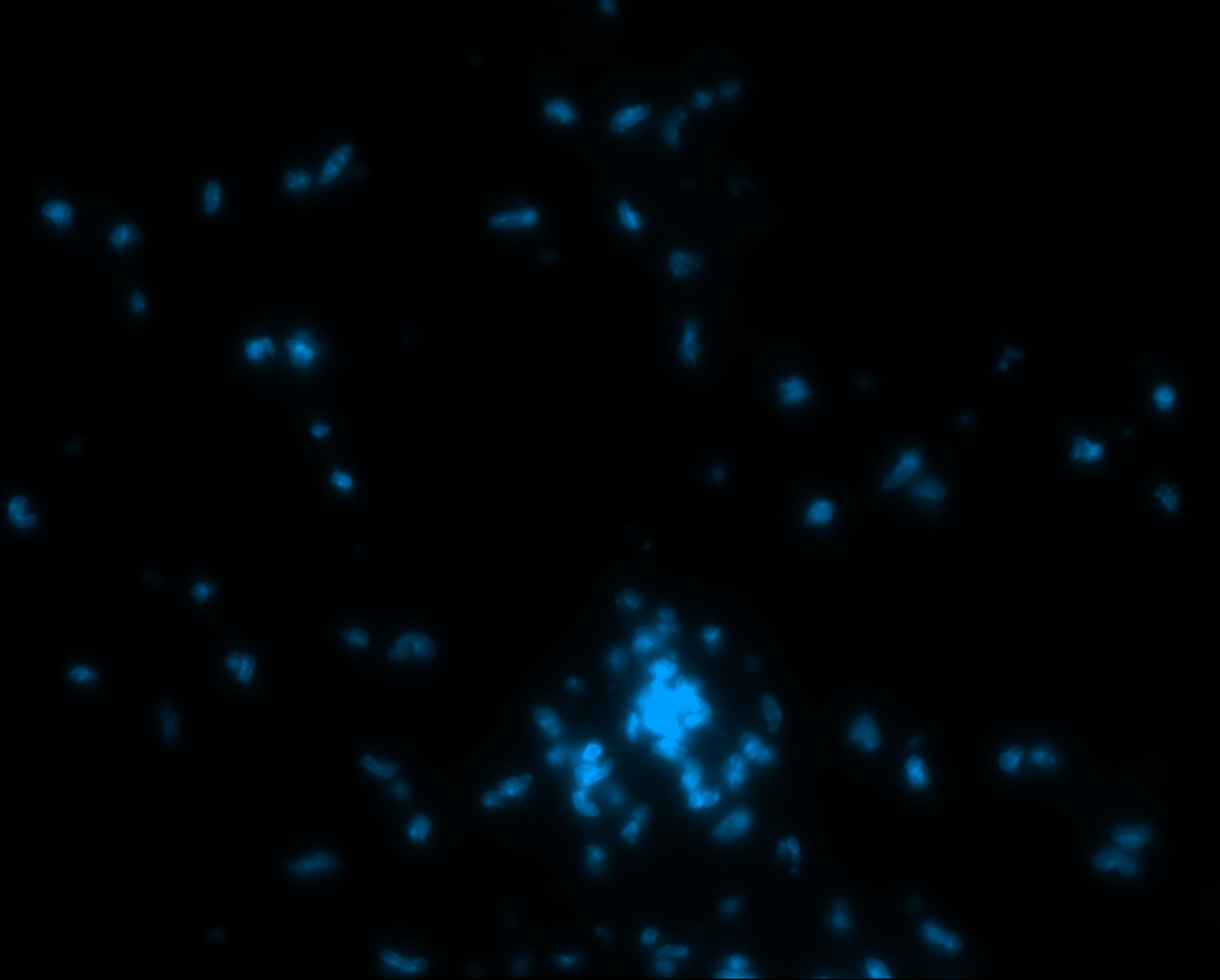

Supplement: S4 File — (ZIP) [file pone.0265049.s006.zip › IF/7D SF MSC Snap-388_DAPI.tif]

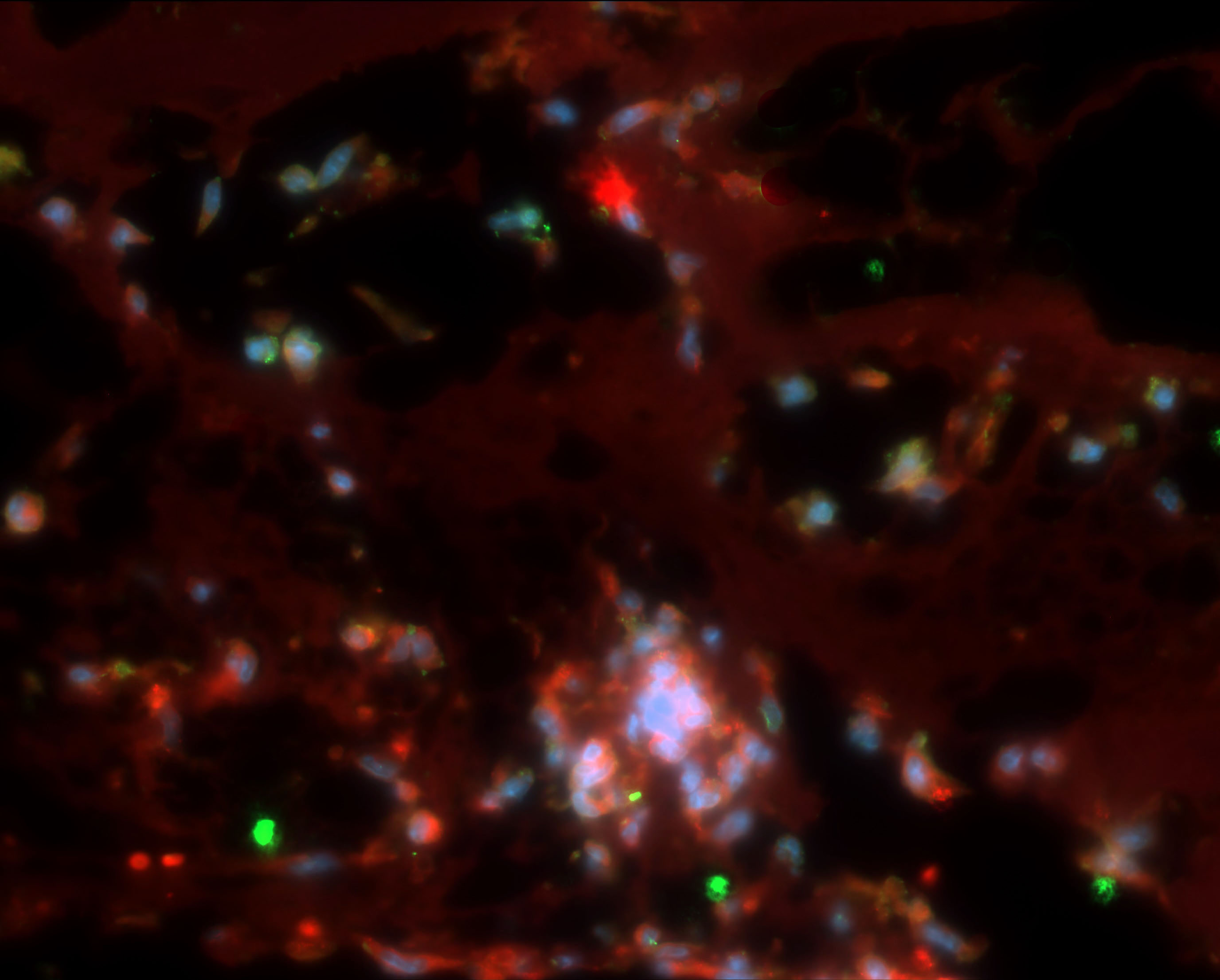

Supplement: S4 File — (ZIP) [file pone.0265049.s006.zip › IF/7D SF MSC Snap-Merge.tif]

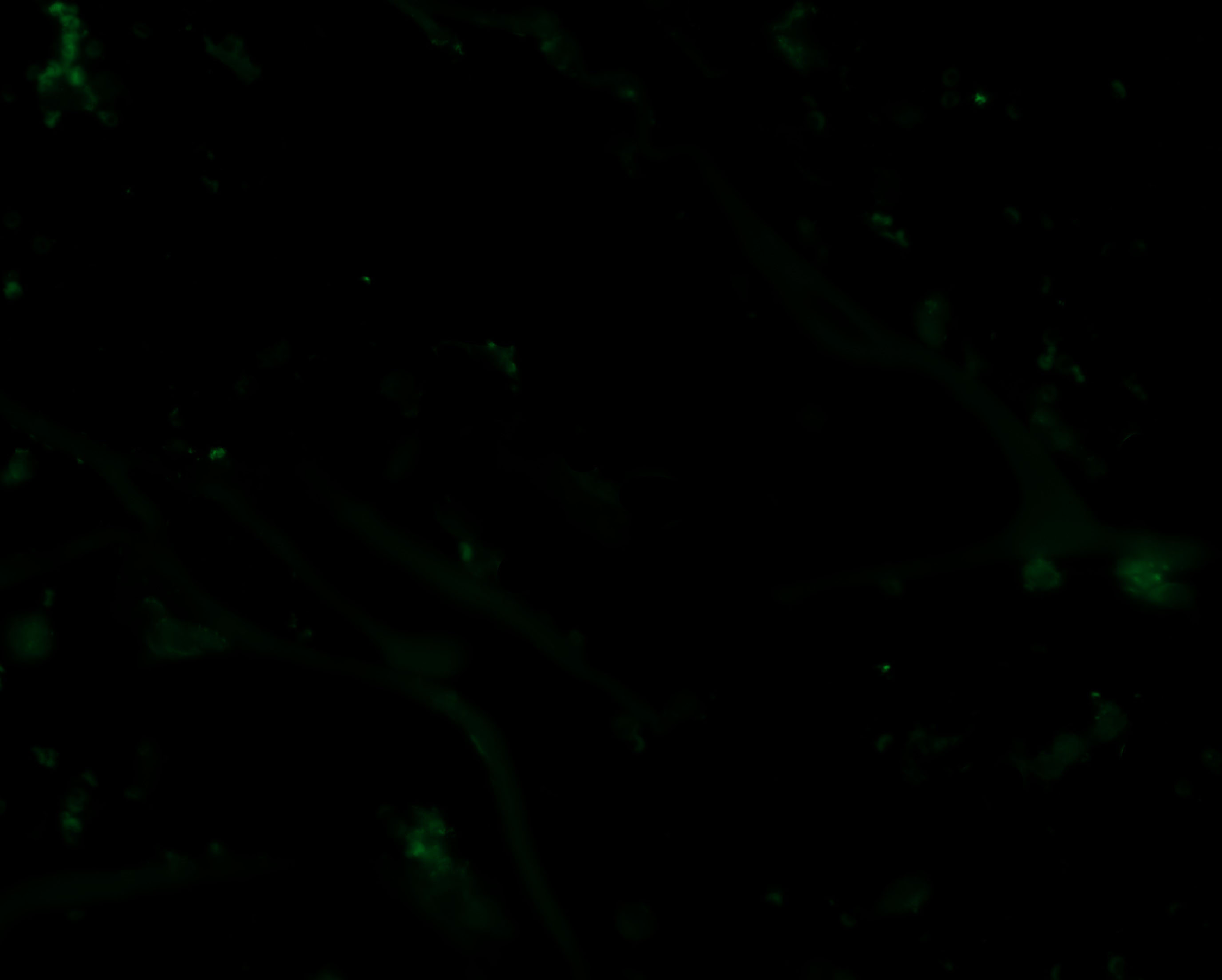

Supplement: S4 File — (ZIP) [file pone.0265049.s006.zip › IF/7D SF Snap-369_Alexa Fluor 488-1.tif]

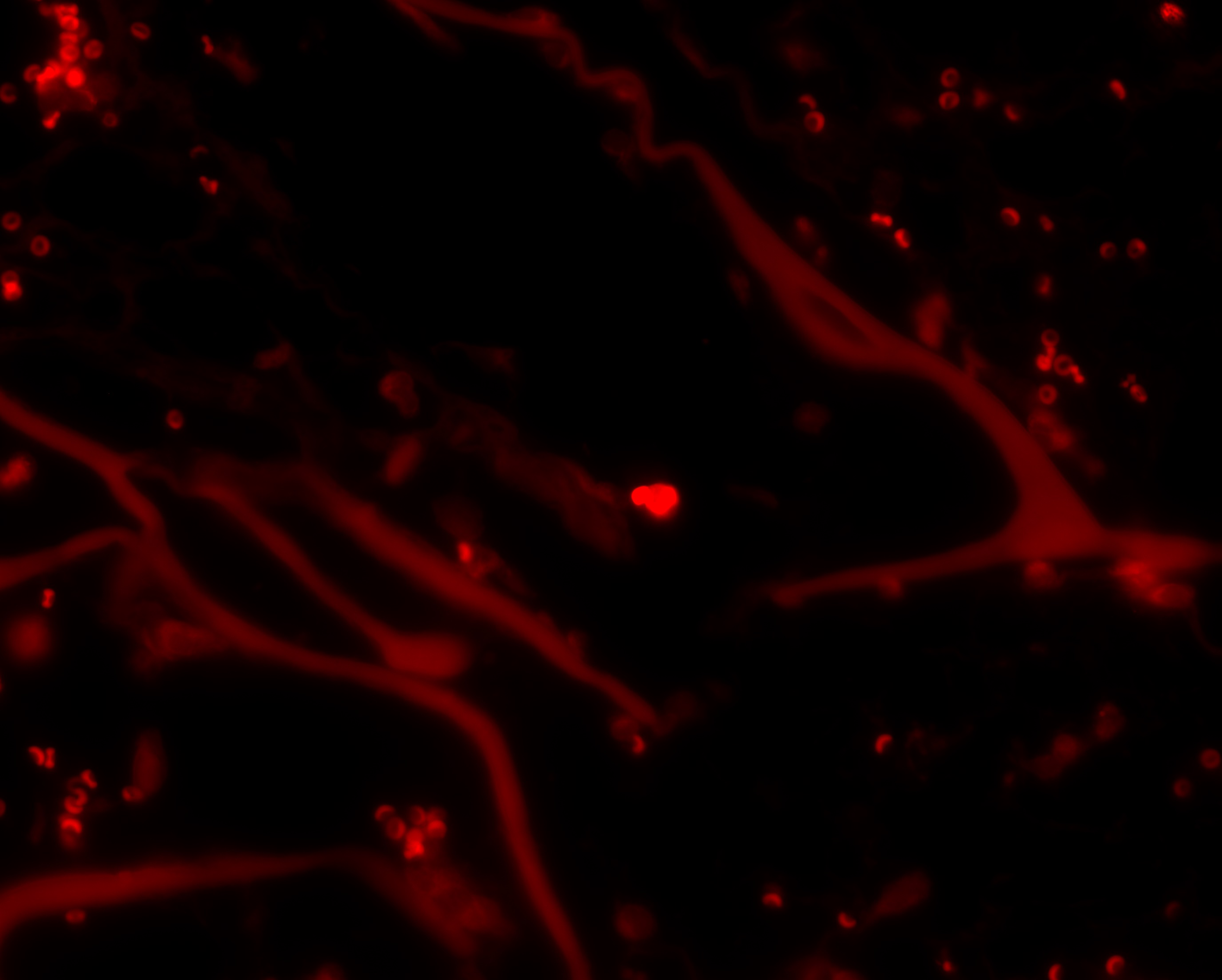

Supplement: S4 File — (ZIP) [file pone.0265049.s006.zip › IF/7D SF Snap-369_Alexa Fluor 594.tif]

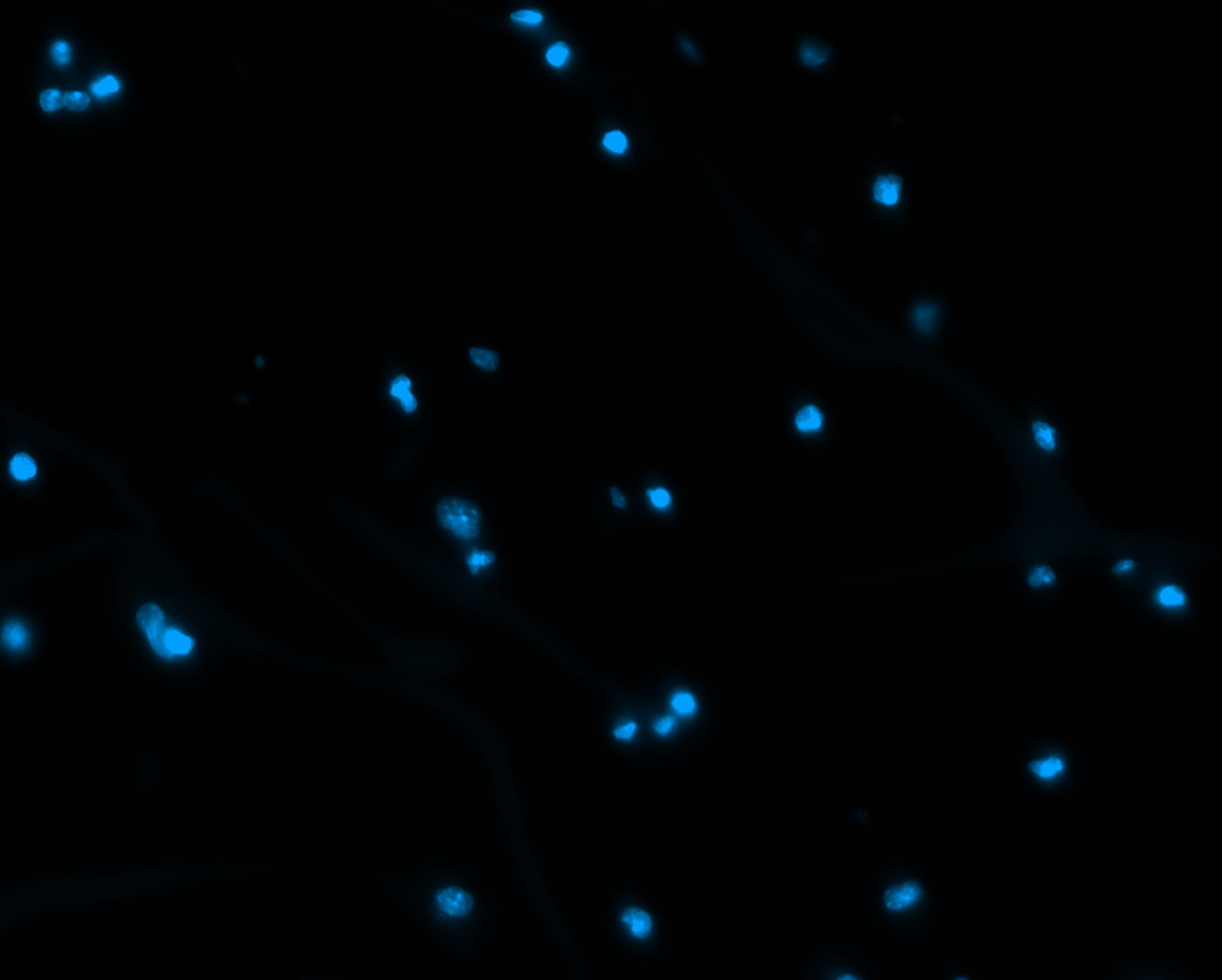

Supplement: S4 File — (ZIP) [file pone.0265049.s006.zip › IF/7D SF Snap-369_DAPI.tif]

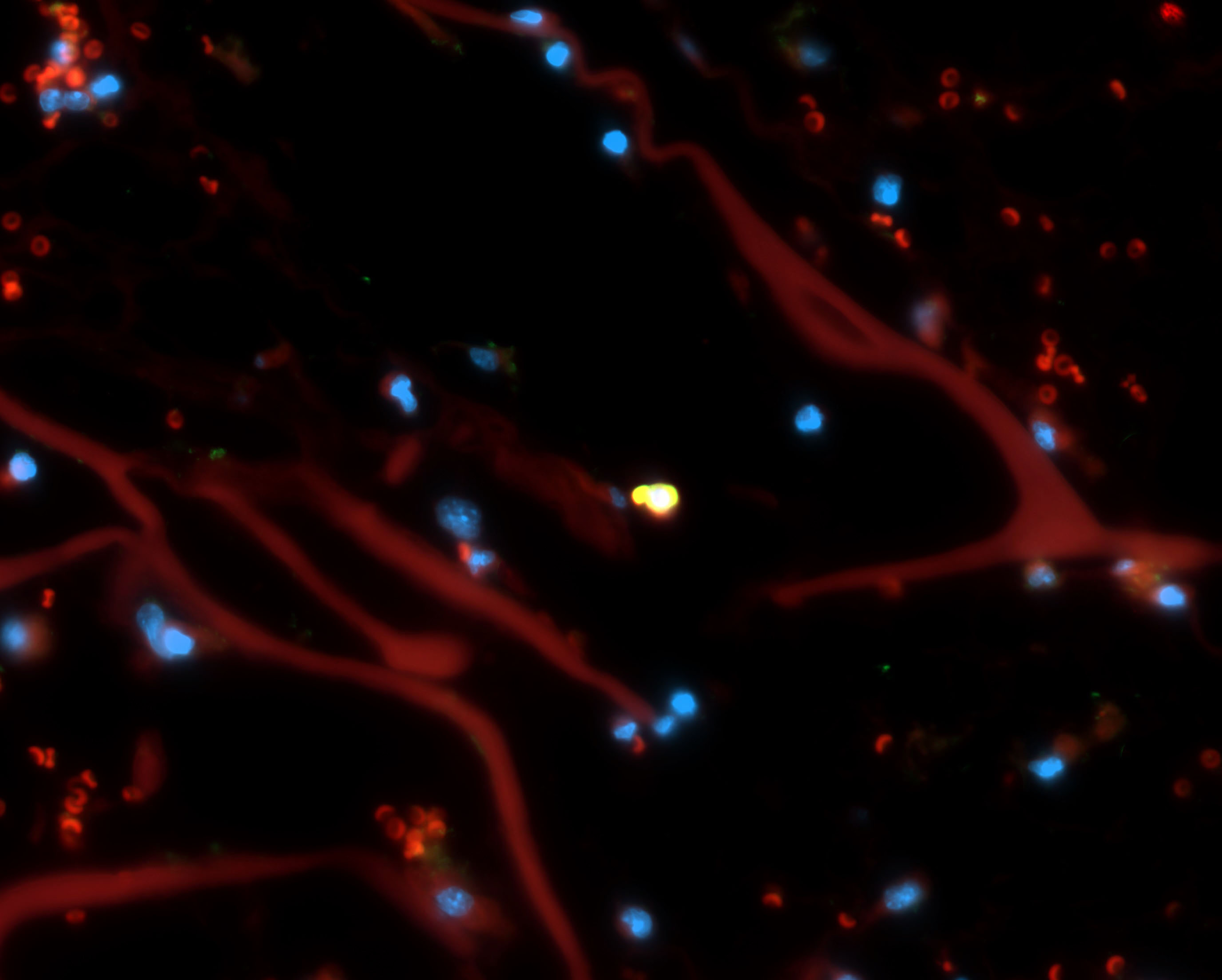

Supplement: S4 File — (ZIP) [file pone.0265049.s006.zip › IF/7D SF Snap-369_Merge.tif]

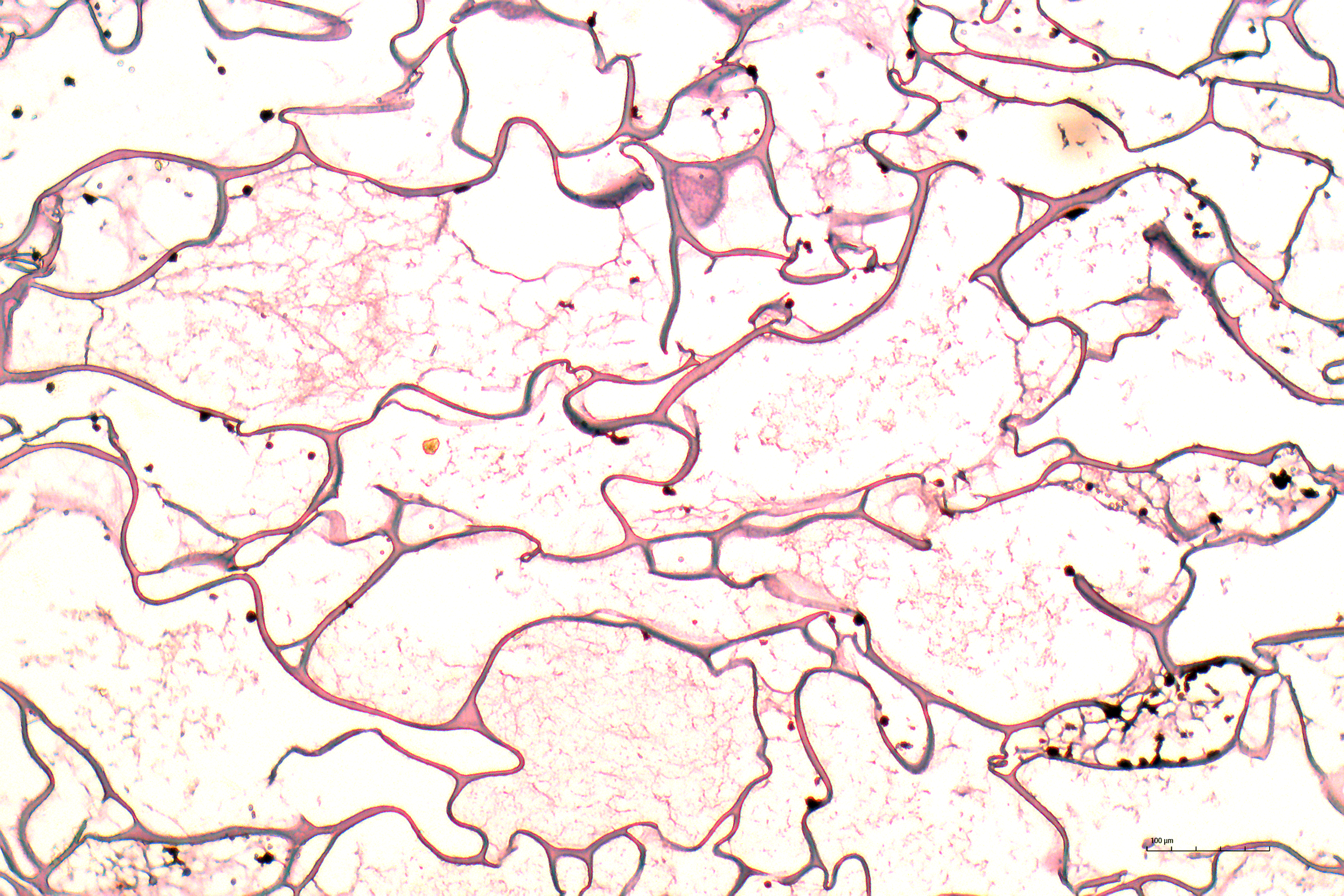

Supplement: S5 File — (ZIP) [file pone.0265049.s007.zip › IHC/SF 3D.tif]

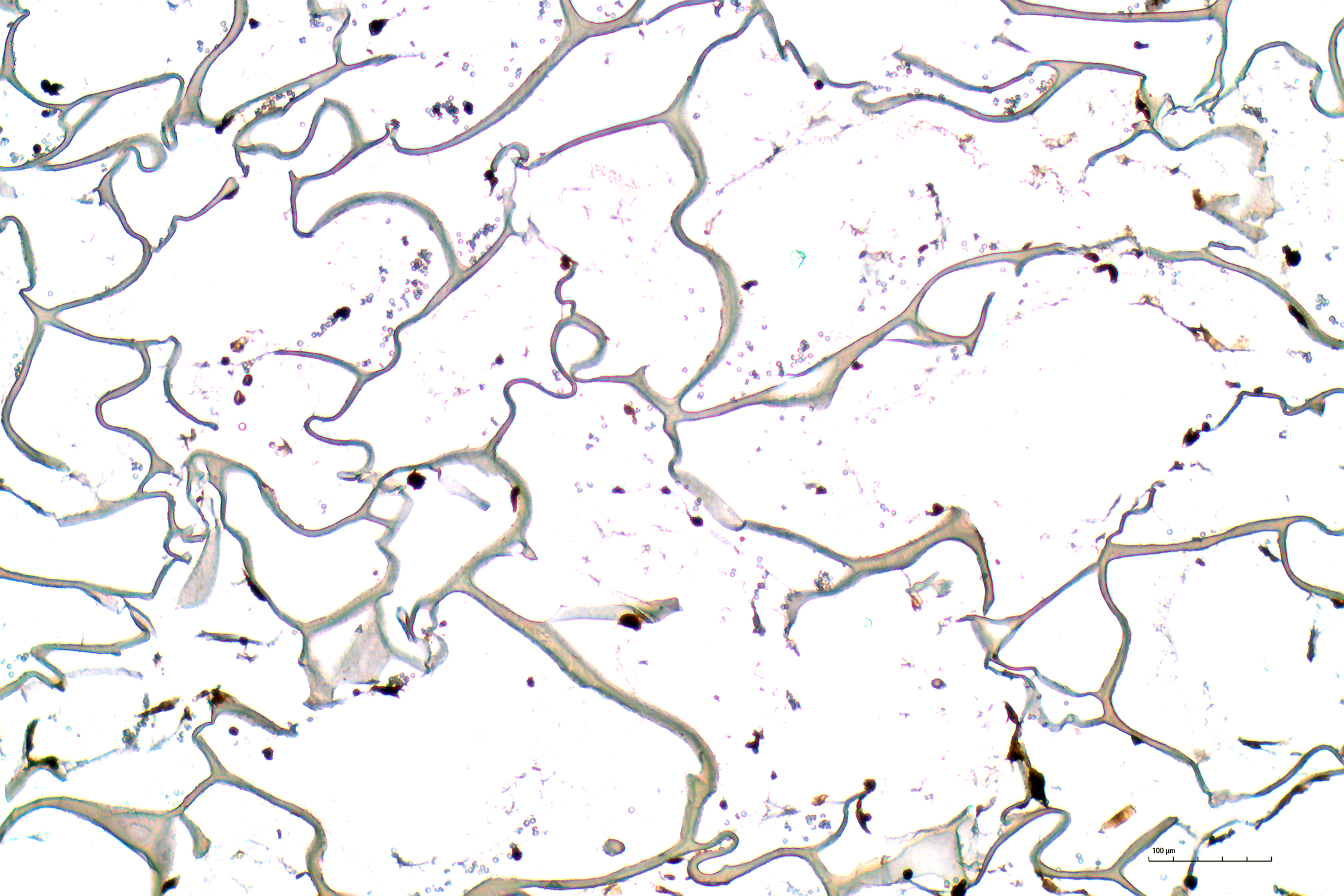

Supplement: S5 File — (ZIP) [file pone.0265049.s007.zip › IHC/SF 7D.tif]

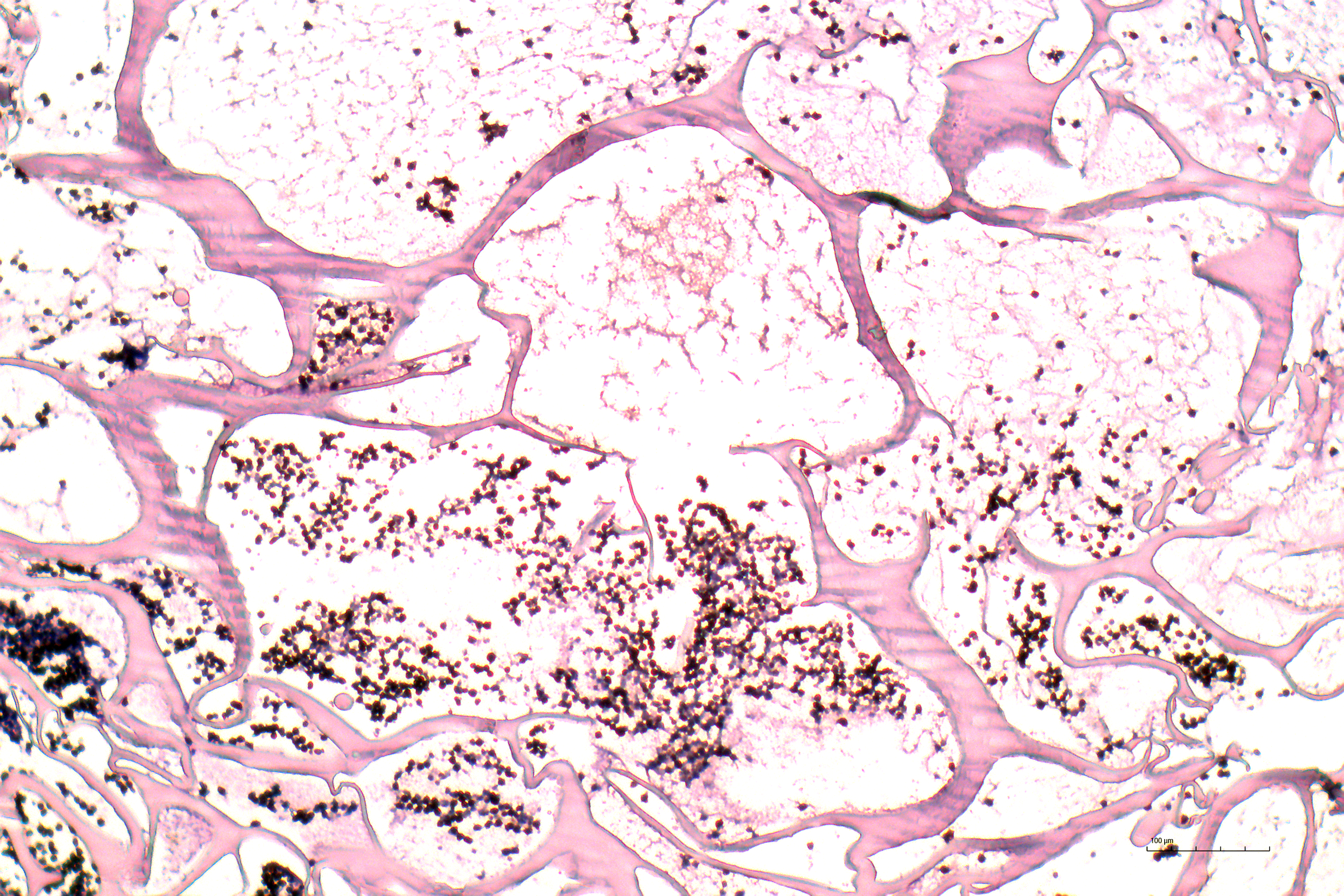

Supplement: S5 File — (ZIP) [file pone.0265049.s007.zip › IHC/SF+IT MSC-CM 3D.tif]

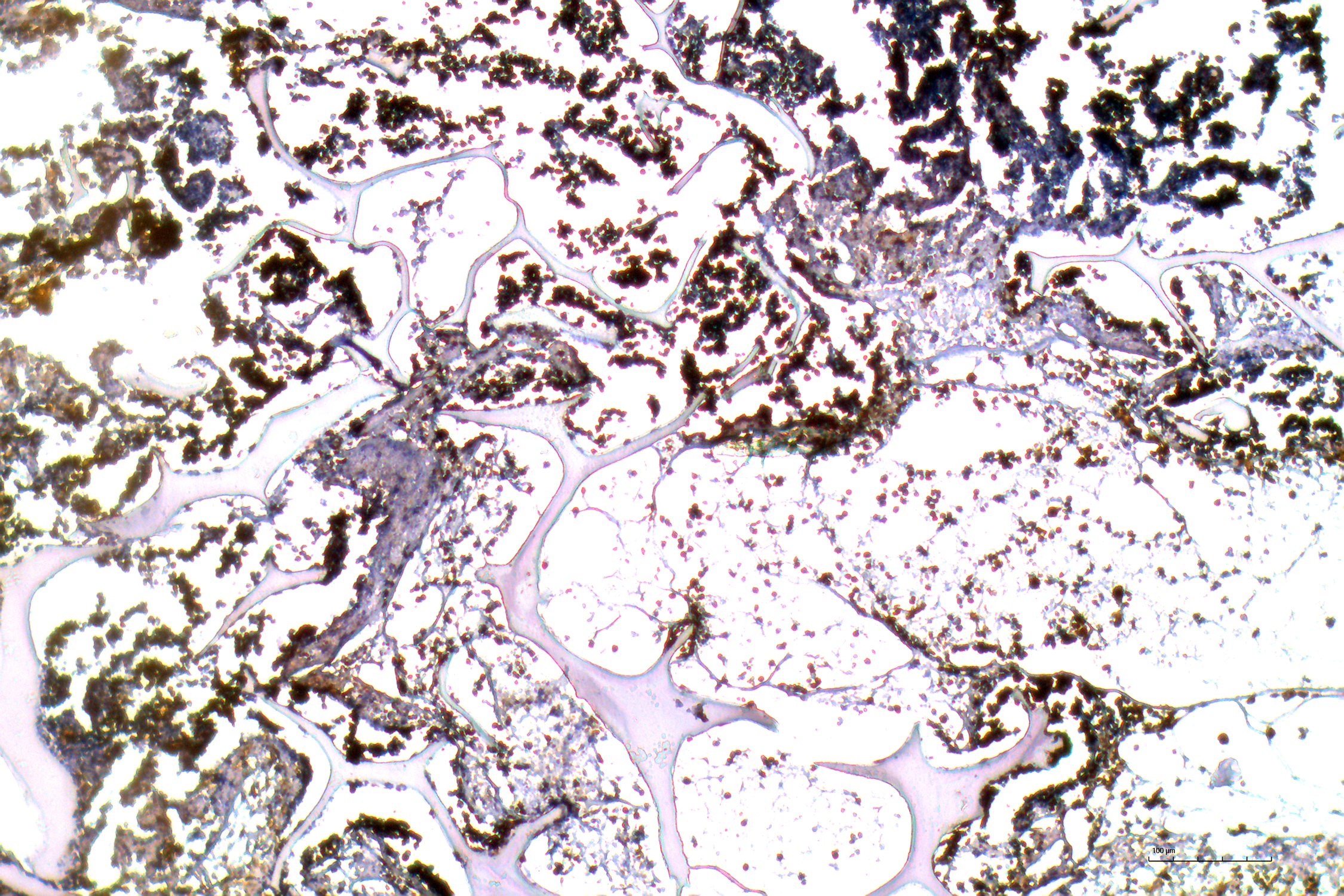

Supplement: S5 File — (ZIP) [file pone.0265049.s007.zip › IHC/SF+IT MSC-CM 7D.tif]

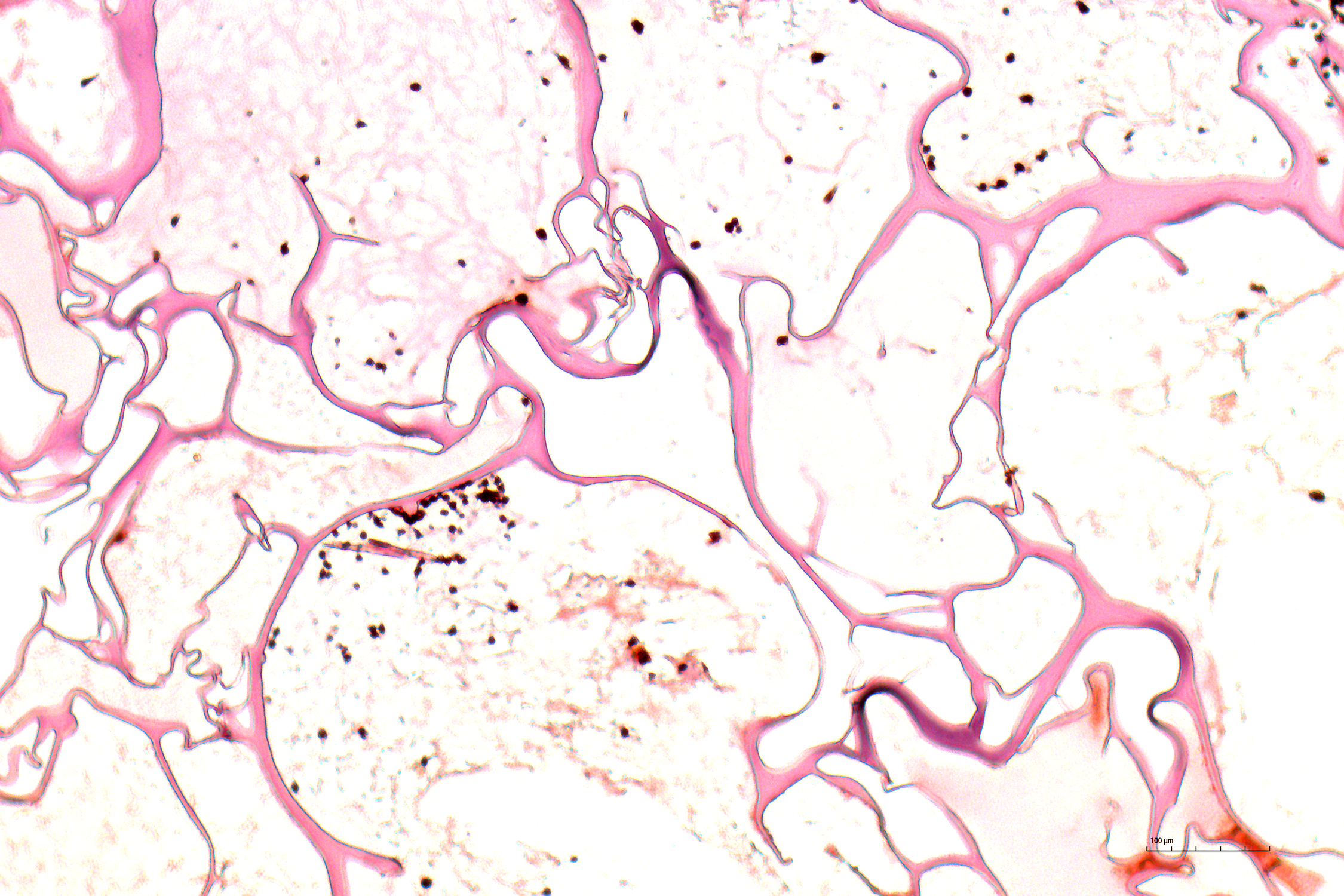

Supplement: S5 File — (ZIP) [file pone.0265049.s007.zip › IHC/SF+MSC-CM 3D.tif]

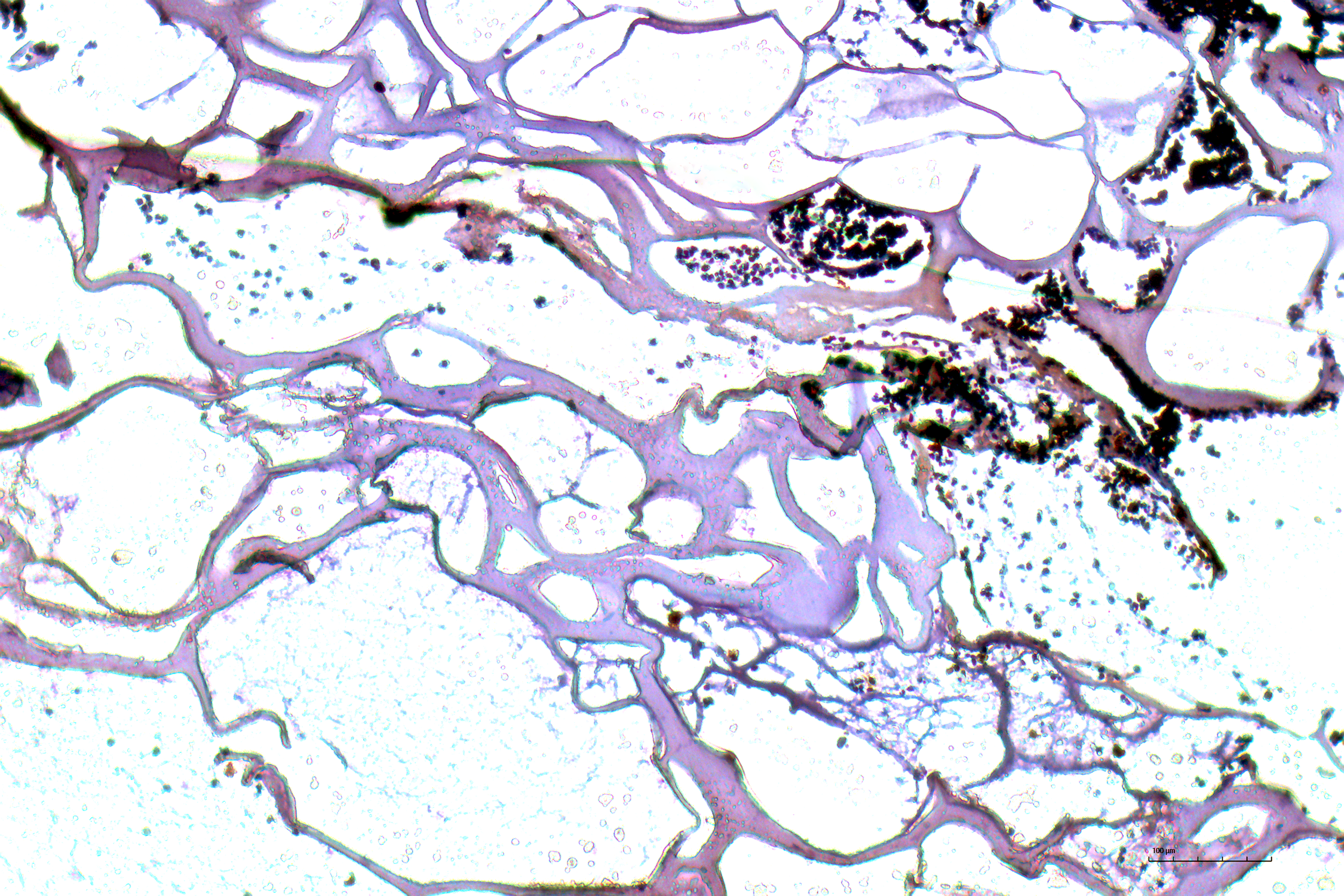

Supplement: S5 File — (ZIP) [file pone.0265049.s007.zip › IHC/SF+MSC-CM 7D.tif]

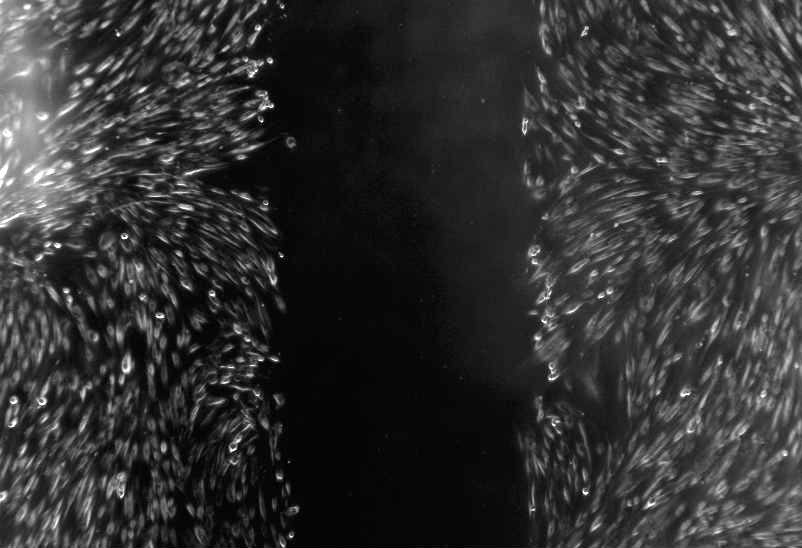

Supplement: S6 File — (ZIP) [file pone.0265049.s008.zip › Scrach assey/HDF DMEM 0h.tif]

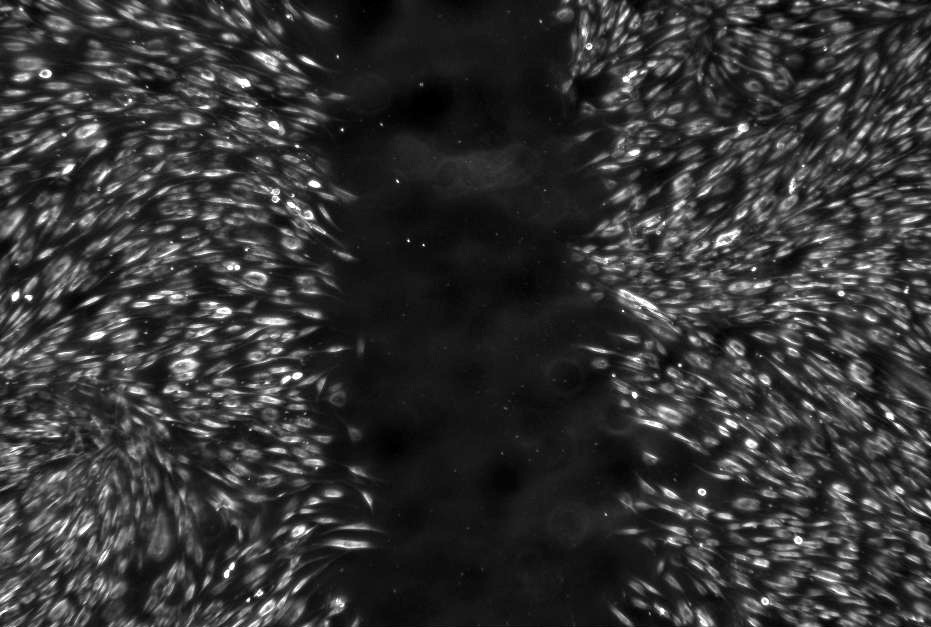

Supplement: S6 File — (ZIP) [file pone.0265049.s008.zip › Scrach assey/HDF DMEM 12h.tif]

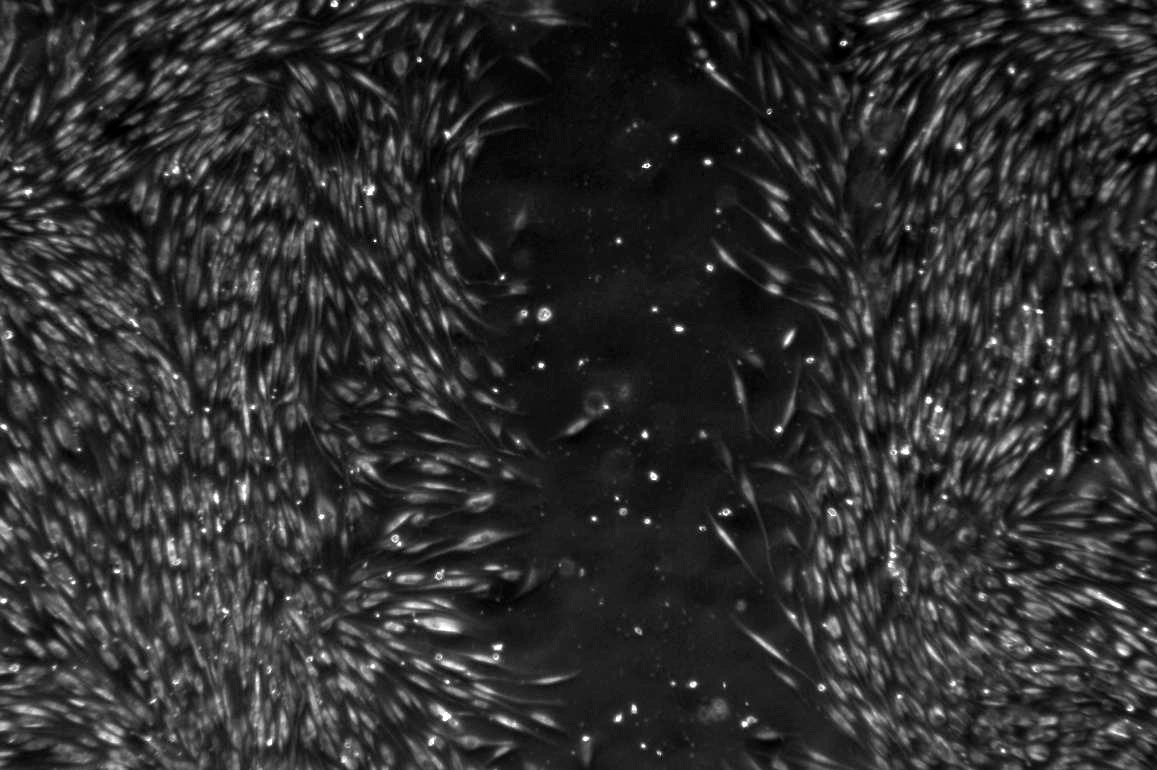

Supplement: S6 File — (ZIP) [file pone.0265049.s008.zip › Scrach assey/HDF DMEM 24h.tif]

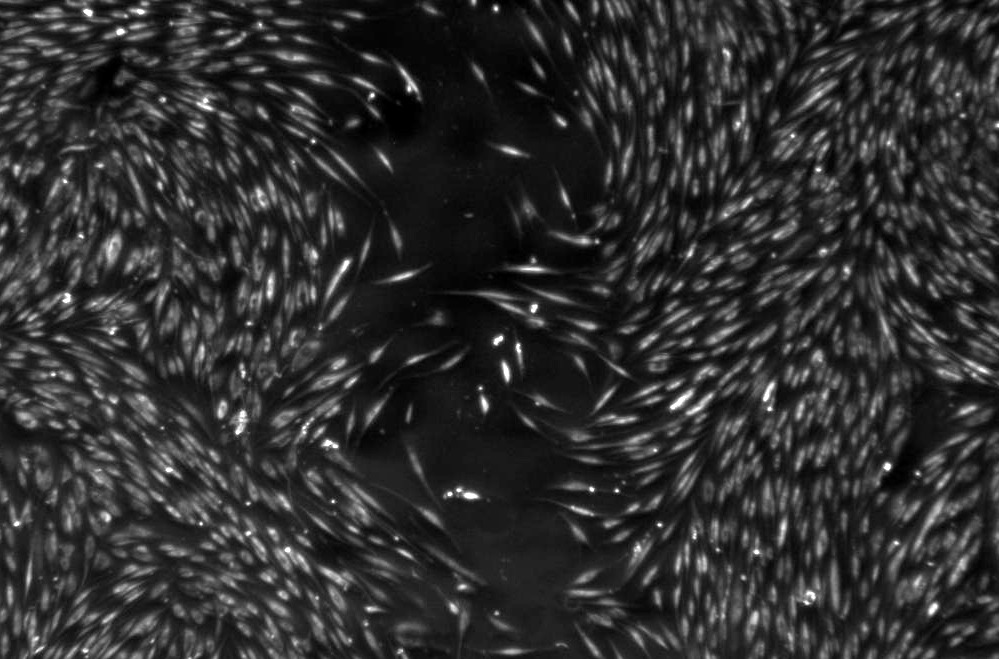

Supplement: S6 File — (ZIP) [file pone.0265049.s008.zip › Scrach assey/HDF DMEM 48h.tif]

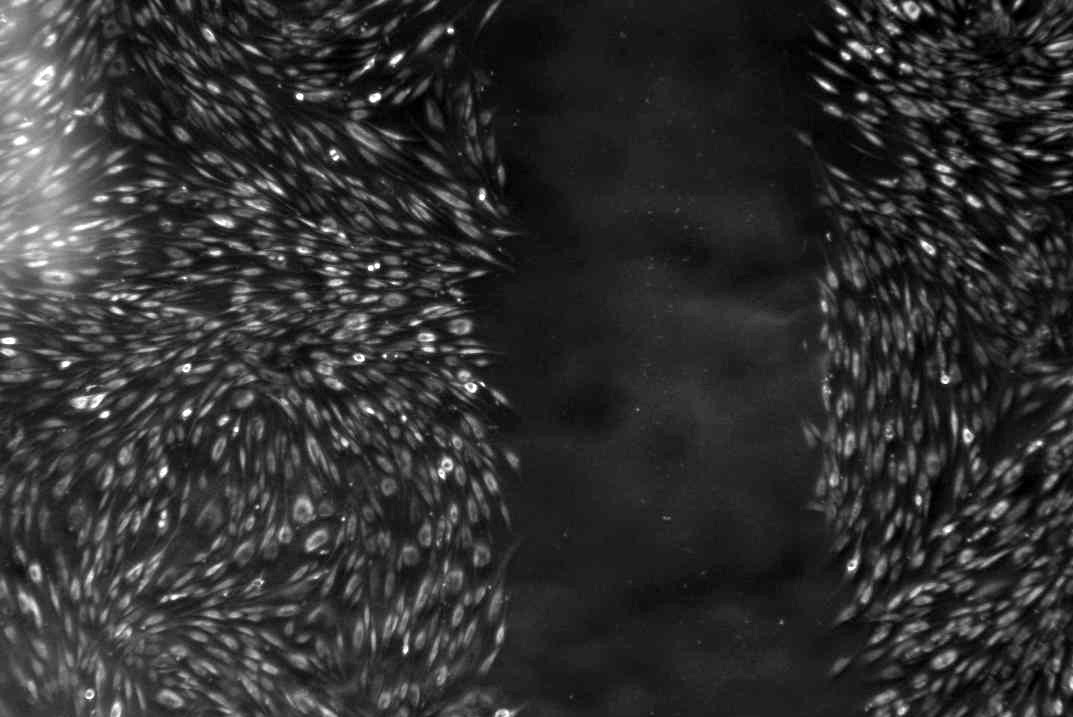

Supplement: S6 File — (ZIP) [file pone.0265049.s008.zip › Scrach assey/HDF DMEM 6h.tif]

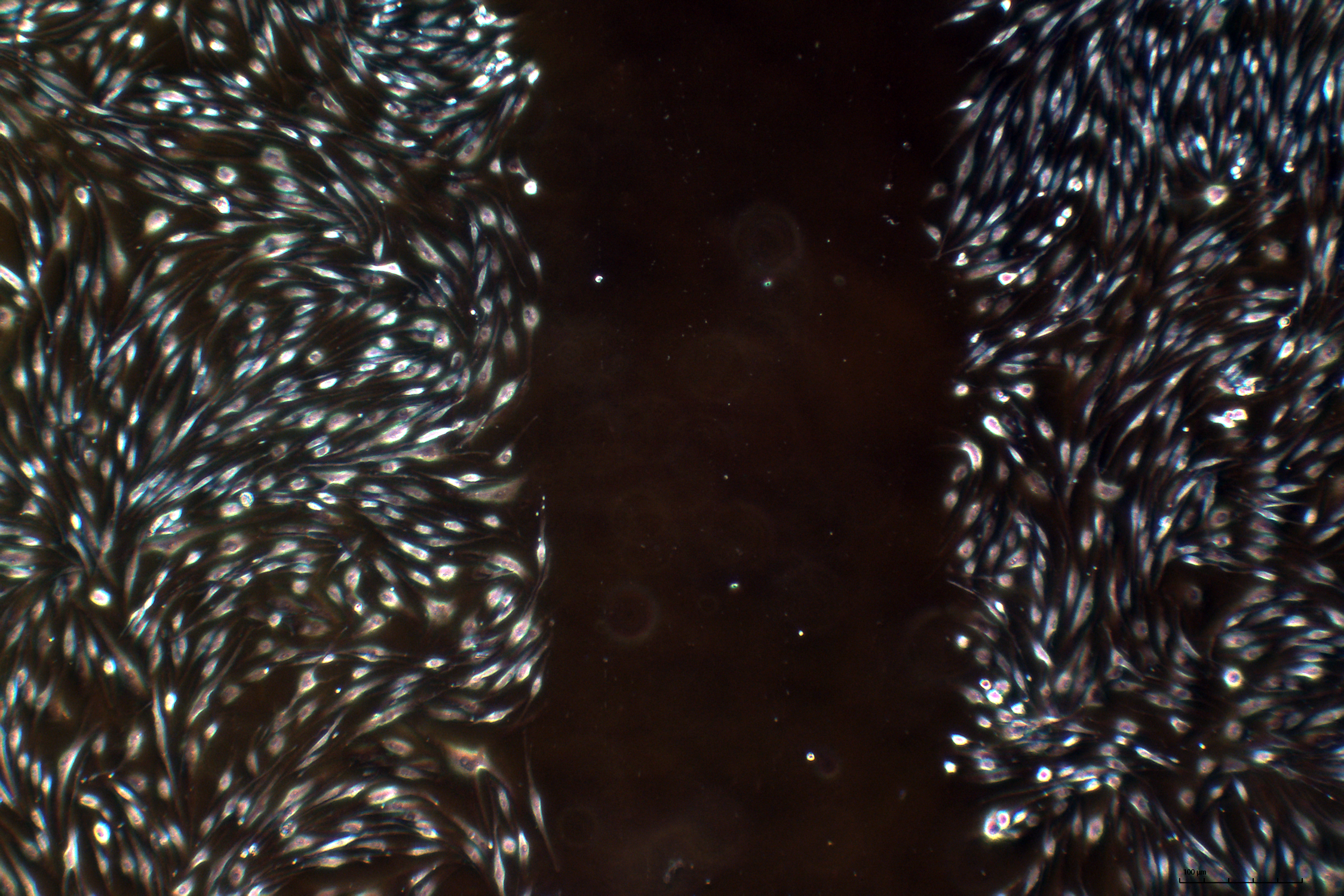

Supplement: S6 File — (ZIP) [file pone.0265049.s008.zip › Scrach assey/HDF IT MSC-CM 0h.tif]

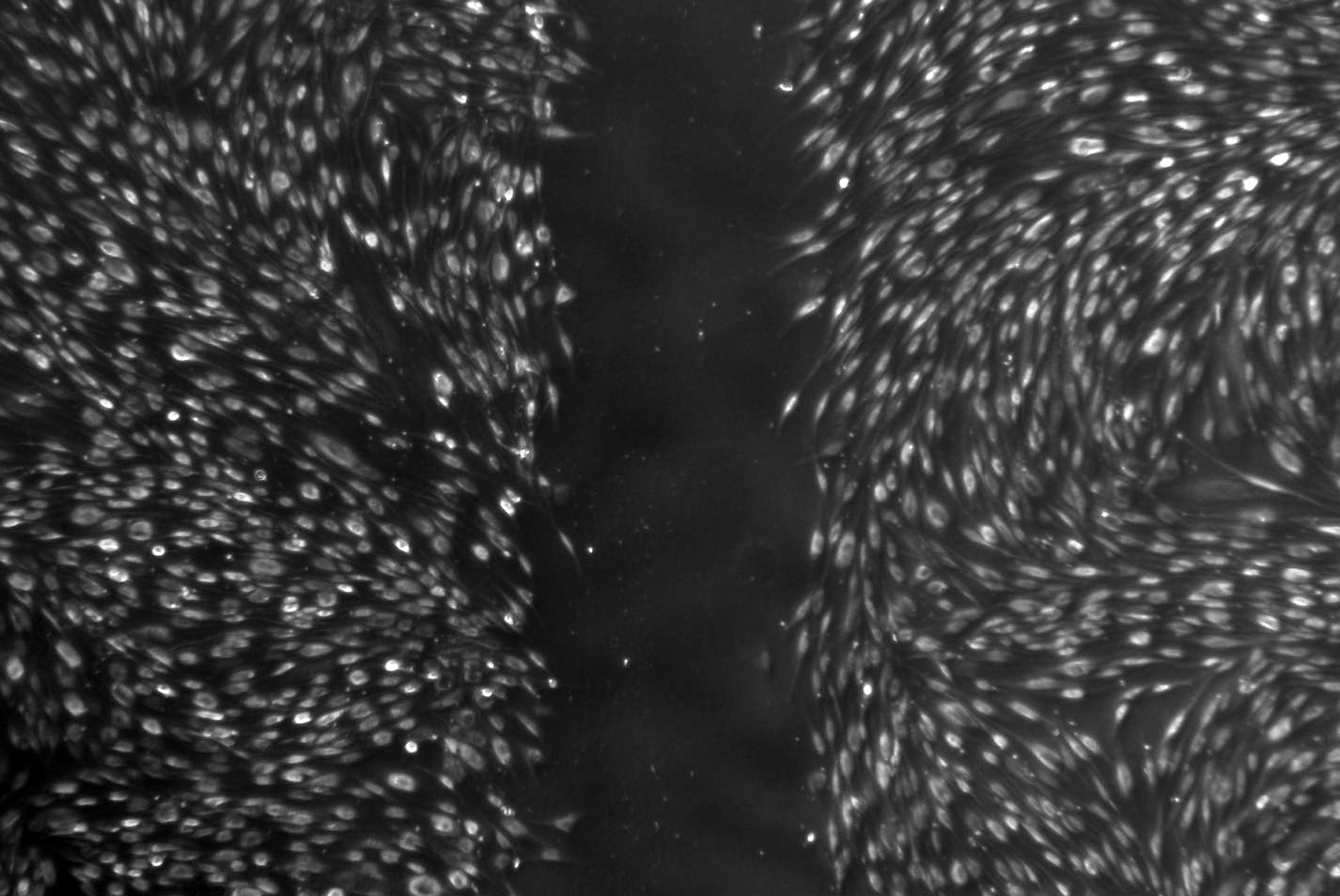

Supplement: S6 File — (ZIP) [file pone.0265049.s008.zip › Scrach assey/HDF IT MSC-CM 12h.tif]

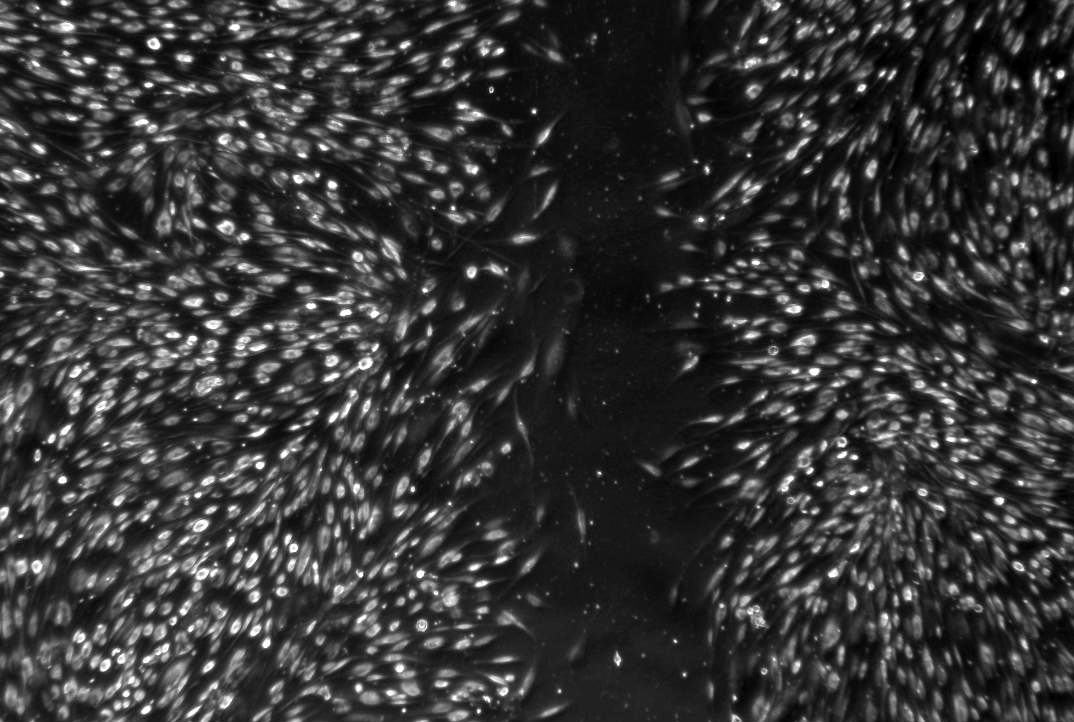

Supplement: S6 File — (ZIP) [file pone.0265049.s008.zip › Scrach assey/HDF IT MSC-CM 24h.tif]

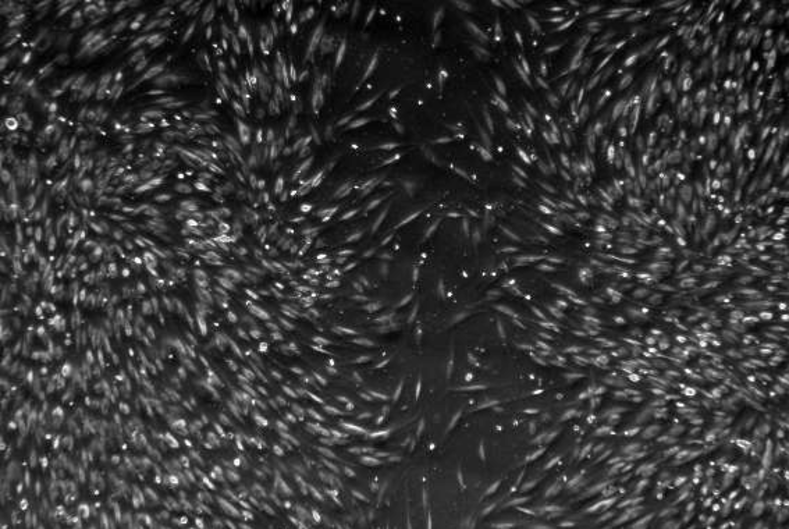

Supplement: S6 File — (ZIP) [file pone.0265049.s008.zip › Scrach assey/HDF IT MSC-CM 48h.tif]

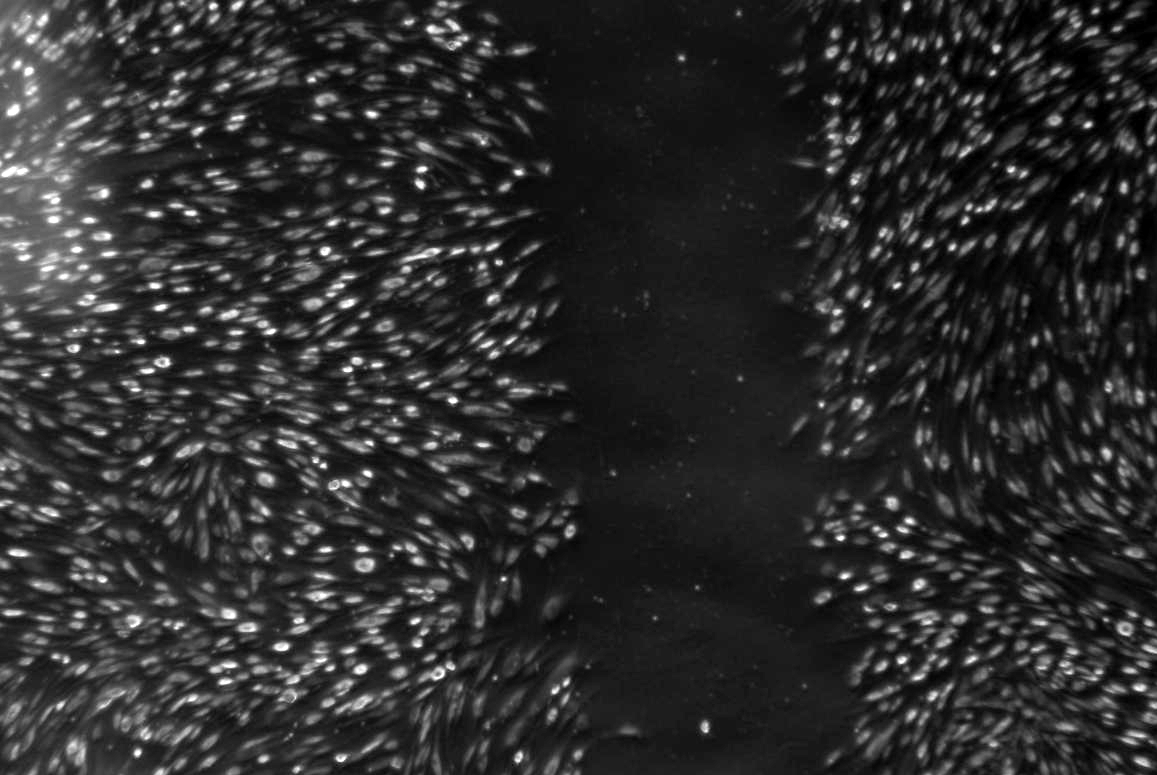

Supplement: S6 File — (ZIP) [file pone.0265049.s008.zip › Scrach assey/HDF IT MSC-CM 6h.tif]

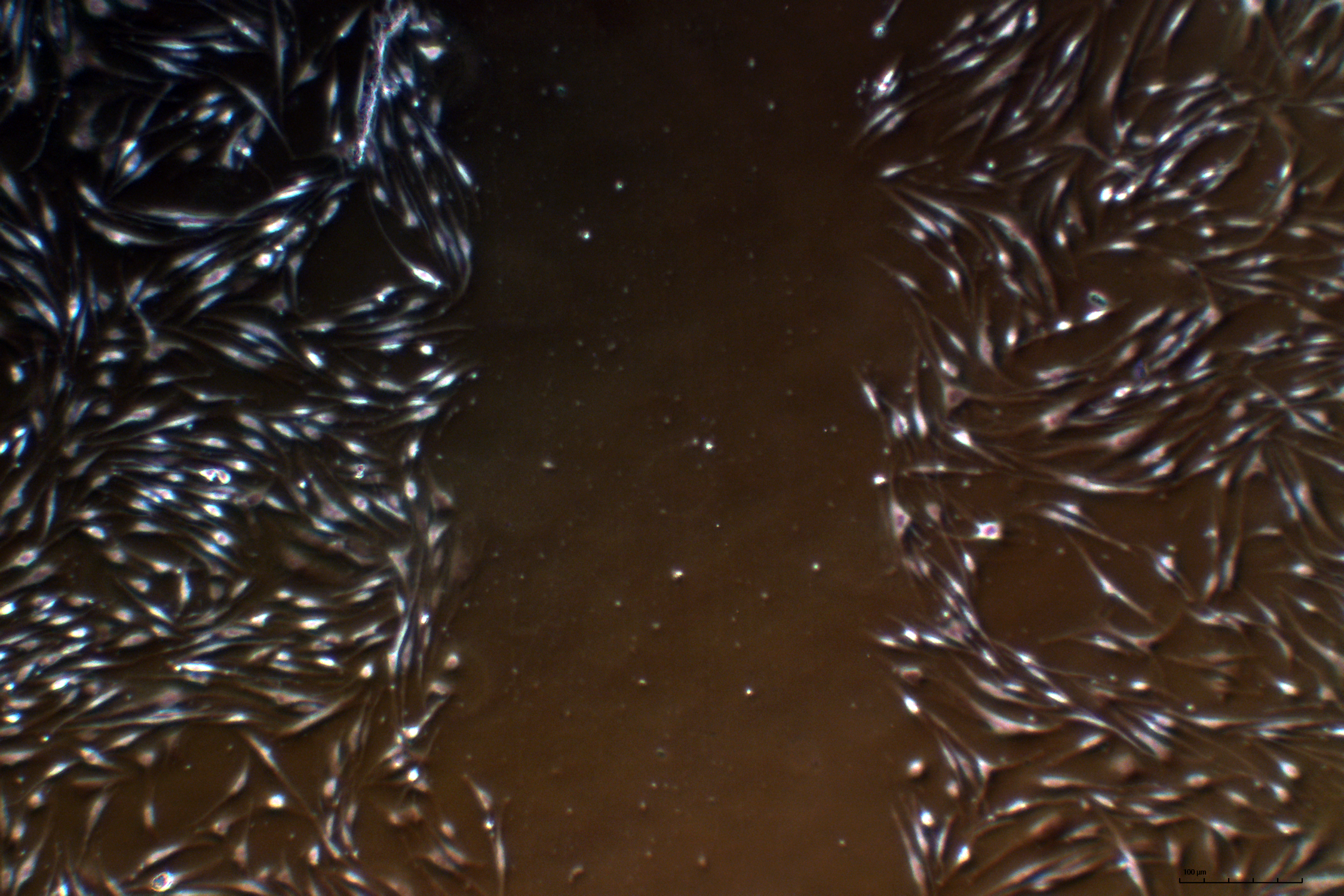

Supplement: S6 File — (ZIP) [file pone.0265049.s008.zip › Scrach assey/HDF MSC-CM 0h.tif]

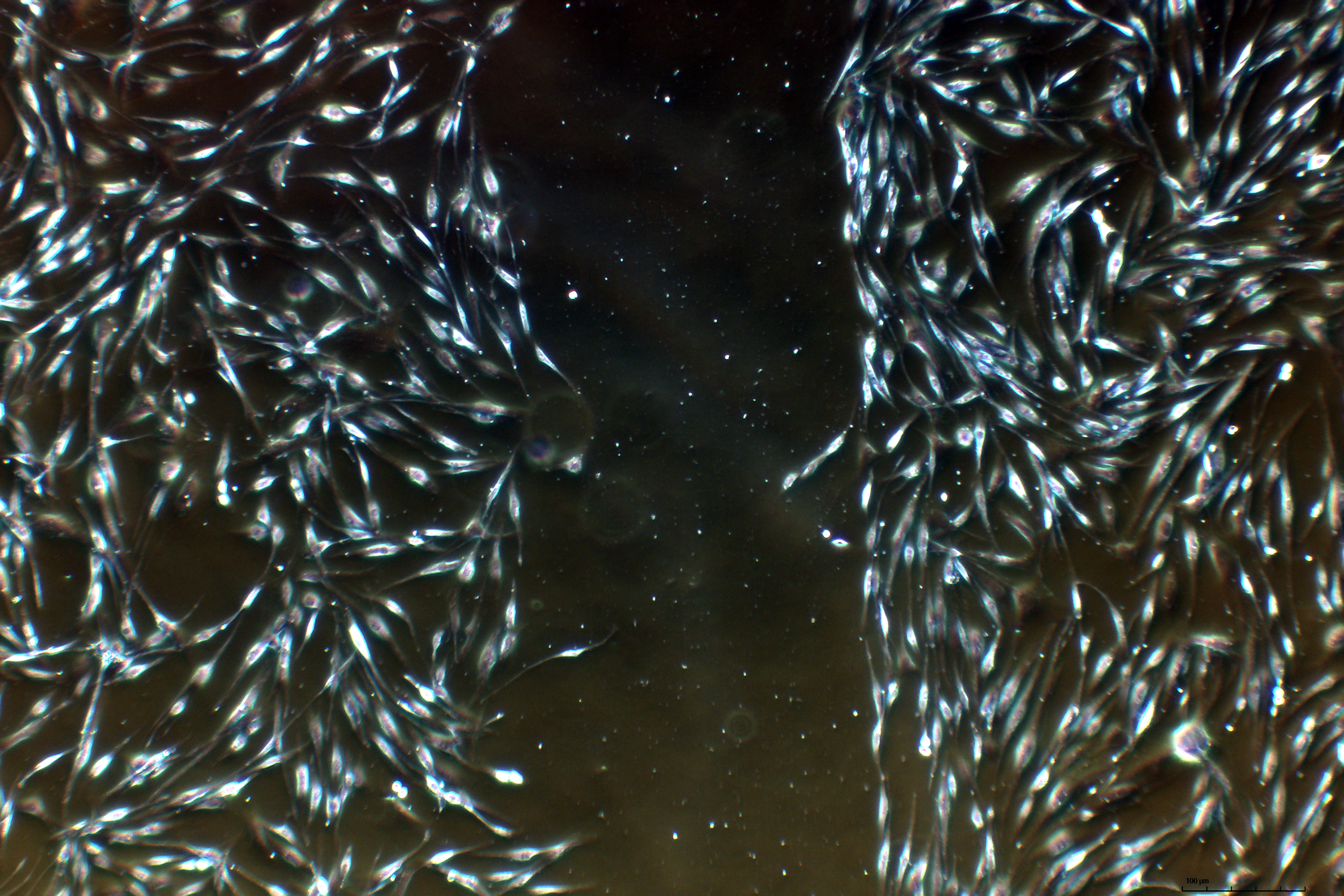

Supplement: S6 File — (ZIP) [file pone.0265049.s008.zip › Scrach assey/HDF MSC-CM 12h.tif]

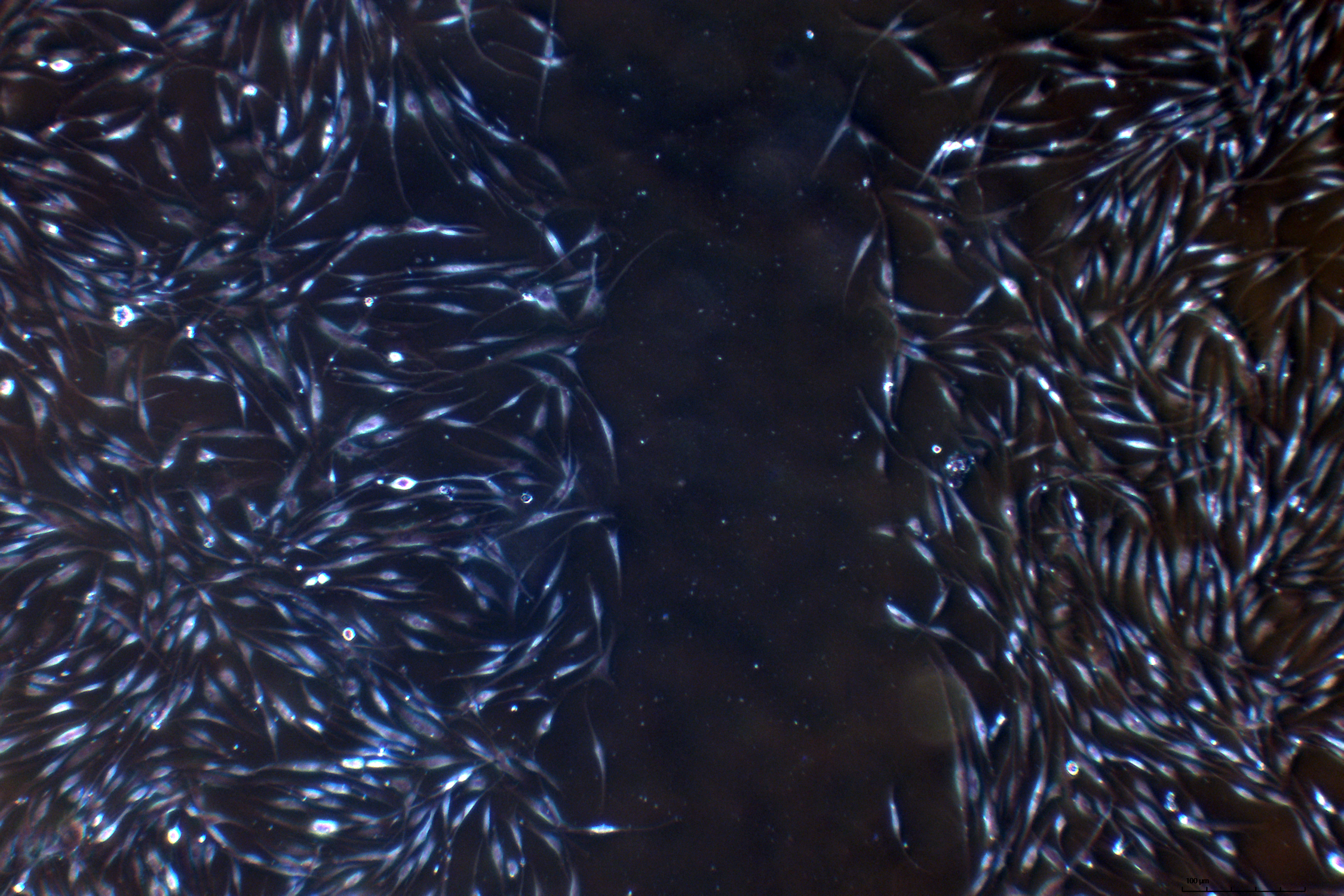

Supplement: S6 File — (ZIP) [file pone.0265049.s008.zip › Scrach assey/HDF MSC-CM 24h.tif]

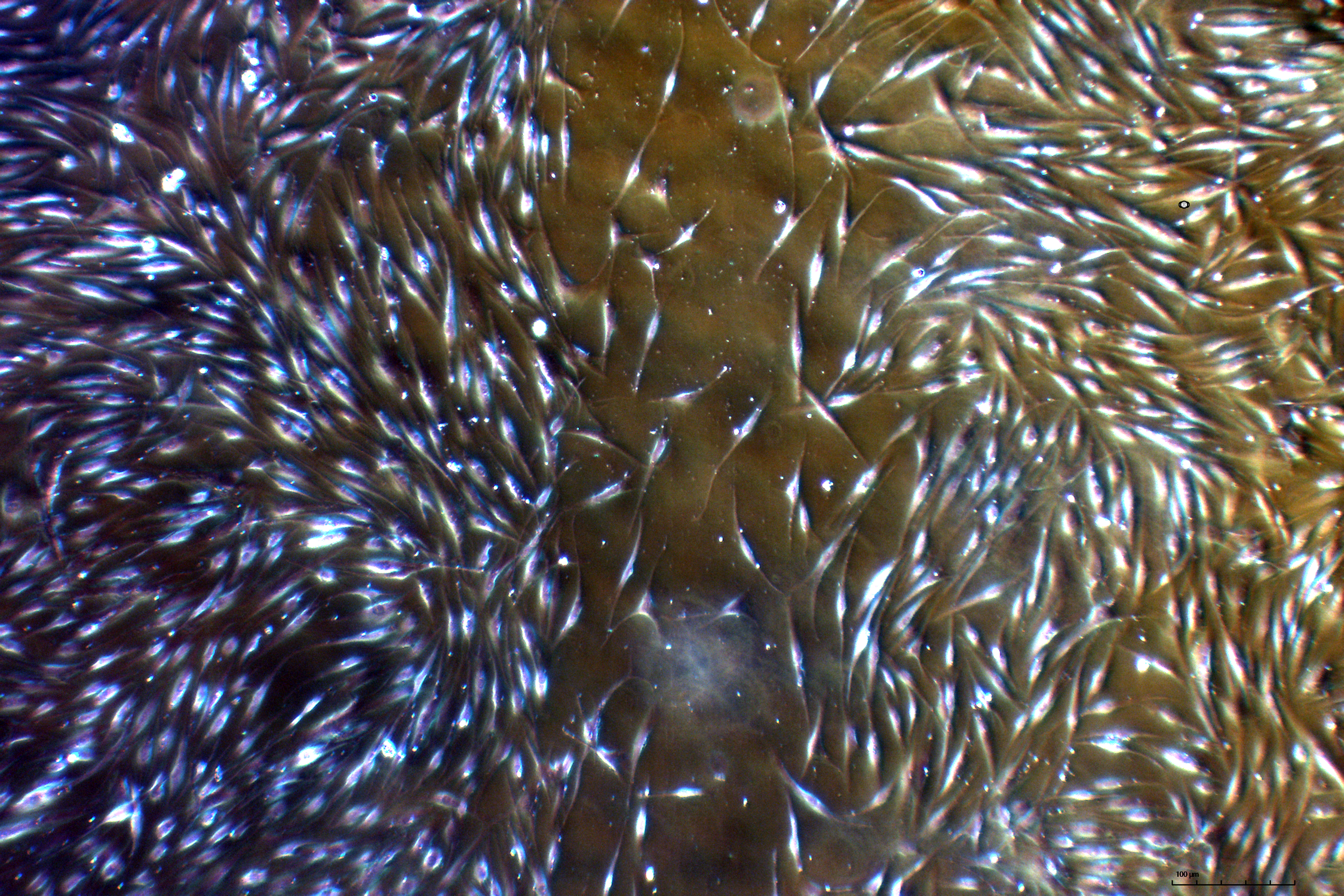

Supplement: S6 File — (ZIP) [file pone.0265049.s008.zip › Scrach assey/HDF MSC-CM 48h.tif]

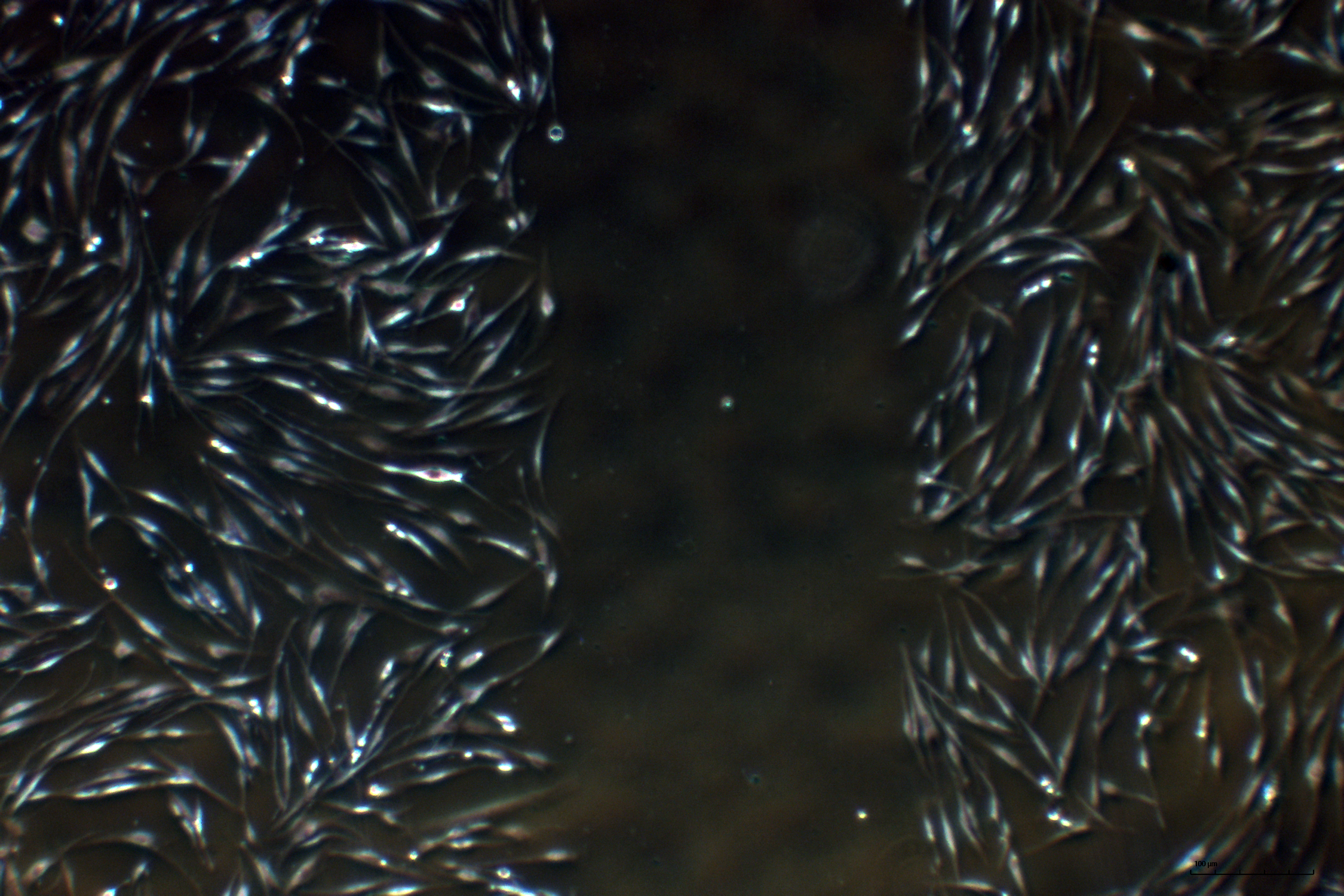

Supplement: S6 File — (ZIP) [file pone.0265049.s008.zip › Scrach assey/HDF MSC-CM 6h.tif]

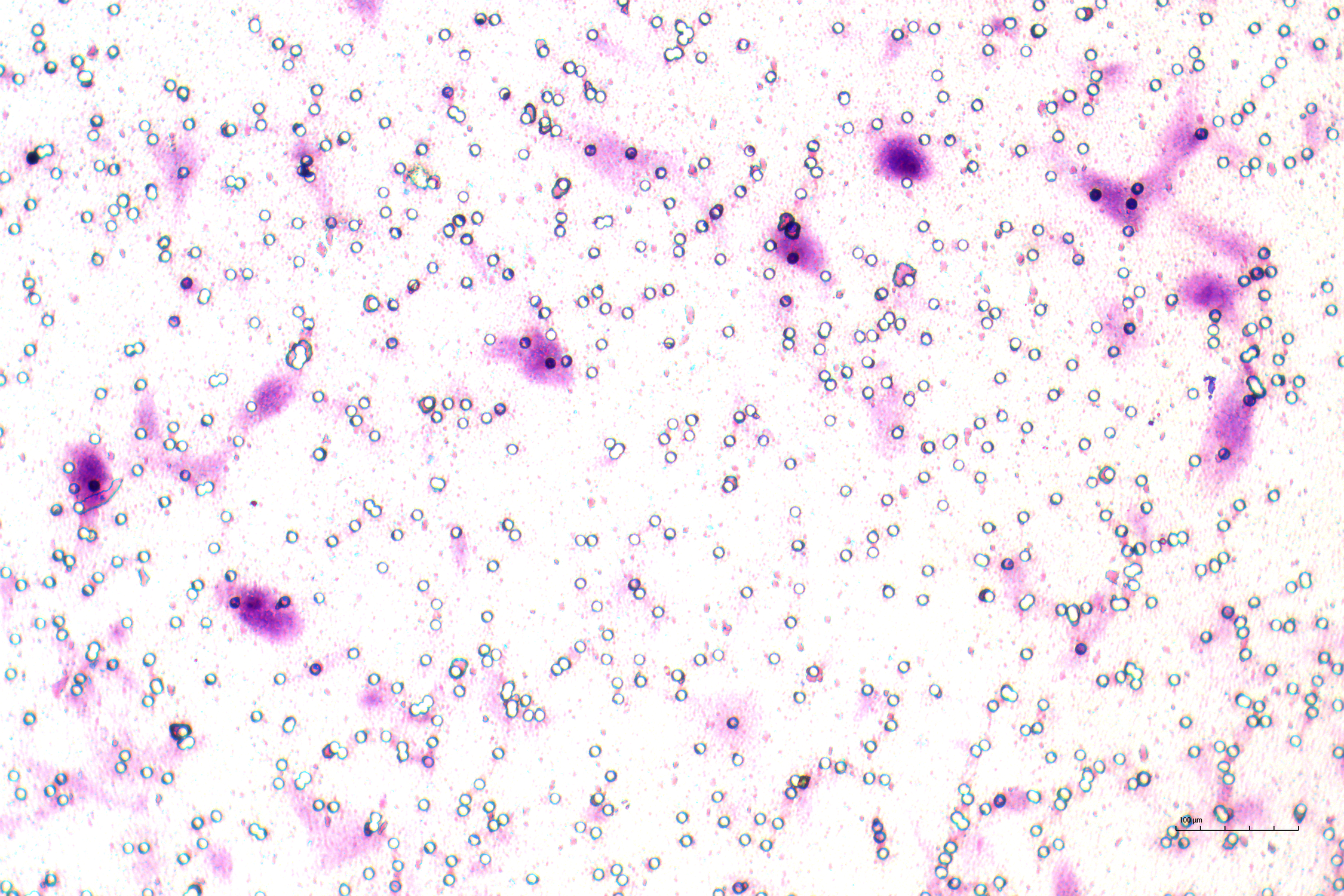

Supplement: S7 File — (ZIP) [file pone.0265049.s009.zip › Transwell assey/DMEM 10X.tif]

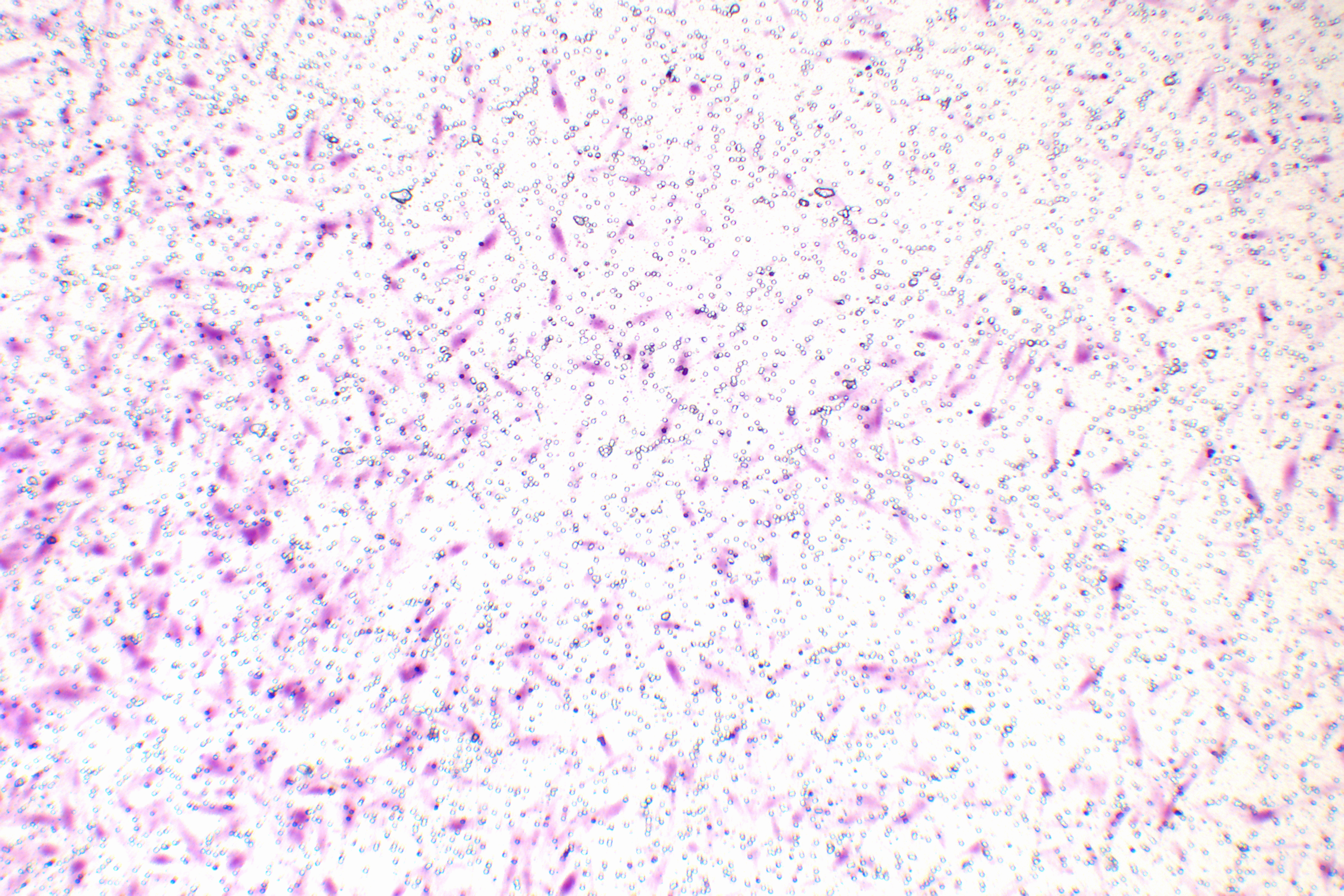

Supplement: S7 File — (ZIP) [file pone.0265049.s009.zip › Transwell assey/DMEM 4X.tif]

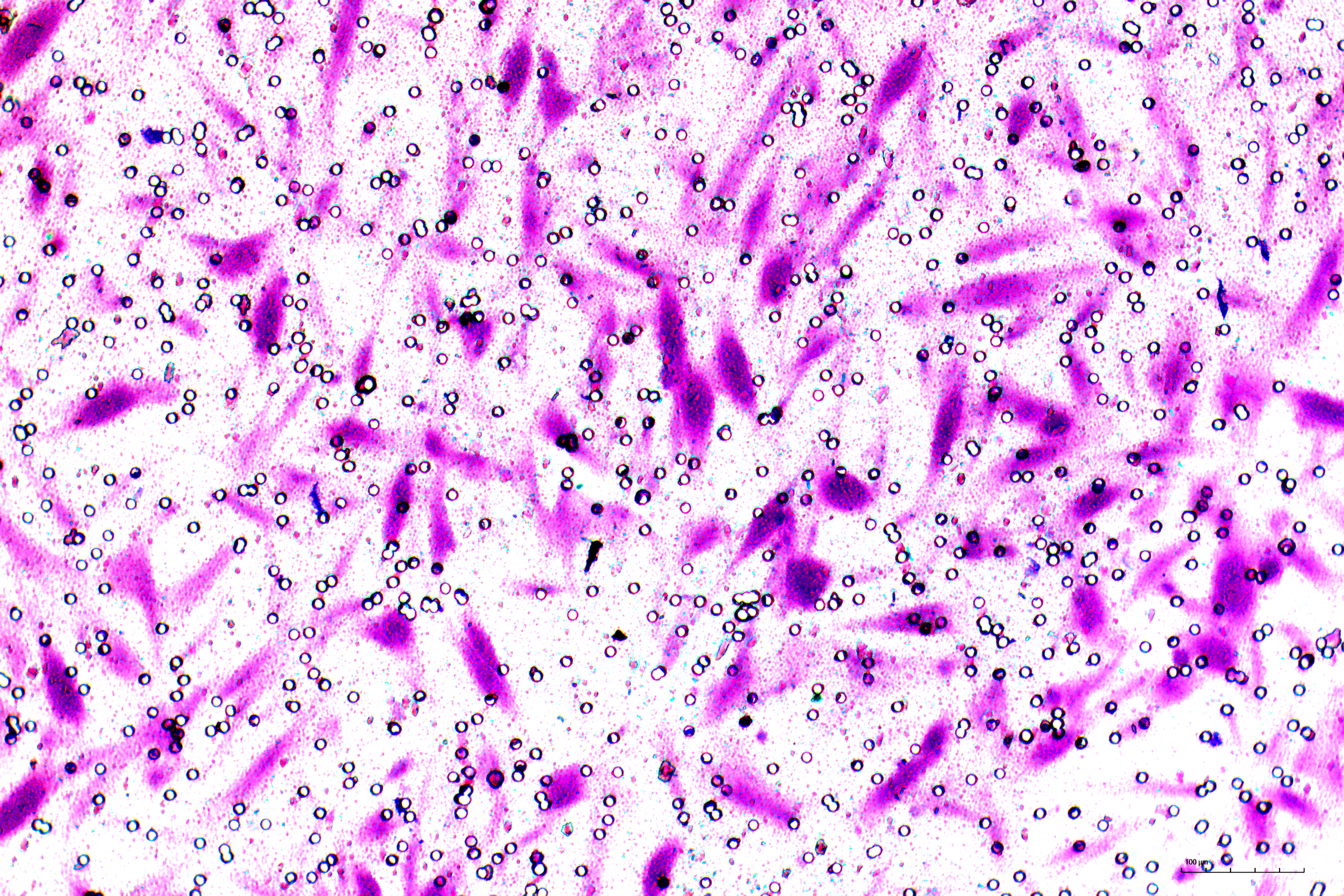

Supplement: S7 File — (ZIP) [file pone.0265049.s009.zip › Transwell assey/IT MSC-CM 10X.tif]

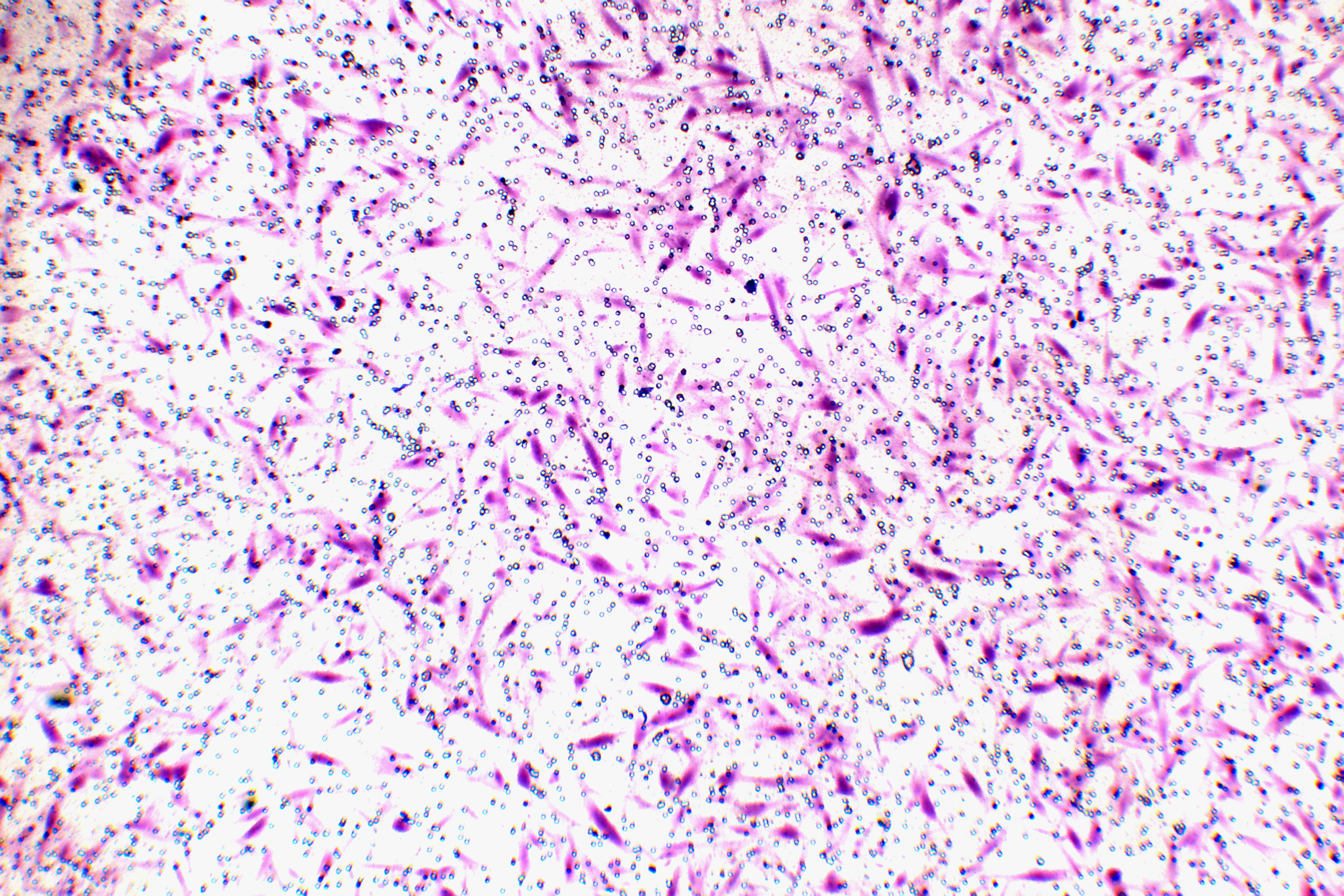

Supplement: S7 File — (ZIP) [file pone.0265049.s009.zip › Transwell assey/IT MSC-CM 4X.tif]

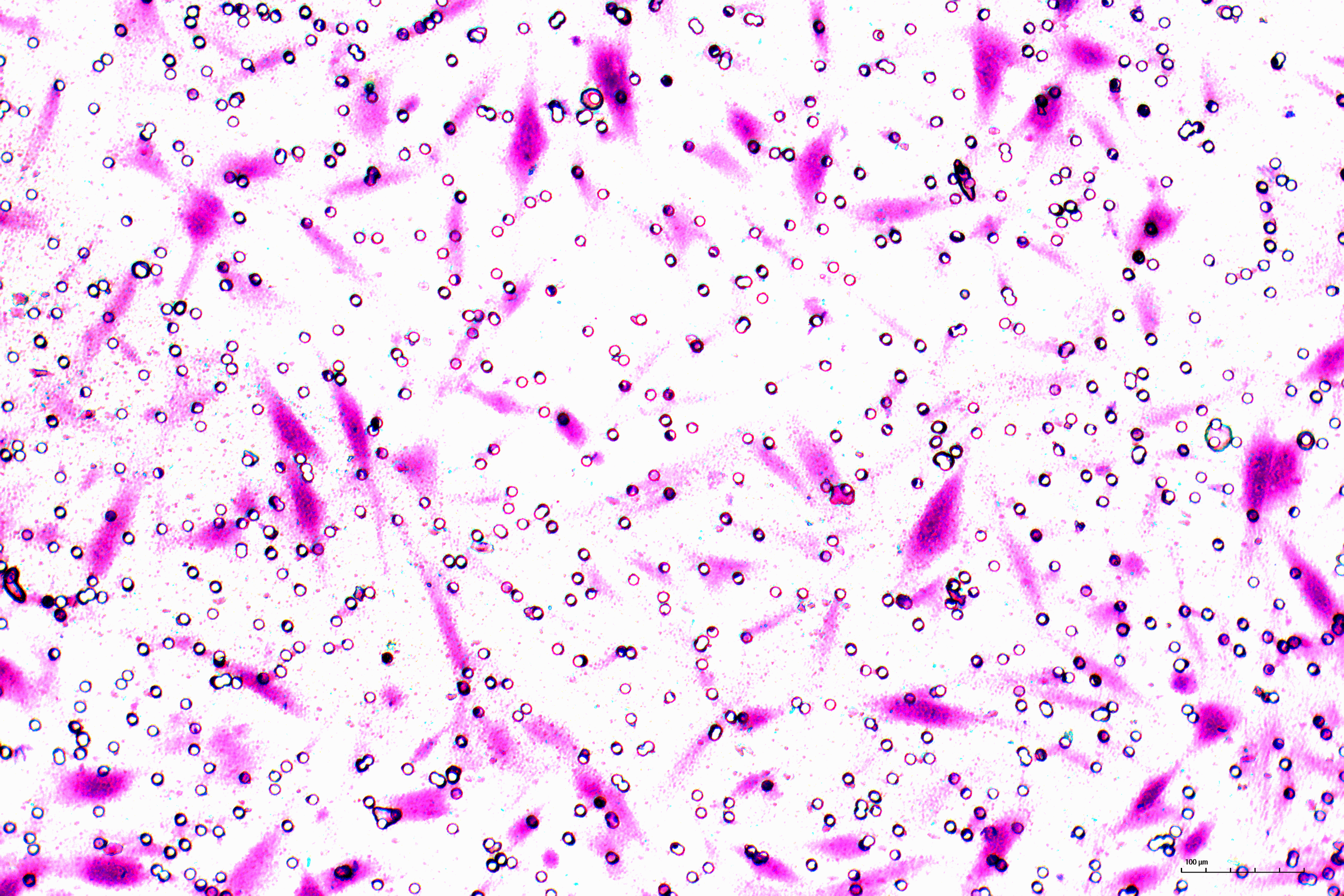

Supplement: S7 File — (ZIP) [file pone.0265049.s009.zip › Transwell assey/MSC-CM 10X.tif]

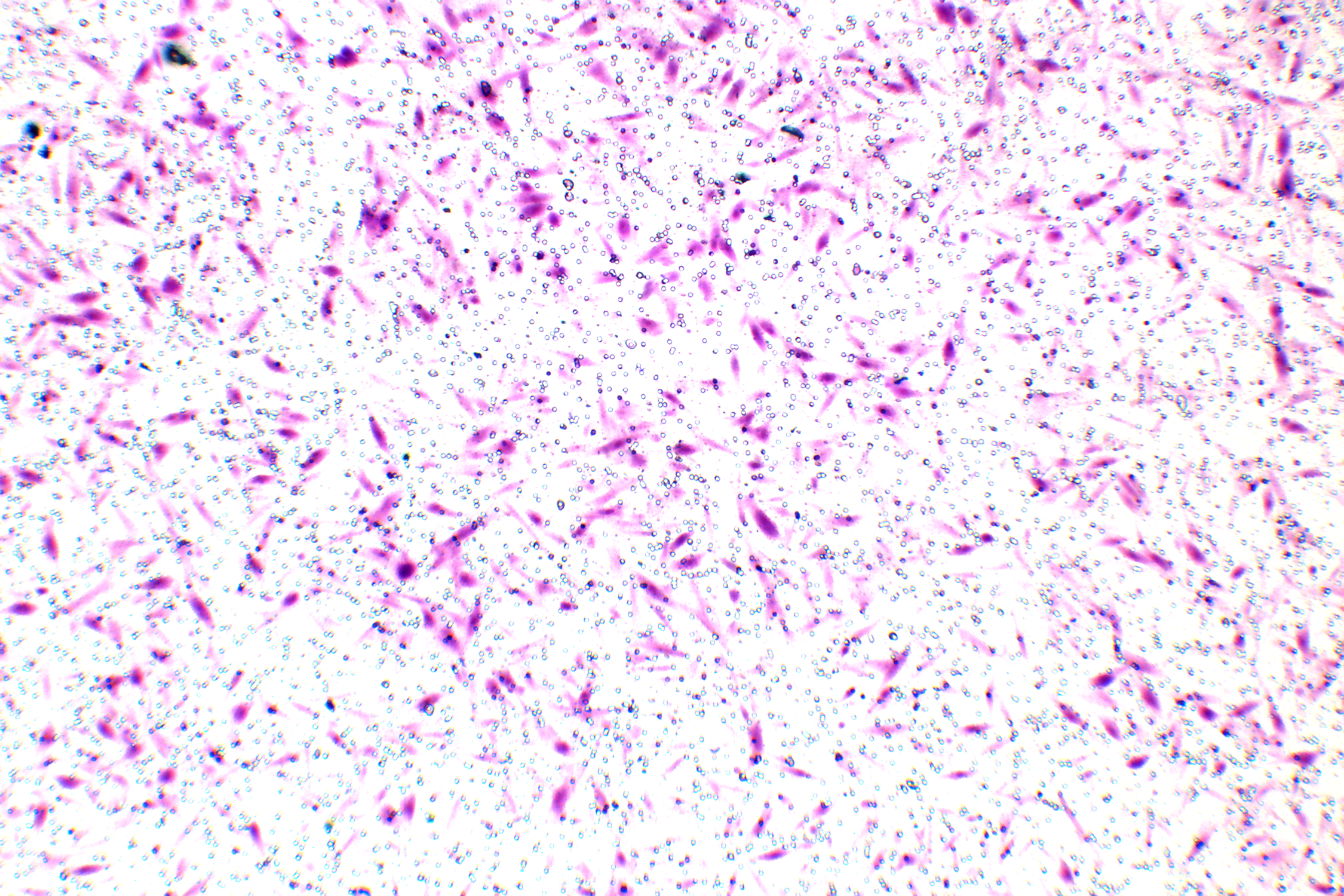

Supplement: S7 File — (ZIP) [file pone.0265049.s009.zip › Transwell assey/MSC-CM 4X.tif]
