# Supplementary material for: The conditioned medium from mesenchymal stromal cells pretreated with proinflammatory cytokines promote fibroblasts migration and activation
Source: PLoS One. 2022 Apr 11;17(4):e0265049. doi: 10.1371/journal.pone.0265049 (PMC9000110; doi:10.1371/journal.pone.0265049)
Supplement: Attachment — Submitted filename: Response to Reviewers.docx [file pone.0265049.s010.docx]

**Response to Reviewers**

Dear reviewer:

I am very grateful to your comments for the manuscript. According with your advice, we amended the relevant part in manuscript. Some of your questions were answered below:

Part A (Reviewer 1):

**1.The reviewer’s comment:** Why was the conditioned medium removed at one time only (12h) after the treatment with IT? The authors should give an explanation for this choice or perform experiments also at different times to evaluate if the production of factors by MSCs changes and has different effects on HDFs.

**The author’s answer:** The conditioned medium of stem cells conditioned medium was extracted by incubating stem cells after IT stimulation for 12 hours using Dulbecco’s modified Eagle’s medium (DMEM) without the nutritional support of fetal bovine serum (FBS). Considering the possible effect of starvation stress on stem cells paracrine secretion if the DMEM incubates the stem cells for too long, therefore, we used a conditioned medium that was extracted after 12h incubation.

**2.The reviewer’s comment:** Moreover, qRT-PCR should be performed also at longer times (almost 48h and 7 days). Gene expression should be confirmed by protein production.

**The author’s answer:** The conditioned medium used in this study was a DMEM extract without nutritional support from FBS. During the in vitro experiments to investigate the effect of each group of conditioned media on HDF, especially the negative control group (DMEM), considering the absence of nutritional support from FBS and necessary bioactive factors, prolonged incubation of HDF with conditioned media, starvation stress may have an effect on HDF biological activity. Therefore, conditioned medium was used to incubate HDF for 24h in this study. In addition, the protein expression corresponding to the gene expression has been added to this paper using Western blot and ELISA assays.

**3.The reviewer’s comment:** In Paragraph 3.5 authors refer to figure 7 and 8 but all the images are in figure 7. Please, change it.

**The author’s answer:** Yes, we have changed it.

**4.The reviewer’s comment:** Paragraph 3.6 should be moved before in vivo experiments, or the results should be explained in a different figure (figure 8). Moreover, Authors assessed that “HDFs stimulated by IT MSC-CM may function through high expression of PDGF-BB”. Only ELISA assay is not sufficient to assert it, but silencing is necessary. Therefore, Auhtors should change this statement as a guess.

**The author’s answer:** Yes, We have made changes according to your suggestions.

Part B (Reviewer 2):

**1.The reviewer’s comment:** Molecular data must be confirmed by corresponding protein levels.

**The author’s answer:** Yes, the protein expression corresponding to the gene expression has been added to this paper using Western blot and ELISA assays in Fig 4.

**2.The reviewer’s comment:** Assays of gene function loss must be performed in order to confirm the results regarding PDGF-BB expression.

**The author’s answer:** In the present study, we speculated based on ELISA results that IT MSC-CM may play a role in promoting the activation and migration of HDFs through high level of PDGF-BB, which just a guess. A more rigorous and comprehensive in vivo and in vitro validation will be carried out in the next research work of our group. We have changed the statement in this paper as a guess.

**3.The reviewer’s comment:** In addition, the Authors should obtain conditioned medium culturing the cells at longer times. In my opinion, 12 hours are few. It would be interesting to evaluate whether there are different effects on fibroblasts due to a different amount of specific factors secreted by MSC.

**The author’s answer:** The conditioned medium of stem cells conditioned medium was extracted by incubating stem cells after IT stimulation for 12 hours using Dulbecco’s modified Eagle’s medium (DMEM) without the nutritional support of fetal bovine serum (FBS). Considering the possible effect of starvation stress on stem cells paracrine secretion if the DMEM incubates the stem cells for too long, therefore, we used a conditioned medium that was extracted after 12h incubation.

**4.The reviewer’s comment:** The Authors must check the images showed in the figures and their description in Results section. They are not always corresponding.

**The author’s answer:** Yes, We have made changes according to your suggestions.
